# Clinical Efficacy and Real-World Effectiveness of Fabry Disease Treatments: A Systematic Literature Review

**DOI:** 10.3390/jcm14145131

**Published:** 2025-07-18

**Authors:** Ana Jovanovic, Eve Miller-Hodges, Felicia Castriota, Obaro Evuarherhe, Olulade Ayodele, Derralynn Hughes, Guillem Pintos-Morell, Roberto Giugliani, Sandro Feriozzi, Csaba Siffel

**Affiliations:** 1 The Mark Holland Metabolic Unit, Northern Care Alliance NHS Foundation Trust, Salford M6 8HD, UK; 2 Centre for Cardiovascular Science, Queen’s Medical Research Institute, University of Edinburgh, Edinburgh EH16 4TJ, UK; 3 Global Evidence and Outcomes, Data and Quantitative Sciences Institute, Takeda Development Center Americas, Inc., Cambridge, MA 02142, USA; 4 Oxford PharmaGenesis Ltd., Tubney OX13 5QJ, UK; 5 Lysosomal Storage Disorders Unit, Royal Free London NHS Foundation Trust, University College London, London WC1E 6BT, UK; 6 Vall d’Hebron Institute of Research, Vall d’Hebron Barcelona Hospital Campus, MPS-Lisosomales Medical Committee, 08035 Barcelona, Spain; 7 Department of Genetics, Federal University of Rio Grande do Sul (UFRGS), Medical Genetics Service, Hospital de Clinicas de Porto Alegre (HCPA), Dasa Genomics, and Casa dos Raros, Porto Alegre 90610-261, Brazil; 8 Nephrology Department, University Campus Bio-Medico, University of Rome, 00128 Rome, Italy; 9 College of Allied Health Sciences, Augusta University, Augusta, GA 30912, USA

**Keywords:** agalsidase alfa, agalsidase beta, efficacy, effectiveness, Fabry disease, lysosomal storage disease, migalastat

## Abstract

**Objectives:** This systematic literature review aimed to identify studies assessing the clinical efficacy and real-world effectiveness of current and emerging treatments for Fabry disease. **Methods:** Searches of the MEDLINE, EMBASE, and Cochrane library databases, as well as relevant congress proceedings, were conducted to identify publications reporting on studies in patients of any age, sex, race, or ethnicity who received any approved or experimental treatment for Fabry disease, published before 17 June 2024. **Results:** Of 1881 publications screened, 234 reported data on renal, cardiac, cerebrovascular, and disease severity outcomes from 225 studies. The majority of reported studies were observational in nature (*n* = 150; 67%) and involved only adults (*n* = 172; 74%). Study designs and patient populations were highly heterogeneous, and cross-study conclusions about the effectiveness of different therapies could not be made. Enzyme replacement therapy (ERT) with agalsidase alfa or agalsidase beta stabilized renal function and cardiac structure in patients with Fabry disease. Early initiation of ERT in childhood or young adulthood was associated with better renal and cardiac outcomes than treatment initiation at a later age. The small number of comparator studies of agalsidase alfa and agalsidase beta suggested similar efficacy. Patients treated with migalastat and pegunigalsidase alfa also maintained stable renal function and cardiac structure. **Conclusions:** Overall, current treatments slow the progression of renal and cardiac decline in patients with Fabry disease. Large cohort studies with long-term follow-up and baseline stratification based on clinical phenotype are needed to address evidence gaps and provide clinicians with robust data to inform treatment decisions.

## 1. Introduction

Fabry disease (FD) is a rare, X-linked lysosomal storage disease caused by variants in the α-galactosidase A gene (*GLA*) that result in deficient α-galactosidase A enzyme (α-Gal A) activity [1]. This deficiency results in the systemic accumulation of glycosphingolipids in lysosomes and leads to heterogeneous, progressive manifestations, including renal failure, cardiomyopathy, and cerebrovascular disease, together with a significant reduction in life expectancy [1,2,3,4]. The severe ‘classic’ FD phenotype presents with multisystemic involvement, whereas ‘non-classic’ or ‘late-onset’ FD phenotypes may be restricted to a single organ, usually the heart [5,6]. The rate of disease progression can vary substantially among patients [7].

Treatment for FD involves the replacement or augmentation of α-Gal A function. Several approved therapies are available, including enzyme replacement therapy (ERT) with agalsidase alfa [8], agalsidase beta [9,10], or more recently, pegunigalsidase alfa (a pegylated form of the α-Gal A enzyme) [11,12], and oral chaperone therapy with migalastat [13,14] for patients with amenable *GLA* variants. Beyond these therapies, research into the treatment of FD continues to advance, with several emerging therapies under investigation in clinical trials. These include gene therapy to address the genetic defect responsible for the α-Gal A deficiency, and substrate reduction therapy, a novel approach intended to modulate the metabolic pathways involved in glycosphingolipid synthesis [15,16].

The purpose of this systematic literature review (SLR) was to identify and summarize the literature relating to the clinical efficacy (based on clinical trial data) and real-world effectiveness (based on data from observational studies) of available and emerging treatments for FD. Specific topics of interest were the impact of treatment on cardiovascular, renal, cerebrovascular, and disease severity outcomes.

## 2. Methods

### 2.1. Search Strategy

The SLR search strategy and reporting was fully compliant with the 2020 Preferred Reporting Items for Systematic Review and Meta-Analysis (PRISMA) guidelines [17]. The protocol was prospectively registered in the International Prospective Register of Systematic Reviews in Health and Social Care (PROSPERO, ID number CRD42023394203) on 26 January 2023. Searches of MEDLINE (in-process and other non-indexed citations), EMBASE, and the Cochrane library databases were conducted on 28 November 2022 and repeated using the same criteria on 17 June 2024. Search strings are shown in Supplementary Table S1. The population of interest was patients with FD of any age, sex, race, or ethnicity who received any approved or experimental disease-specific treatment for FD. Searches were restricted to English but were not limited by year or region. Case reports were excluded. Proceedings from the following key congresses (from 2020 to 2024) were also searched for relevant abstracts and posters: the Lysosomal Diseases Gordon Research Conference (GRC), the Society for the Study of Inborn Errors of Metabolism (SSIEM) annual symposium, the We’re Organizing Research on Lysosomal Diseases (WORLD) symposium, the International Congress of Inborn Errors of Metabolism (ICIEM), and the International Society for Pharmacoeconomics and Outcomes Research (ISPOR) annual conference.

### 2.2. Data Collection, Extraction, and Bias Assessment

Relevant abstracts and titles were identified by a single reviewer based on the criteria outlined in Supplementary Table S2. Articles meeting the inclusion criteria, or for which eligibility was unclear, were revisited for a full-text review by another single reviewer to confirm eligibility. Systematic reviews were included for full-text review to identify potentially relevant studies in their reference lists. Remaining uncertainties regarding eligibility were addressed by a separate reviewer. Relevant data from eligible publications were extracted to populate predefined summary tables, with all data being checked by a second reviewer. Extracted data included the study design, country, number of patients, baseline demographics, length of follow-up, treatment details, outcomes of interest (see below), funding information, and author conclusions. Publications were classified and summarized using the following categories: agalsidase alfa single-arm studies, agalsidase beta single-arm studies, mixed or non-specified ERT or other treatment single-arm studies, comparator studies, and studies examining the effects of switching between treatments (switch studies). For each included study, a risk of bias assessment was performed using the RoB2 tool for randomized trials, the Risk Of Bias In Non-randomized Studies—of Interventions (ROBINS-I) tool for non-randomized studies, or the Joanna Biggs Institute (JBI) critical appraisal checklist for systematic reviews and research synthesis (for meta-analyses).

### 2.3. Outcome Measures

The SLR strategy aimed to identify literature relating to the clinical efficacy and real-world effectiveness of different treatments for FD. Outcomes of interest were renal outcomes (glomerular filtration rate [GFR] and proteinuria); cardiovascular outcomes (left ventricular mass index [LVMI]/left ventricular mass [LVM] and other cardiac structure outcomes); cerebrovascular outcomes (white matter lesions [WMLs], stroke, transient ischemic attack [TIA], and other cerebrovascular outcomes); and the Mainz Severity Score Index (MSSI), a scoring system developed to measure the severity of general, neurological, cardiovascular, and renal signs and symptoms of FD. The SLR also identified safety outcomes data (adverse events and serious adverse events); these have not been included here owing to the heterogeneous nature of safety reporting across the included clinical studies.

## 3. Results

**Figure 1:**
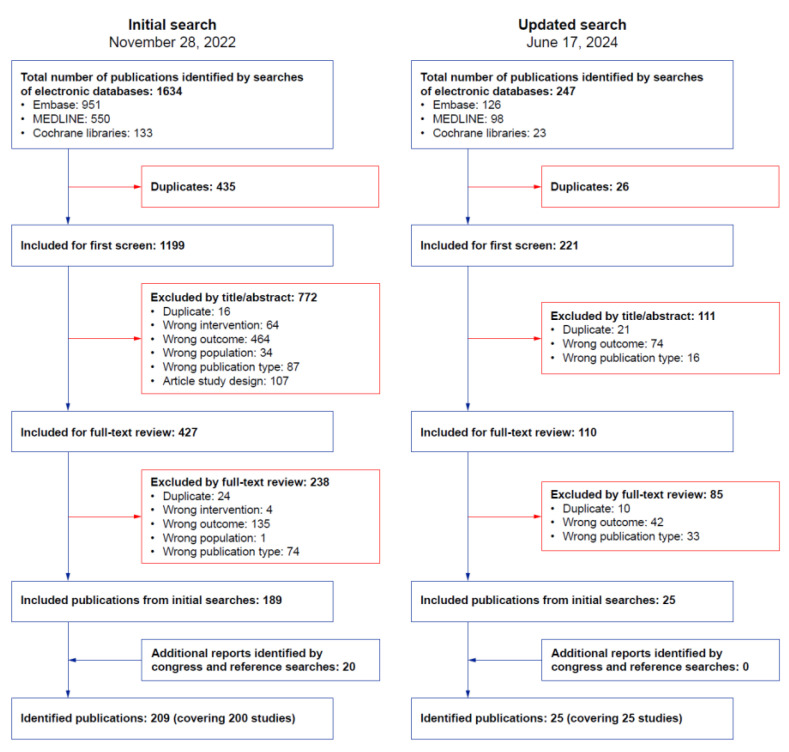
PRISMA flow diagram for identified publications (initial and updated searches). PRISMA, Preferred Reporting Items for Systematic reviews and Meta-Analyses.

A flow diagram summarizing the search and screening process is presented in Figure 1. Overall, 1881 articles were identified from the electronic databases. An additional 20 relevant publications were identified in congress searches. After removing duplicates and non-eligible studies, 234 publications remained, covering a total of 225 studies. Most studies were real-world, observational studies (*n* = 150; 67%); the remaining studies were clinical trials (*n* = 71; 32%) and meta-analyses (*n* = 2; 1%). Geographically, study locations were wide-ranging, with the largest number conducted in Germany (*n* = 25) or across multiple countries (*n* = 70; Supplementary Figure S1). Most studies included only adults (*n* = 172; 74%). In studies that reported clinical outcomes, the number of included patients ranged from six [18,19] to 2051 [20]. Seven studies included over 500 patients [20,21,22,23,24,25,26]. The pharmaceutical industry funded 122 (54%) studies (41 studies were not industry funded; funding was not reported in the remaining 62 studies identified).

Overall, 187 studies included ERT with agalsidase alfa and/or agalsidase beta, 30 studies included treatment with migalastat, and 11 studies included second-generation ERT with pegunigalsidase alfa. Of the publications on single-arm studies (i.e., those without a comparator arm), 37 investigated the effects of agalsidase alfa, and 25 examined the effects of agalsidase beta. In addition, 23 publications examined mixed or non-specified ERT and 21 examined other treatments (including second-generation ERT) in single-arm studies. Overall, 32 publications compared various treatment regimens with each other or with untreated or placebo-treated patients. Only three studies compared the effects of agalsidase alfa and agalsidase beta [27,28,29]. In total, 20 publications examined the effects of switching between different treatments; this excluded investigations in which the effects of the switch were not reported as outcomes [30,31,32,33,34,35,36,37]. A summary of the risk of bias assessments is shown in Supplementary Table S3. Studies reported in congress publications were more likely to have a high risk of bias or lack information than studies reported in journal articles.

### 3.1. Renal Outcomes

FD causes glycosphingolipid accumulation and damage throughout the glomerular, vascular, and interstitial compartments of the kidney, resulting in progressive decline in kidney function and increased proteinuria [38]. Publications reporting GFR outcomes (reported as GFR, estimated GFR [eGFR], or measured GFR) presented data from single-arm studies of agalsidase alfa (*n* = 29) [26,39,40,41,42,43,44,45,46,47,48,49,50,51,52,53,54,55,56,57,58,59,60,61,62,63,64,65,66], agalsidase beta (*n* = 19) [23,30,67,68,69,70,71,72,73,74,75,76,77,78,79,80,81,82,83], mixed or non-specified ERT (*n* = 13) [31,35,36,84,85,86,87,88,89,90,91,92,93], other treatments (*n* = 18) [94,95,96,97,98,99,100,101,102,103,104,105,106,107,108,109,110,111], comparator studies (*n* = 21) [27,28,29,33,108,112,113,114,115,116,117,118,119,120,121,122,123,124,125,126,127], and switch studies (*n* = 13) [128,129,130,131,132,133,134,135,136,137,138,139,140]. An overview of the GFR outcome data from agalsidase alfa and agalsidase beta single-arm studies and the comparator studies is presented in Table 1. Key results from the other types of studies are provided in Supplementary Table S4.

Treatment duration in the identified agalsidase alfa single-arm studies ranged from 26 weeks [51] to 20 years [40] (Table 1A). In 16 studies with follow-up durations of less than 5 years, treatment with agalsidase alfa demonstrated a renoprotective effect, stabilizing renal function (i.e., no statistically significant change in eGFR over the follow-up period) [39,44,46,48,49,50,51,54,56,58,59,60,61,62,63,64,65,66]. In 10 longer-term studies (>5 years), mixed results were reported [26,40,42,43,45,47,52,53,55,57]. In a retrospective, observational study, eGFR values remained stable over 10 years of treatment [47]. In a 10-year, prospective clinical study, weekly infusions of agalsidase alfa at a dose of 0.2 mg/kg were found to be beneficial for patients whose kidney function had declined with standard every-other-week dosing [53,57]. However, this study included small numbers of patients (*n* = 12). In two larger Fabry Outcome Survey (FOS) studies (*n* > 150), with longer follow-up periods (median treatment duration ≥ 12 years), agalsidase alfa treatment stabilized eGFR in patients with preserved kidney function (eGFR ≥ 60 mL/min/1.73 m^2^) or low proteinuria (≤0.5 g/24 h) at baseline [42,43]. In contrast, patients with impaired renal function (eGFR < 60 mL/min/1.73 m^2^) or high proteinuria (>0.5 g/24 h) at baseline continued to show declines in eGFR during treatment, although these declines were less than expected based on FD natural history data [42,43]. In a large, retrospective cohort study (*n* = 560) with a mean treatment duration of 7.6 years, patients who initiated agalsidase alfa treatment after 18 and 30 years of age had significant annual declines in mean (95% confidence interval [CI]) eGFR over the follow-up period (−1.12 [−1.77, −0.47] and −2.60 [−3.16, −2.04] mL/min/1.73 m^2^, respectively), whereas those who initiated treatment in childhood (≤18 years old) did not have significant annual changes in mean eGFR (−0.44 [−0.42, 1.30] mL/min/1.73 m^2^) [26]. In the longest agalsidase alfa study reported (in a congress publication), which included 66 patients from the FOS registry who had received agalsidase alfa for at least 19 years, the mean (standard deviation [SD]) annual rate of eGFR decline was −2.04 (0.36) mL/min/1.73 m^2^ (*n* = 48) [40].

Treatment duration in single-arm studies of agalsidase beta reporting GFR outcomes ranged from 22.7 months [68] to 10 years [82] (Table 1A). In total, 17of 19 identified studies reported a stabilizing effect of agalsidase beta on kidney function [23,30,67,68,69,70,71,72,73,74,75,76,77,78,79,80,81,82,83], which varied based on factors, such as baseline GFR (based on clearance of ^93^Technetium DTPA [68] and eGFR [72]) and level of renal involvement [23,70,82]. In a long-term analysis (median treatment duration: 10 years) of patients with classic FD who participated in a phase 3 clinical trial of agalsidase beta, its extension study, and the Fabry Registry (*n* = 52), patients with low renal involvement at baseline (urine protein-to-creatinine ratio [UPCR] ≤ 0.5 g/g and <50% sclerotic glomeruli on kidney biopsy) had a mean annual eGFR decline of −1.89 mL/min/1.73 m^2^ [82]. In contrast, those with high renal involvement (UPCR > 0.5 g/g or ≥50% sclerotic glomeruli on kidney biopsy) had a mean annual eGFR decline of −6.82 mL/min/1.73 m^2^ despite treatment [82]. In patients from the Fabry Registry who initiated agalsidase beta treatment at a young age (5–30 years old; median follow-up: ≥5 years), annual eGFR decline was modest for both males (−1.18 mL/min/1.73 m^2^; *n* = 117) and females (−0.92 mL/min/1.73 m^2^; *n* = 59) with low renal involvement (UPCR ≤ 0.5 g/g or urine albumin-to-creatinine ratio [UACR] ≤ 0.3 g/g) at baseline, whereas males with high renal involvement (UPCR > 0.5 g/g or UACR > 0.3 g/g) showed a greater annual decline in eGFR (−2.39 mL/min/1.73 m^2^; *n* = 23) [23].

Single-arm studies evaluating mixed or non-specified ERT also demonstrated the importance of early initiation of ERT for better long-term renal outcomes (Supplementary Table S4). In a retrospective cohort of adults receiving ERT (*n* = 293; median follow-up: 6.8 years), those with lower eGFR at baseline had an increased risk of having a first renal event (hazard ratio [per −10 mL/min/1.73 m^2^]: 5.57, *p* = 0.02) [31]. In single-arm studies of other treatments, 12 studies (13 publications) specifically evaluated the effects of migalastat [14,97,98,99,100,101,103,104,105,106,108,109,110], four examined second-generation ERT with pegunigalsidase alfa [94,95,96,107], and one explored the effects of a lentivirus-mediated gene therapy [102] on eGFR in patients with FD. Renal function remained stable in most migalastat studies (follow-up periods ranged from 6 months to 7 years) [98,99,100,101,105,106,108,110,111]. In a prospective, observational study of migalastat treatment over 24 months (*n* = 59), mean (SD) eGFR significantly decreased from 98.6 (15.2) to 90.4 (14.7) mL/min/1.73 m^2^ (*p* = 0.0317) in females and from 99.8 (22.6) to 92.4 (24.0) mL/min/1.73 m^2^ (*p* = 0.0028) in males [103,104]. In the four studies that examined the effects of pegunigalsidase alfa (*n* = 15–30), eGFR remained stable over follow-up periods of 3 to 72 months [94,95,96,107]. In the single study of lentivirus-mediated gene therapy, five patients were infused with autologous lentivirus-transduced, CD34+-selected, hematopoietic stem/progenitor cells engineered to express α-Gal A [102]. Patients had a transient increase in eGFR during the active treatment phase, although eGFR returned to baseline levels and remained stable after gene therapy (follow up: 12–33 months), except for one patient who displayed progressive chronic kidney disease with significant proteinuria at baseline [102].

In total, 12 studies reporting on GFR outcomes compared active treatments with no treatment or placebo [33,108,112,114,115,116,117,120,121,122,123,124,126], 10 studies directly compared different active treatments [27,28,29,108,113,114,115,119,122,125,127] (two studies included active comparator groups and untreated groups [112,114]), and two compared different doses of the same treatment [33,118] (Table 1B). Overall, studies that included patients who received no treatment or placebo indicated that approved treatments for FD generally resulted in stabilization of GFR outcomes, although study designs, patient populations, and follow-up times varied considerably. In a comparison of FOS registry data (*n* = 740; median treatment duration: 5.4 years) and published findings for untreated patients with FD, treatment with agalsidase alfa slowed eGFR decline in male patients with low eGFR (<60 mL/min/1.73 m^2^) at baseline [112]. A stabilizing effect of ERT on eGFR was also observed in patients with FD following kidney transplantation [113,114,115]. Three studies (two cohort studies and one randomized controlled open-label trial) directly compared agalsidase alfa and agalsidase beta with respect to effects on eGFR; no significant differences between the two treatments were identified [27,28,29]. In the largest of these studies, there was no significant difference in the slope of eGFR between patients treated with agalsidase alfa (*n* = 248) or agalsidase beta (*n* = 139) over a median follow-up period of 4.9 years. This finding was seen regardless of baseline eGFR (≥60 or <60 mL/min/1.73 m^2^) and after adjusting for additional factors including sex, phenotype, and baseline proteinuria [29]. In the phase 3 BALANCE trial, pegunigalsidase alfa showed noninferiority to agalsidase beta based on the rate of eGFR decline over 24 months in adults with deteriorating renal function at baseline (linear eGFR slope more negative than −2 mL/min/1.73 m^2^/year; *n* = 52) [127].

Ten studies (17 publications) assessed the effects of switching from one FD treatment to another on GFR outcomes (Supplementary Table S4), of which six studies (seven publications) focused on patients switching from agalsidase beta to agalsidase alfa following an agalsidase beta supply shortage from June 2009 to January 2012 [128,131,132,133,136,137,138]. Four studies showed mild deterioration in eGFR after switching from the standard dosing of agalsidase beta to agalsidase alfa (*n* = 22–38) [131,132,133,138]. However, all of these studies were observational and nonrandomized; therefore, treatment groups were not homogeneous. Two other studies demonstrated maintenance of eGFR in patients following the switch from agalsidase beta to agalsidase alfa (*n* = 71 and 11) [128,136,137]. In the phase 3 BRIDGE trial (*n* = 20), pegunigalsidase alfa slowed progression of kidney disease (mean eGFR slope was −5.90 mL/min/1.73 m^2^/year with agalsidase alfa compared with −1.19 mL/min/1.73 m^2^/year 12 months after switching to pegunigalsidase alfa) [139]. The small number of patients means the data could be influenced by a few patients. There was no difference in GFR outcomes in studies in which patients were switched from ERT to migalastat [129,130,134,135,140].

Identified studies reporting on proteinuria in patients receiving FD treatments (42 studies in 44 publications) are shown in Supplementary Table S5. Overall, the majority of studies showed little to no effect of treatment on proteinuria in patients with FD, although improvements in at least one proteinuria outcome were reported in four out of the six studies in children [33,51,76,81]. However, supportive treatment with an angiotensin-converting enzyme inhibitor (ACEi) or angiotensin receptor blocker (ARB) is standard practice for any patient with proteinuric kidney disease, and has been shown to provide sustained reductions in proteinuria and stabilization of kidney function in a small study of patients with severe FD [79]. The differing use of supportive treatments with ACEi and ARB may have affected the results from other studies and cannot be independently assessed. The additional effect of sodium-glucose co-transporter-2 inhibitors combined with ERT on proteinuria and renal outcomes in FD remains to be established.

**Table 1:** Overview of GFR data from (**A**) single-arm agalsidase alfa or agalsidase beta studies and (**B**) comparator studies.

(A)			
Author Year Study Identifier	*N*	Treatment Duration	Key Results
Agalsidase alfa single-arm studies			
Beck 2004 [39] FOS	150	Mean [max]: 17 [56] months	Observational study using data from FOS In patients with a baseline GFR between 60 and 90 mL/min/1.73 m^2^ (*n* = 104), GFR was stable following 1 year (*n* = 57) and 2 years (*n* = 30) of treatment Patients with a baseline GFR between 30 and 60 mL/min/1.73 m^2^ (*n* = 46) also had stable GFR at 1 year (*n* = 26) and 2 years (*n* = 18) of treatment
Cybulla 2022 [43] FOS	193	Mean/median (SD) [range]: 12.6/11.9 (5.0) [5.0, 21.6] years	Observational study using male patient data from FOS eGFR (mL/min/1.73 m^2^) ◦ Low proteinuria (*n* = 114): intercept (SE), 9.67 (4.00); slope (SE), −1.61 (0.28); *p* < 0.0001 ◦ High proteinuria (*n* = 51): intercept (SE), 4.15 (3.69); slope (SE), −3.62 (0.42); *p* < 0.0001 ◦ *p* (high vs. low proteinuria) < 0.0001
Feriozzi 2009 [44] FOS	165	3 years	Observational study using data from FOS eGFR remained stable in men with stage III disease; in those with stage I or II renal disease, there was a small but significant decrease in eGFR (*p* < 0.01) The mean decrease in eGFR in all male patients (*n* = 115) was 2.66 ± 5.07 mL/min/1.73 m^2^/year The decline in eGFR (SD) was numerically larger in patients with urine protein levels > 500 mg/24 h (*n* = 14) −3.98 (7.86) mL/min/1.73^2^/year than in patients with urine protein levels <500 mg/24 h (*n* = 40) −1.68 (3.59) mL/min/1.73^2^/year
Feriozzi 2012 [45] FOS	208	Mean [range]: 7.4 [5.0, 11.2] years	Observational study using data from FOS Mean (95% CI) yearly change in eGFR was −2.2 (−2.8, −1.7) mL/min/1.73 m^2^ in men (*n* = 134) and −0.7 (−1.4, 0.0) mL/min/1.73 m^2^ in women (*n* = 74)
Giugliani 2023 * [40] FOS	66	Median [IQR]: 20.03 [19.55, 20.64] years	Observational study using data from FOS Baseline eGFR: 99.35 (27.07) mL/min/1.73 m^2^ Annual rate of change: mean (SE), −2.04 (0.36) mL/min/1.73 m^2^ (*n* = 48) Mean reduction: males (*n* = 38), −2.27 mL/min/1.73 m^2^; females (*n* = 10), −1.24 mL/min/1.73 m^2^ Statistical significance values not reported
Goker-Alpan 2016 [46]	14	Median [range]: 54.5 [54.0, 59.0] weeks	An open-label trial of agalsidase alfa in children Mean (95% CI) change from baseline at week 55 of 0.15 (−11.39, 11.70) mL/min/1.73 m^2^ (median, 0.72 [−27.5, 35.9] mL/min/1.73 m^2^)
Hughes 2011 [48] FOS	250	≥4 years	Observational study using data from FOS Median [10–90th percentile] eGFR (MDRD) at baseline (mL/min/1.73 m^2^): women, 71.8 [56.5–87.6]; men, 88.2 [41.1–137.0] Median [10–90th percentile] eGFR (MDRD) at 4 years (mL/min/1.73 m^2^): women (*n* = 41), 69.6 [44.8–91.1], *p* = 0.007 vs. baseline; men (*n* = 105), 80.3 [34.2–119.7], *p* < 0.001 vs. baseline
Kampmann 2015 [47]	45	Median [range]: 10.8 [9.6, 12.5] years	Single-center observational study in Germany Among patients (8 males, 5 females) with pretreatment eGFR ≥ 90 mL/min/1.73 m^2^, the mean annual decline in eGFR over 10 years was not statistically significant In patients with poorer renal function (10 males, 11 females), eGFR seemed to improve in the first 3 years of ERT After 10 years, however, eGFR values were not significantly changed regardless of renal function before ERT
Kleinert 2006 [64] FOS	60	>2 years	Observational study using data from FOS Median [10th–90th percentile] eGFR (mL/min/1.73 m^2^): baseline: 88 [49–126] (*n* = 54); 1 year: 86; 2 years: 84
Mehta 2009 [65] FOS	181	Mean (SD): 3.1 (2.1) years	Observational study using data from FOS All patients: mean (SD) eGFR (mL/min/1.73 m^2^) ◦ Year 1 (*n* = 121); baseline: 85.46 (29.21); year-end: 82.31 (29.12); change: −3.15 (13.01); annualized: −3.15; *p* = 0.01 ◦ Year 2 (*n* = 143); baseline: 86.17 (27.65); year-end: 79.03 (29.47); change: −7.14 (16.82); annualized: −3.57; *p* = 0.001 ◦ Year 3 (*n* = 137); baseline: 87.51 (28.63); year-end: 76.77 (27.01); change: −10.74 (14.91); annualized: −3.58; *p* = 0.001 ◦ Year 5 (*n* = 150); baseline: 87.04 (28.80); year-end: 74.75 (28.65); change: −12.29 (18.40); annualized: −2.46; *p* = 0.001 Men: mean (SD) eGFR (mL/min/1.73 m^2^) ◦ Year 1 (*n* = 80); baseline: 94.02 (30.56); year-end: 90.30 (31.02); change: −3.72 (14.84); annualized: −3.72; *p* = 0.028 ◦ Year 2 (*n* = 99); baseline: 93.23 (28.37); year-end: 85.58 (31.60); change: −7.65 (19.05); annualized: −3.83; *p* = 0.0001 ◦ Year 3 (*n* = 92); baseline: 96.26 (29.01); year-end: 82.65 (27.91); change: −13.61 (14.66); annualized: −4.54; *p* < 0.0001 ◦ Year 5 (*n* = 103); baseline: 94.71 (29.77); year-end: 78.84 (31.08); change: −15.87 (20.04); annualized: −3.17; *p* < 0.0001 Women: mean (SD) eGFR (mL/min/1.73 m^2^) ◦ Year 1 (*n* = 41); baseline: 68.76 (16.81); year-end: 66.71 (16.33); change: −2.05 (8.45); annualized: −2.05; *p* = NS ◦ Year 2 (*n* = 44); baseline: 70.28 (17.81); year-end: 64.30 (16.46); change: −5.98 (10.27); annualized: −2.99; *p* = 0.0004 ◦ Year 3 (*n* = 45); baseline: 69.61 (17.49); year-end: 64.74 (20.59); change: −4.87 (13.80); annualized: −1.62; *p* = 0.02 ◦ Year 5 (*n* = 47); baseline: 70.23 (17.37); year-end: 65.80 (19.96); change: −4.44 (10.72); annualized: −0.89; *p* = 0.007 Hyperfiltration (≥130 mL/min/1.73 m^2^): mean (SD) eGFR (mL/min/1.73 m^2^) ◦ Year 1 (*n* = 11); baseline: 144.49 (13.40); year-end: 131.13 (21.61); change: −13.35; annualized: −13.35; *p* = 0.05 ◦ Year 2 (*n* = 12); baseline: 139.64 (8.75); year-end: 118.04 (43.46); change: −21.60; annualized: −10.80; *p* = NS ◦ Year 3 (*n* = 13); baseline: 142.47 (13.19); year-end: 114.36 (16.56); change: −28.11; annualized: −9.37; *p* = 0.001 ◦ Year 5 (*n* = 14); baseline: 143.50 (13.25); year-end: 108.07 (20.26); change: −35.43; annualized: −7.09; *p* = 0.001 CKD Stage 1 (90–129 mL/min/1.73 m^2^): mean (SD) eGFR (mL/min/1.73 m^2^) ◦ Year 1 (*n* = 35); baseline: 107.06 (11.15); year-end: 103.94 (18.37); change: −3.12; annualized: −3.12; *p* = NS ◦ Year 2 (*n* = 47); baseline: 106.89 (10.34); year-end: 99.06 (19.38); change: −7.83; annualized: −3.91; *p* = 0.01 ◦ Year 3 (*n* = 45); baseline: 107.63 (10.34); year-end: 95.37 (19.49); change: −12.26; annualized: −4.09; *p* = 0.001 ◦ Year 5 (*n* = 48); baseline: 107.06 (10.30); year-end: 93.93 (23.48); change: −13.13; annualized: −2.63; *p* = 0.001 CKD Stage 2 (60–89 mL/min/1.73 m^2^): mean (SD) eGFR (mL/min/1.73 m^2^) ◦ Year 1 (*n* = 55); baseline: 73.61 (8.59); year-end: 72.21 (12.32); change: −1.39; annualized: −1.39; *p* = NS ◦ Year 2 (*n* = 60); baseline: 74.16 (8.73); year-end: 69.33 (14.03); change: −4.83; annualized: −2.41; *p* = 0.001 ◦ Year 3 (*n* = 58); baseline: 73.25 (8.54); year-end: 66.84 (13.71); change: −6.40; annualized: −2.13; *p* = 0.001 ◦ Year 5 (*n* = 64); baseline: 73.98 (8.74); year-end: 66.33 (17.26); change: −7.64; annualized: −1.53; *p* = 0.001 CKD Stage 3 (30–59 mL/min/1.73 m^2^): mean (SD) eGFR (mL/min/1.73 m^2^) ◦ Year 1 (*n* = 19); baseline: 48.78 (8.23); year-end: 45.92 (9.88); change: −2.87; annualized: −2.87; *p* = 0.05 ◦ Year 2 (*n* = 23); baseline: 49.76 (7.93); year-end: 45.23 (9.82); change: −4.54; annualized: −2.27; *p* = 0.01 ◦ Year 3 (*n* = 21); baseline: 49.77 (8.31); year-end: 41.07 (10.19); change: −8.70; annualized: −2.90; *p* = 0.001 ◦ Year 5 (*n* = 23); baseline: 49.76 (7.93); year-end: 39.53 (14.50); change: −10.23; annualized: −2.05; *p* = 0.001 CKD Stage 4 (15–29 mL/min/1.73 m^2^): mean (SD) eGFR (mL/min/1.73 m^2^) ◦ Year 1 (*n* = 1); baseline: 28.78; year-end: 34.60; change: +5.81; annualized: +5.81; *p* = NA ◦ Year 2 (*n* = 1); baseline: 28.78; year-end: 29.29; change: +0.50; annualized: +0.25; *p* = NA ◦ Year 3 (*n* = 0); data: NR ◦ Year 5 (*n* = 1); baseline: 28.78; year-end: 37.05; change: +8.27; annualized: +1.65; *p* = NA
Parini 2020 [26] FOS	560	Mean (SD): 7.6 (4.8) years	Observational study using data from FOS Overall mean eGFR (mL/min/1.73 m^2^): cohort 1: ≤18 years, 117.9; cohort 2: 18–30 years, 114.1; cohort 3: >30 years, 79.9 Changes during follow-up ◦ Cohort 1: ≤ 18 years; no significant annual changes in eGFR slope (95% CI) −0.44 mL/min/1.73 m^2^ (−0.42, 1.30) proteinuria was observed throughout the follow-up period ◦ Cohort 2: 18–30 years; although proteinuria remained stable, eGFR showed a significant annual decline over the follow-up period: eGFR slope (95% CI), −1.12 mL/min/1.73 m^2^ (−1.77, −0.47; *p* < 0.001) ◦ Cohort 3: >30 years; a significant annual deterioration over the follow-up period was shown for eGFR (slope (95% CI), −2.60 mL/min/1.73 m^2^ (−3.16, −2.04; *p* < 0.001)
Pastores 2007 [49]	22	Median [range]: 42 [11, 65] weeks	A phase 2, multi-center, open-label study Patients with FD and ESRD undergoing dialysis or who had a history of kidney transplantations were treated with agalsidase alfa After 26 weeks of agalsidase alfa therapy, mean (SEM) eGFR increased slightly to 71.6 (8.0) mL/min/1.73 m^2^ (*n* = 10) from 64.5 (6.0) mL/min/1.73 m^2^ (*n* = 12), which approached statistical significance in this relatively small cohort of patients (*p* = 0.07)
Pintos-Morell 2023 [41] FOS	285	Mean (SD): Early initiators group: 11.2 (5.7) years; Late initiators group: 11.8 (5.8) years	Observational study using data from FOS Rates of annual eGFR change; mean (SE) ◦ Early-initiators (<25 years at treatment start) group (*n* = 40): −1.45 (0.52) mL/min/1.73 m^2^/year; *p* = 0.0054 ◦ Late-initiators (≥25 years at treatment start) group (*n* = 103): −2.84 (0.32) mL/min/1.73 m^2^/year; *p* < 0.0001 The rate of eGFR change was significantly slower in the early initiators compared with the late initiators group; *p* = 0.0234
Ramaswami 2011 [50] FOS	8	Mean (SD): 4.2 (1.9) years	Observational study in children using data from FOS Study of patients in the FOS registry who received their first dose of agalsidase alfa before reaching the age of 7 years Mean (SD) baseline eGFR (mL/min/1.73 m^2^): 124.4 (25.7); final eGFR, 112.7 (21.79)
Ramaswami 2012 [66] FOS	98	≥6 months	Observational study in pediatric patients (<18 years old) using data from FOS Mean (SD) eGFR at baseline and 12 months (*n* = 40): 96.8 (26.5) and 94.2 (21.2) mL/min/1.73 m^2^ Mean (SD) eGFR at baseline and 24 months (*n* = 45): 93.2 (26.1) and 92.5 (25.2) mL/min/1.73 m^2^
Ramaswami 2019 [42] FOS	152	Median [range]: Evaluable treated renal cohort (*n* = 152) 13.3 [10.1, 18.4] years; Females (*n* = 62) 12.5 [10.1, 17.1] years; Males (*n* = 90): 14.4 [10.1, 18.4] years	Observational study in children using data from FOS Pediatric patients who had received agalsidase alfa ERT over a 10-year period Mean (SD) baseline eGFR (mL/min/1.73 m^2^): evaluable treated renal cohort, 89.04 (26.49) Number of patients (%) in eGFR category <60 mL/min/1.73 m^2^: evaluable treated renal cohort, 21/152 (13.8%) Mean [95% CI] eGFR at year 10 (mL/min/1.73 m^2^): females 79.1 [74.1, 84.0]; males 77.5 [70.1, 84.9] Mean [95% CI] eGFR slope in patients in eGFR category ≥ 60 mL/min/1.73 m^2^ at baseline: females (*n* = 52), −0.55 [−1.12, 0.01]; males (*n* = 79), −1.99 [−2.45, −1.54] Mean [95% CI] eGFR slope in patients in eGFR category <60 mL/min/1.73 m^2^ at baseline: females (*n* = 10), −0.14 [−1.43, 1.15]; males (*n* = 11), −2.79 [−4.01, −1.56]
Ries 2006 [51]	24	26 weeks	Open-label study in pediatric patients Study in pediatric patients with FD (between 6.5 and 18 years of age) naive to ERT Mean (SEM) baseline eGFR was 121 (5.0) mL/min/1.73 m^2^ 7 patients had eGFR > 135 mL/1.73 m^2^ 2 patients showed evidence of kidney dysfunction defined as eGFR between 60 and 89 mL/min/1.73 m^2^ Mean (SEM) eGFR did not change significantly during the treatment (mL/min/1.73 m^2^): 9 weeks, 119.2 (3.6); 17 weeks, 120.0 (3.9); and 26 weeks, 116.0 (3.9) The eGFR of patients with possible hyperfiltration (eGFR > 135 mL/min/1.73 m^2^) returned to the reference range
Sasa 2019 [52]	493	Mean [range]: 3.5 [0.0, 7.9] years; <0.5: (*n* = 35); >0.5–1: (*n* = 31); >1–2: (*n* = 48); >2–3: (*n* = 69); >3–4: (*n* = 86); >4–5: (*n* = 132); >5–6: (*n* = 55); >6–7: (*n* = 20); >7–8: (*n* = 17)	A post-marketing surveillance study in Japan Mean (SD) yearly change in eGFR (mL/min/1.73 m^2^) ◦ Males (classic phenotype): year 0.5, 1.00 (*p* = 0.619); year 1, −1.03 (*p* = 0.289); year 2, −1.79 (*p* = 0.002); year 3, −2.09 (*p* = 0.001); year 4, −2.04 (*p* = 0.000); year 5, −2.50 (*p* = 0.000); year 6, −2.88 (*p* = 0.124); year 7, −0.44 (*p* = 0.814) ◦ Males (non-typical variant): year 0.5, −1.17 (*p* = 0.718); year 1, −1.24 (*p* = 0.544); year 2, −1.73 (*p* = 0.103); year 3, −2.04 (*p* = 0.028); year 4, −1.47 (*p* = 0.057); year 5, −0.95 (*p* = 0.447); year 6, −1.54 (*p* = 0.084); year 7, NR ◦ Females: year 0.5, −1.81 (*p* = 0.328); year 1, −1.61 (*p* = 0.059); year 2, −1.44 (*p* = 0.008); year 3, −1.02 (*p* = 0.020); year 4, −1.10 (*p* = 0.028); year 5, −1.89 (*p* = 0.009); year 6, −1.95 (*p* = 0.021); year 7, −2.64 (*p* = 0.078)
Schiffmann 2006 [54]	25	≤54 months	Open-label extension study for male patients Assessment of long-term renal effects and practicality of home infusions of agalsidase alfa Data reported for 24/25 patients: 12 had stage I renal disease (eGFR, ≥ 90 mL/min/1.73 m^2^) at baseline, 8 had stage II (eGFR, 60–89 mL/min/1.73 m^2^), and 4 had stage III (GFR, 30–59 mL/min/1.73 m^2^) renal disease Overall population: after 48 months, eGFR declined to 75.1 (32.7) mL/min/1.73 m^2^, *p* = 0.039 vs. baseline, mostly owing to changes in patients with stage 3 CKD
Schiffmann 2007 [53], Schiffmann 2015 [57]	12	≤10 years	Open-label single-center study in the US Long-term prospective clinical trial assessing the effects of EOW versus weekly agalsidase alfa infusions in patients who experienced ongoing decline in renal function of EOW dosing Before therapy with agalsidase alfa, mean (SD) eGFR was 77.8 (30.4) mL/min/1.73 m^2^ During EOW dosing, the mean (SD) rate of change of eGFR was −8.0 (2.8) mL/min/1.73 m^2^/year (−0.67 (0.23) mL/min/1.73 m^2^/month) During 2 years of weekly dosing (following EOW dosing), the mean rate of change of eGFR slowed to −3.3 ± 4.7 mL/min/1.73 m^2^/year (−0.27 ± 0.40 mL/min/1.73 m^2^/month); *p* = 0.01 After 10 years of follow-up, mean (SD) slope on agalsidase alfa every 2 weeks was −7.92 (2.88) and 3.84 (4.08) mL/min/1.73 m^2^/year on weekly enzyme infusions (*p* = 0.01)
Schiffmann 2013 [56]	73	12 months	Data pooled from three randomized, double-blind, placebo-controlled trials and their open-label extension studies 25 patients had stage II/III renal disease (baseline eGFR 30–90 mL/min/1.73 m^2^ Mean (SD) eGFR at baseline was 92.6 (32.4) mL/min/1.73 m^2^ and remained stable at 92.1 (32.4) at 12 months
Schiffmann 2014 [55]	17	Mean (SD): 6.5 (0.6) years	Open-label multi-center extension study for pediatric patients Evaluating safety and efficacy of long-term agalsidase alfa ERT in children with FD Pediatric patients with FD (7–17 years of age at study enrollment) received agalsidase alfa Mean (SD) baseline eGFR was 123.29 (16.00) mL/min/1.73 m^2^ Most individuals showed relatively stable eGFR over time
Schwarting 2006 [58] FOS	20	2 years	Observational study using data from FOS eGFR (mL/min/1.73 m^2^), median [10th–90th percentile] ◦ Baseline: CKD stage 2 (*n* = 12), 70 [63–83]; CKD stage 3 (*n* = 8), 47 [35–55] Renal function in 12 patients (10 females and 2 males; mean age: 40 years; range: 19, 71 years) with baseline CKD stage 2 declined significantly (*p* < 0.05) in the year before treatment began Renal function in 8 patients (4 females and 4 males; mean age: 49 years; range: 29, 69 years) with CKD Stage 3 also declined in the year before the start of treatment, but this change was not significant After 1 year of treatment, however, the progressive loss of GFR had been stabilized in both groups of patients Renal function remained stable in 8 patients with CKD stage 2 and in 5 patients with CKD stage 3 studied for 2 years after the start of ERT
Schwarting 2006b [59] FOS	401	>2 years (*n* = 219) >3 years (*n* = 133)	Observational study using data from FOS Mean, median [range] eGFR (mL/min/1.73 m^2^) ◦ Patients given 1 year of therapy with agalsidase alfa - Female (*n* = 52): baseline: 66.8, 66.7 [32.0, 89.5]; year 1: 65.2, 64.5 [29.3, 87.4] - Male (*n* = 47): baseline: 66.0, 68.3 [38.4, 89.5]; year 1: 63.2, 61.7 [26.1, 113.0] ◦ Patients given 2 years of therapy with agalsidase alfa - Female (*n* = 34): baseline: 65.6, 64.3 [32.0, 89.5]; year 2: 63.1, 63.5 [30.0, 97.7] - Male (*n* = 28): baseline: 69.4, 73.1 [39.4, 89.5]; year 2: 63.3, 63.0 [12.8, 92.4] ◦ Patients given 3 years of therapy with agalsidase alfa - Female (*n* = 13): baseline: 68.4, 64.2 [47.6, 89.5]; year 3: 66.3, 65.6 [48.9, 88.2] - Male (*n* = 12): baseline: 65.6, 69.3 [38.4, 84.5]; year 3: 60.6, 62.4 [23.9, 91.2]
Thofehrn 2009 [60]	9	36 months (*n* = 6) 18 months (*n* = 2) 12 months (*n* = 1)	Single-center observational study in Brazil Mean (SD) [range] GFR (mL/min/1.73 m^2^) ◦ Baseline (*n* = 8): 110.30 (28.68) (1 patient progressed to ESRD and underwent transplant, excluded from analysis of renal function) ◦ 12 months (*n* = 8): 114.78 (33.92) ◦ 24 months (*n* = 5): 104.38 (35.66) ◦ 36 months (*n* = 5): 122.61 (46.85) No significant (*p* > 0.05) differences were found
Tsuboi 2017 [61] Japan Fabry Research—002	36	Median [range]: 62.5 [8, 84] months	Prospective observational study in Japan Cohort included ERT treatment-naive patients and those who were receiving agalsidase alfa 0.2 mg/kg EOW Mean (SD) baseline eGFR: 83.5 (28.6); after follow-up: 82.6 (29.4) mL/min/1.73 m^2^
West 2009 [62]	108	Mean, median (SD): 2.0, 1.6 (1.0) years Mean (SD) treatment years by baseline GFR range (mL/min/1.73 m^2^) ≥135 (*n* = 8): 1.2 (0.2); 90 to <135 (*n* = 33): 2.0 (1.0); 60 to <90 (*n* = 36): 2.2 (1.2); 30 to <60 (*n* = 14): 2.1 (0.9) 15 to <30 (*n* = 2): 1.0	Summary of 3 prospective, randomized, placebo-controlled trials and their open-label extension studies Mean (SD) [median; range] GFR (mL/min/1.73 m^2^): ◦ Baseline before placebo: 88.9 (32.5) [92.3; 12.7, 160] ◦ After 6 months of placebo: 85.0 (37.6) [83.3; 12.5, 184] ◦ Mean (SD) annualized rate of change in GFR: −7.7 (38.0) mL/min/1.73 m^2^/year; *p* = 0.14 3 patients had renal hyperfiltration (i.e., baseline GFR > 135 mL/min/1.73m^2^) When these 3 patients were removed from the analysis of the placebo period: ◦ Mean (SD) GFR at baseline before placebo (mL/min/1.73 m^2^): 85.4 (29.6) ◦ Mean (SD) GFR after 6 months of placebo (mL/min/1.73 m^2^): 81.9 (34.8) ◦ Mean (SD) annualized rate of change in GFR −7.0 (32.9) mL/min/1.73 m^2^/year; *p* = 0.12 Mean (SD) [median; range] GFR at baseline before agalsidase alfa (mL/min/1.73 m^2^): 90.3 (31.2) [87.4; 25–184] Mean (SD) baseline GFR by baseline GFR range (mL/min/1.73 m^2^): ≥135 (*n* = 8), 151.9 (16.4); 90 to <135 (*n* = 33), 109.6 (13.4); 60 to <90 (*n* = 36), 78.1 (8.4); 30 to <60 (*n* = 14), 50.1 (8.2); 15 to <30 (*n* = 2), 26.7 Mean (SD) annualized rate of change in GFR for the entire study population: −4.8 (10.6) mL/min/1.73 m^2^/year (*p* = 0.0003 compared with no change); median, −4.1 mL/min/1.73 m^2^/year After removing 8 patients with hyperfiltration during agalsidase alfa therapy: rate of change in GFR, −2.9 (8.7) mL/min/1.73 m^2^/year (*p* = 0.0002 compared with no change)
Whybra 2009 [63]	36	4 years	Prospective single-center, open-label study of female patients in Germany A cohort of female patients with FD who were experiencing manifestations of FD and had not been previously treated with ERT Mean (SD) eGFR after 4 years of treatment: 91.0 (25.6) mL/min/1.73 m^2^ ◦ 4 patients with eGFR > 135 mL/min/1.73 m^2^ at baseline (defined as hyperfiltration): eGFR at after 4 years of treatment, 128.3 (4.6) mL/min/1.73 m^2^ Mean (SD) eGFR by baseline eGFR range (mL/min/1.73 m^2^; * *p* < 0.05 vs. baseline; ** *p* < 0.001 vs. baseline): ◦ eGFR > 135 (*n* = 4): month 12, 140.3 (26.8); month 24, 135.3 (18.3) *; month 36, 133.0 (17.0) *; month 48, 128.3 (4.6) ◦ eGFR 90–135 (*n* = 9): month 12, 98.7 (15.6); month 24, 98.5 (10.9); month 36, 106.7 (15.5); month 48, 100.4 (16.4) ◦ eGFR 60–89 (*n* = 20): month 12, 85.5 (13.2); month 24, 88.8 (14.5); month 36, 83.0 (17.9); month 48, 85.9 (20.2) ◦ eGFR 30–59 (*n* = 3): month 12, 50.4 (10.8); month 24, 55.8 (8.1); month 36, 49.7 (11.5); month 48, 47.3 (14.0) Responder definitions: I, less than 20% decrease in GFR from baseline; II, no shift to more severe CKD stage eGFR 90–135 mL/min/1.73 m^2^ at baseline (*n* = 9): responder definition I (%), 89; responder definition II (%), 89 ◦ eGFR 60–89 mL/min/1.73 m^2^ at baseline (*n* = 20): responder definition I (%), 95; responder definition II (%), 95 ◦ eGFR 30–59 mL/min/1.73 m^2^ at baseline (*n* = 3): responder definition I (%), 67; responder definition II (%), 100 Patients treated with ACEIs or ARBs during entire study (*n* = 7) ◦ Mean (SD): eGFR after 4 years of treatment, 65.0 (13.5) mL/min/1.73 m^2^; proteinuria after 4 years of treatment, 425 (531) mg/24 h Patients who began treatment with ACEIs or ARBs after starting treatment with agalsidase alfa (*n* = 6) ◦ Mean (SD): eGFR at last measurement before starting anti-angiotensin therapy, 87.7 (29.6) mL/min/1.73 m^2^; eGFR at study end, 83.0 (33.4) mL/min/1.73 m^2^
**Agalsidase beta single-arm studies**			
Benichou 2009 [67]	76	Median [range]: Phase 3: 55.9 [3.3, 60.7] months; Phase 4: 33.5 [1.9, 51.2] months	Retrospective analysis of efficacy data obtained from adult patients with FD who received agalsidase beta for up to 5 years during a phase 3 or phase 4 placebo-controlled trial and the associated open-label extension study No statistically significant correlation was found between eGFR slope and peak titer (the highest anti-aGAL IgG titer observed for a given patient during treatment) among male patients from either the phase 3 (r^2^ = −0.22, *n* = 56, *p* = 0.10) or phase 4 studies (r^2^ = −0.09, *n* = 66, *p* = 0.46) Similar correlation coefficients were found when both males and females were combined and when eGFR was examined as a function of titer/day
Breunig 2006 [68]	25	Mean (SD) [range]: 23 (8) [12, 37] months	A single-center observational study of German patients with different stages of FD Mean (SD) GFR (mL/min/1.73 m^2^) ◦ Patients with GFR > 90 (*n* = 16): baseline, 115 (18); follow-up, 102 (14); *p* = 0.15 ◦ Patients with GFR < 90 (*n* = 16): baseline, 71 (17) [*n* = 8]; follow-up, 60 (23); *p* = 0.039
Burlina 2019 * [70] The Fabry Registry	49	≥2.5 years	Observational study using data from the Fabry Registry Included patients started on agalsidase beta (1 mg/kg EOW) when aged 5–30 years eGFR outcomes were stratified by renal involvement (high and low) based on albuminuria/proteinuria levels eGFR decline was higher among 16 high renal involvement males than 66 LRI males (slopes, −2.56 vs. −1.29 mL/min/1.73 m^2^/year; *p* value of interaction <0.05; median age at first treatment 25.3 vs. 20.6 years) Slope estimates were unreliable among females
Cabrera 2017 [30]	39	Mean (SD): 68.2 (36.9) months	Long-term prospective and historical analysis of cohort of patients with FD from Argentina At baseline, 5 patients had eGFR < 60 mL/min/1.73 m^2^, 2 males; 3 females Mean (SD), eGFR (mL/min/1.73 m^2^): ◦ Males (*n* = 17): baseline: 96.9 (28); follow-up: 102 (29) ◦ Females (*n* = 13): baseline: 86 (23); follow-up: 84 (23)
Chen 2024 * [83]	22	48 weeks	Phase 4 open-label study of Chinese patients Baseline mean (SD) eGFR: 115.6 (37.3) mL/min/1.73 m^2^ Week 48 mean (SD) eGFR: 118.4 (42.9) mL/min/1.73 m^2^ Change: 0.0 (12.4) mL/min/1.73 m^2^
Dutra-Clarke 2021 [71]	20	2–20 years	Observational study in the US Annual decrease in eGFR of −1.88 mL/min/1.73 m^2^ (95% CI: −3.36, −0.40; *p* = 0.013) on ERT in males and females and −3.16 mL/min/1.73 m^2^ (95% CI: −4.97, −1.35; *p* = 0.001) for males alone
Germain 2015 [82]	52	Median [IQR]: 10 [7.3, 10.3] years	Analysis of phase 3 clinical trial data and Fabry Registry observational analysis LRI subgroup (*n*= 32): mean eGFR slope: −1.89 mL/min/1.73 m^2^/year *p* = 0.001 HRI subgroup (*n* = 20): mean eGFR slope: −6.82 mL/min/1.73 m^2^/year *p* < 0.001 Patients with LRI whose UPCR was ≤0.5 g/g throughout the treatment period: mean slope: −1.48 mL/min/1.73 m^2^/year *p* < 0.001 Patients with LRI whose UPCR rose above 0.5 g/g during the treatment period: mean slope: −2.6 mL/min/1.73 m^2^/year *p* < 0.001
Goicoechea 2020 * [72]	69	Median [range] follow-up: 60 [24, 120] months	An observational study of Spanish patients At the end of follow-up, eGFR remained stable in 42 males and 27 females Renal, neurological, or cardiac events were more frequent in patients with baseline eGFR ≤ 60 mL/min/1.73 m^2^ (log-rank, 12.423; *p* = 0.001), and this remained significant even after excluding incident renal events (log-rank, 4.086; *p* = 0.043) Lower baseline eGFR was associated with a 3- to 7-fold increase in the risk of clinical events in different Cox models
Hopkin 2023 [23] The Fabry Registry	785	Median: 6.3 years in males; 5.0 years in females	Observational study using data from the Fabry Registry Baseline eGFR: males: median 91.7 mL/min/1.73 m^2^; females: median 102.3 mL/min/1.73 m^2^; *p* < 0.001 Male patients (*n* = 524): subgroup with eGFR assessments for slope analysis and renal involvement classification (*n* = 140); LRI, 117 (83.6%); HRI, 23 (16.4%) eGFR slopes in males ◦ LRI subgroup: slope, −1.18 mL/min/1.73 m^2^/year; 95% CI: −1.66, −0.71; *p* < 0.001 ◦ HRI subgroup: slope, −2.39 mL/min/1.73 m^2^/year; 95% CI: −3.53, −1.25; *p* < 0.001 ◦ *p* difference: 0.055 ◦ Median follow-up: 6.3 years (LRI) vs. 5.6 years (HRI) Female patients (*n* = 261) ◦ Subgroup with LRI and eGFR assessments for slope analysis (*n* = 59); HRI subgroup analysis not possible ◦ eGFR slope: −0.92 mL/min/1.73 m^2^/year; 95% CI: −1.81, −0.04; *p* = 0.040 ◦ Median follow-up: 5.0 years Patients with baseline eGFR > 135 mL/min/1.73 m^2^ ◦ Male LRI (*n* = 7): eGFR slope, −2.95 mL/min/1.73 m^2^/year; 95% CI: −6.01, 0.12; *p* = 0.057 ◦ Female LRI (*n* = 6): eGFR slope, −3.20 mL/min/1.73 m^2^/year; 95% CI: −9.71, 3.32; *p* = 0.140 ◦ *p* difference: 0.893 ◦ Median follow-up: 4.9 years (males) vs. 5.8 years (females) ◦ Median baseline eGFR: 148.4 mL/min/1.73 m^2^ (males) vs. 138.0 mL/min/1.73 m^2^ (females) ◦ Median age at first treatment: 15.5 years (males) vs. 12.9 years (females) ◦ Median age at last eGFR assessment: 23.6 years (males) vs. 28.4 years (females)
Hwang 2022 [73]	10	22-week follow-up Mean (SD) [range]: 4.6 (3.2) [1, 9] years	A phase 2 open-label study in Korea 8 patients had received previous ERT with agalsidase beta, 2 patients were treatment naive Mean (SD) [range] eGFR: baseline: 107.00 (28.42) [68.00, 159.00] mL/min/1.73 m^2^; follow-up: 116.33 (29.77) [77.00–163.00] mL/min/m^2^
Lubanda 2009 [74]	21	96 weeks	Evaluation of a low dose, after a standard therapeutic dose, of agalsidase beta Patients received 12 infusions of agalsidase beta at 1.0 mg/kg EOW for 6 months (weeks 0–23), followed by up to 37 infusions at 0.3 mg/kg EOW for 18 months (weeks 24–96) ◦ Mean, median (SD) [range] eGFR (mL/min/1.73 m^2^): baseline: (*n* = 21), 93, 92.5 (21.0) [55–134] ◦ Median eGFR (mL/min/1.73 m^2^): 24 weeks, 93.1; 96 weeks, 92.8
Ortiz 2021 * [75]	254	Median post-treatment period: 4.1 years	Self-controlled comparison of renal function before and after ERT in male patients with LRI versus HRI LRI (UPCR ≤ 0.5 g/g or UACR ≤ 0.3 g/g) and HRI (ratios > 0.5 g/g or >0.3 g/g, respectively) ◦ Pre-treatment eGFR slope (mL/min/1.73 m^2^/year): whole cohort (N = 254): −2.22 (median pre-treatment period 1.1 years); LRI (n = 251): −1.73; HRI (N = 68): −2.93 ◦ eGFR slope, pre- vs. post-treatment (mL/min/1.73 m^2^/year): whole cohort (n = 254), −2.22 vs. −2.66 (p pre-post difference = 0.24; median pre-treatment period, 1.1 years; median post-treatment period, 4.1 years); LRI (n = 165), −1.73 vs. −1.92 (p pre-post difference = 0.66); HRI (n = 68), −2.93 vs. −4.31 (p pre-post difference = 0.04); statistically significant between LRI and HRI, p interaction <0.01
Ramaswami 2019 [76]	31	5 years	Pediatric male patients treated with 2 low-dose regimens (0.5 mg/kg q2w, *n* = 16; or 1.0 mg/kg q4w, *n* = 15) Mean, median (SD) [range] iGFR (mL/min/1.73 m^2^) ◦ Year 5 overall (*n* = 23), 115.7, 112.4 (15.4) [90.6, 149.1]; change from baseline to year 5, −3.4, 0.5 (12.7) [−35.9, 19.2], *p* = 0.4065; annualized rate of iGFR decline, −0.7, 0.1 (2.5) [−7.2, 3.9]; % change from baseline, −2.0, 0.3 (10.5) [−27.4, 18.7] Mean, median (SD) [range] eGFR (mL/min/1.73 m^2^) ◦ Year 5 overall (*n* = 27), 118.6, 121.0 (21.6) [81.1, 168.7]; change from baseline to year 5, −0.7, −4.6 (17.1) [−25.4, 58.3], *p* = 0.2667; annualized rate of eGFR decline, −0.1, −0.9 (3.4) [−5.1, 1.7]; % change from baseline, 0.8, −3.5 (16.4) [−20.1, 56.7]
Veloso 2023 * [69]	9	Mean (SD): 70.2 (12.9) months	Follow-up study of a family with a classic pathogenic variant (p.G35V) in Brazil eGFR and proteinuria/albuminuria remained stable during the follow-up in both sexes (further details not reported in abstract)
Wanner 2020 [77] Fabry Registry	86	Mean (SD) [range]: 3.9 (0.9) [2.0, 5.0] years	Self-controlled pretreatment and post-treatment comparison in adult female patients in the Fabry Registry Pretreatment vs. post-treatment eGFR slopes (95% CI) mL/min/1.73 m^2^/year overall and stratified by renal involvement or use of ACEI/ARBs ◦ Overall (*n* = 86): −0.83 (−1.52, −0.13) during pre-treatment and −0.95 (−1.59, −0.32) during post-treatment ◦ HRI (*n* = 22): eGFR slope pretreatment (95% CI), −1.95 (−3.23, −0.67), *p* < 0.01, *p* (pre-difference) = 0.10; eGFR slope post-treatment (95% CI), 2.08 (−3.21, −0.95), *p* < 0.01, *p* (post-difference) < 0.01; pretreatment vs. post-treatment eGFR slope difference (95% CI), −0.13 (−1.99, 1.72), *p* (pre-post difference) = 0.89, *p* (interaction) = 0.58 ◦ LRI (*n* = 53): eGFR slope pretreatment (95% CI), −0.68 (−1.56, 0.20), *p* = 0.13; eGFR slope post-treatment (95% CI), −0.14 (−0.92, 0.63), *p* (post-difference) = 0.72; pretreatment vs. post-treatment slope difference (95% CI), 0.54 (−0.81, 1.88), *p* (pre-post difference) = 0.43) ◦ ‘Ever’ ACEI/ARBs (*n* = 52): eGFR slope pretreatment (95% CI), −1.30 (−2.17, −0.43), *p* (pre-difference) = 0.08; eGFR slope post-treatment (95% CI), −1.19 (−1.96, −0.43), *p* = < 0.01, *p* (post-difference) 0.38; pretreatment vs. post-treatment eGFR slope difference (95% CI), 0.10 (−1.13, 1.34), *p* (pre-post difference) 0.87, *p* (interaction) = 0.55 ◦ ‘Never’ ACEI/ARBs (*n* = 34): eGFR slope pretreatment (95% CI), 0.01 (−1.15, 1.18), *p* = 0.98; slope post-treatment (95% CI), −0.57 (−1.69, 0.56), *p* = 0.32; pretreatment vs. post-treatment slope difference (95% CI), −0.58 (−2.43, 1.27), *p* (pre-post difference) = 0.54
Warnock 2012 [78] Fabry Registry	213	≥2 years	Observational study using data from the Fabry Registry Multivariate logistic regression analyses to identify factors associated with renal disease progression Patients were categorized into quartiles based on eGFR slopes during treatment. ◦ Men’s eGFR slope (mL/min/1.73 m^2^/year) categories: quartile 1, −1.2 to 15.3; quartile 2, −2.8 to 1.3; quartile 3, −4.8 to 2.9; and quartile 4, −15.5 to −14.9. ◦ Women’s eGFR slope (mL/min/1.73 m^2^/year) categories: quartile 1, 1.1 to 7.5; quartile 2, −0.4 to 1.0; quartile 3, −2.3 to −0.4; and quartile 4, −7.6 to −2.4 Mean, median (SD) [IQR] pre-treatment (baseline) eGFR (mL/min/1.73 m^2^) ◦ Men: eGFR slope quartile 1 (*n* = 37), 99, 104 (22.6) [89, 115]; eGFR slope quartile 2 (*n* = 37), 77, 73 (32.9) [46, 108]; eGFR slope quartile 3 (*n* = 38), 88, 88 (38.7) [51, 123]; eGFR slope quartile 4 (*n* = 36), 72, 61 (34.3) [47, 95] ◦ Women: eGFR slope quartile 1 (*n* = 14): 88, 87 (16.0) [75, 100]; eGFR slope quartile 2 (*n* = 14): 90, 93 (23.9) [72, 111]; eGFR slope quartile 3 (*n* = 16): 95, 98 (27.6) [76, 118]; eGFR slope quartile 4 (*n* = 14): 78, 85 (35.6) [36, 105] Mean, median (SD) [IQR] eGFR slope (mL/min/1.73 m^2^/year) ◦ Men: eGFR slope quartile 1 (*n* = 38): −0.1, −0.5 (1.20) [−0.9, 0.6]; eGFR slope quartile 2 (*n* = 38), −2.1, −2.1 (0.52) [−2.6, −1.5]; eGFR slope quartile 3 (*n* = 38), −3.8, −3.6 (0.61) [−4.3, −3.3]; eGFR slope quartile 4 (*n* = 37), −6.7, −6.2 (2.26) [−6.9, −5.5] ◦ Women: eGFR slope quartile 1 (*n* = 15), 2.7, 2.6 (1.65) [1.3– 3.4]; eGFR slope quartile 2 (*n* = 16), 0.1, 0.1 (0.41) [−0.2, 0.4]; eGFR slope quartile 3 (*n* = 16), −1.3,−1.4 (0.66) [−1.7, −0.6]; eGFR slope quartile 4 (*n* = 15), −4.4, −4.2 (1.58) [−5.5, −3.0] The risk factor most strongly associated with renal disease progression was UPCR ≥ 1 g/g (OR: 112; 95% CI: 4, 3109; *p* = 0.0054) Longer time from symptom onset to treatment was also associated with renal disease progression (OR: 19; 95% CI: 2, 184; *p* = 0.0098)
Warnock 2015 [79] FAACET	24	Median [IQR] duration of therapy before first visit: 3.1 [0.3, 4.4] years Patients were followed up during a 21-month treatment phase	The FAACET study evaluated the safety and efficacy of proteinuria control with ACEI and/or ARB therapy in patients with FD receiving ERT with agalsidase beta Assessment of renal function in patients with classic FD stratified by achieved UPCR goal (defined as UPCR for first visit ≤ 0.5 g/g or averaged treatment UPCR ≤ 50% of baseline or averaged treatment UPCR ≤ 0.5 g/g) eGFR slope (mL/min/1.73 m^2^/year) ◦ Patients who met UPCR goal (*n* = 18), eGFR slope (95% CI) −3.6 (−4.8, −1.1) ◦ Patients who did not meet UPCR goal (*n* = 6), eGFR slope (95% CI) −7.0 (−5.6, −2.0) Median [IQR] eGFR at initial follow-up visit (mL/min/1.73 m^2^): ◦ Patients who met UPCR goal and eGFR slope ≥ −2 mL/min/1.73 m^2^/year (*n* = 6): 88 [56, 11] ◦ Patients who met UPCR goal and eGFR slope < −2 mL/min/1.73 m^2^/year (*n* = 12): 62 [48, 78] ◦ Patients who were above UPCR goal and eGFR slope < −2 mL/min/1.73 m^2^/year; (*n* = 6), 61 [45, 81] Median (IQR) eGFR—average of follow-up visits (mL/min/1.73 m^2^): ◦ Patients who met UPCR goal and eGFR slope ≥ −2 mL/min/1.73 m^2^/year (*n* = 6): 80 [75, 126] ◦ Patients who met UPCR goal and eGFR slope < −2 mL/min/1.73 m^2^/year (*n* = 12): 56 [45, 75] ◦ Patients who were above UPCR goal and eGFR slope < −2 mL/min/1.73 m^2^/year (*n* = 6): 68 [45, 73] Median (IQR) eGFR at last follow-up visit (mL/min/1.73 m^2^): ◦ Patients who met UPCR goal and eGFR slope ≥ −2 mL/min/1.73 m^2^/year (*n* = 6): 97 [75, 121] ◦ Patients who met the UPCR goal (and eGFR slope < −2 mL/min/1.73 m^2^/year (*n* = 12): 62 [51, 85] ◦ Patients who were above UPCR goal and eGFR slope < −2 mL/min/1.73 m^2^/year (*n* = 6): 47 [35, 90]
Weidemann 2013 [80]	40	Median [IQR]: 6.0 [5.1, 7.2] years	Assessment of long-term effects of agalsidase beta in patients with advanced FD at a single center in Germany Mean (SD) GFR (mL/min): baseline, 85 (28); at follow-up, 73 (39) (*p* = 0.003) Mean (SD) annual change in GFR −2.3 (4.6) mL/min/year (men: −2.4 (5.0) mL/min/1.73 m^2^/year and women: −1.9 (3.3) mL/min/year)
Wraith 2008 [81]	16	48 weeks	Open-label study of pediatric patients with FD from France, Poland UK, and USA Mean (SD) eGFR (mL/min/1.73 m^2^): baseline (*n* = 16), 126 (29); week 24 (*n* = 16), 128 (29); week 48 (*n* = 15), 125 (26)
**(B)**			
**Author Year** **Study Identifier**	**Treatment Groups, *n*** **Treatment Duration**		**Key Results**
**Comparator studies**			
Arends 2018 [29]	Agalsidase alfa: 248 Agalsidase beta: 139 Median [range]: All patients 4.9 [0.8, 14.4] years; Agalsidase alfa 5.2 [0.8. 14.4] years; Agalsidase beta 3.8 [0.8, 12.1] years		Retrospective cohort study involving three European centers combined with data from the Canadian FD Initiative Median eGFR [range], mL/min/1.73 m^2^ at baseline: agalsidase alfa, 89 [10, 159]; agalsidase beta, 86 [10, 140] Adjusted for sex and phenotype, there was no difference in eGFR slope between agalsidase alfa and beta in patients with a baseline eGFR ≥ 60 mL/min/1.73 m^2^ (beta slope alfa-beta: −0.12 mL/min/1.73 m^2^/year; 95% CI: −0.76, 0.51; *p* = 0.70) In patients with an eGFR < 60 mL/min/1.73 m^2^, there was no difference in the rate of decline (beta slope alfa-beta: −0.85 mL/min/1.73 m^2^/year, 95% CI: −2.31, 0.62; *p* = 0.26), and adding the use of ACEI/ARBs and/or presence of proteinuria at baseline as covariates to the model did not result in a better fit or different results
Beck 2015 [112] FOS	Agalsidase alfa: 740 Published findings for untreated patients Median [range]: 5.4 [1.5, 13.7] years		Mean annualized eGFR slope (SEM) [95% CI], mL/min/1.73 m^2^/year FOS evaluable treated renal cohort by baseline eGFR category (mL/min/1.73 m^2^): ◦ Males, ≥ 60 (*n* = 117): −1.68 (0.19) [−2.05, −1.31]; <60 (*n* = 18): −2.86 (0.53) [−3.90, −1.83] ◦ Females, ≥ 60 (*n* = 111): −0.43 (0.21) [−0.83, −0.02]; <60 (*n* = 22): 0.36 (0.42) [−0.47, 1.19] Previously published external untreated cohort by baseline eGFR category (mL/min/1.73 m^2^): ◦ Males, ≥60 (*n* = 117): −3.0 (0.1); <60 (*n* = 28): −6.8 (1.5) ◦ Females, ≥60 (*n* = 42): −0.9 (0.9); females, <60: (*n* = 13); −2.1 (1.6) In a sensitivity analysis, eGFR results were evaluated only for FOS ERT patients who had 5-year data (79 males, 71 females; median [range] treatment duration, 8.25 [2.7, 13.8] years) ◦ The annualized eGFR slopes for these patients, overall and grouped by baseline eGFR, were not significantly different from corresponding values for the full FOS ERT renal cohort
Cybulla 2009 [113] FOS	Kidney transplant recipients: 27 (20 received agalsidase alfa, 7 untreated) Median ERT treatment duration: 3.5 years		Baseline renal function data available for 27 kidney transplant recipients (*n* = 27): median [IQR] eGFR (mL/min/1.73 m^2^) 44.4 [33.6, 64.0] Renal function in kidney transplant recipients receiving ERT or without ERT, medians [IQR] eGFR (mL/min/1.73 m^2^): ◦ Kidney recipients receiving ERT (*n* = 20), 59.2 [36.5, 65.2] ◦ Kidney recipients without ERT (*n* = 7), 35.1 [19.9, 51.7]
Cybulla 2013 * [115] FOS	Kidney transplant recipients: 93 (78 treated with ERT before or after transplant surgery, 15 untreated) Mean (SD): 3.7 (3.1) years		Mean (SD) baseline eGFR (mL/min/1.73 m^2^) ◦ Kidney recipients receiving ERT (*n* = 53): 44.8 (25.9) ◦ Kidney recipients without ERT (*n* = 10): 61.7 (12.0) Mean (95% CI) eGFR slope (mL/min/1.73 m^2^/year) ◦ Male recipients receiving ERT: −0.0 (−2.7, 2.6) ◦ Male recipients without ERT: −4.5 (−14.1, 5.0) ◦ Female recipients receiving ERT: −1.0 (−10.4, 8.4) ◦ Female recipients without ERT: −2.8 (−24.5, 18.8)
Cybulla 2014 * [114] FOS	Kidney transplant recipients: 51 (47 treated with ERT before or after transplant surgery, 4 untreated) Median [range]: 2.9 [0.0, 13.4] years		Mean [range] baseline eGFR (mL/min/1.73m^2^) ◦ Kidney recipients receiving ERT (*n* = 24): 45.7 [4.1, 110.8] ◦ Kidney recipients without ERT (*n* = 3): 54.2 [45.9, 59.7]
Dehout 2003 [116] FOS	Agalsidase alfa: 234, Untreated: 103 12 months		Observational study using preliminary data from FOS Patients with baseline GFR 60–89 mL/min (*n* = 39): a significant improvement in GFR from baseline, *p* = 0.027 following 12 months of treatment with agalsidase alfa Patients with baseline GFR 30–59 mL/min (*n* = *NR*): GFR was maintained after 12 months of treatment with agalsidase alfa, whereas in those without treatment, GFR declined significantly (*p* < 0.021) Patients with a baseline GFR > 90 mL/min (*n* = 51): no difference between treated and untreated patients after 1 year
Golan 2015 [118]	Agalsidase alfa 0.2 mg/kg EOW: 20 Agalsidase alfa 0.2 mg/kg weekly: 19 Agalsidase alfa 0.4 mg/kg weekly: 5 1 year		Phase 3/4 multi-center open-label study Mean baseline eGFR (mL/min/1.73 m^2^) ◦ 0.2 mg/kg EOW (*n* = 20): 82.0 ◦ 0.2 mg/kg weekly (*n* = 19): 78.1 ◦ 0.4 mg/kg weekly (*n* = 5): 72.1 Median baseline UACR ◦ 0.2 mg/kg EOW (*n* = 20): 41.5 ◦ 0.2 mg/kg weekly (*n* = 19): 40.0 ◦ 0.4 mg/kg weekly (*n* = 5): 250.0 Week 53 mean eGFR (mL/min/1.73 m^2^) ◦ 0.2 mg/kg EOW (*n* = 17): 78.3 ◦ 0.2 mg/kg weekly (*n* = 18): 77.5 ◦ 0.4 mg/kg weekly (*n* = 5): 70.4 Change in eGFR (95% CI) at week 53 (mL/min/1.73 m^2^) ◦ 0.2 mg/kg EOW: −1.21 (−7.46, 5.05) ◦ 0.2 mg/kg weekly: −3.32 (−9.63, 2.99) ◦ 0.4 mg/kg weekly: −1.68 (−14.00, 10.64) 0.2 mg/kg weekly minus EOW mean eGFR (mL/min/1.73 m^2^) ◦ LSM difference: −1.56 (95% CI: −9.23, 6.12); unadjusted *p* = 0.6830 Mean (95% CI) change from baseline in eGFR by CKD stage, 1A, 1B, 2, 3, 4/5 (mL/min/1.73 m^2^) ◦ 0.2 mg/kg EOW: CKD-1A, −24.2 (−82.6, 34.2; *n* = 3); CKD-1B, 1.6 (−35.4, 38.7; *n* = 3); CKD-2, 4.5 (−1.2, 10.1; *n* = 10); CKD-3, −5.0 (−27.3, 17.2; *n* = 3); CKD-4/5, −1.2 (−7.5, 5.0; *n* = 1) ◦ 0.2 mg/kg weekly: CKD-1A, −24.4 (−71.4, 22.6; *n* = 2); CKD-1B, −4.3 (−21.4, 12.8; *n* = 5); CKD-2, −1.2 (−8.4, 5.9; *n* = 7); CKD-3, 7.3 (−24.6, 39.2; *n* = 3); CKD-4/5, −2.9 (*n* = 2) ◦ 0.4 mg/kg weekly: CKD-1A, NA (*n* = 0); CKD-1B, −0.4 (*n* = 1); CKD-2, −2.3 (−37.0, 32.4; *n* = 3); CKD-3, NA (*n* = 0); CKD-4/5, −1.0 (*n* = 1)
Guerard 2018 [119]	Lucerastat + ERT: 10 ERT only: 4 12 weeks Mean (SD) previous ERT at baseline: Lucerastat: 4.5 (2.6) years; ERT only: 6.3 (4.2) years		Single-center, open-label, randomized study in Germany Mean (SD) baseline eGFR (mL/min/1.73 m^2^) ◦ Lucerastat + ERT (*n* = 10): 88.7 (26.2) ◦ ERT only (*n* = 4): 94.9 (26.7) Week 4 mean (SD) eGFR ◦ Lucerastat + ERT (*n* = 10): 97.4 (26.0) ◦ ERT only (*n* = 4): 91.8 (20.5) Week 8 mean (SD) eGFR ◦ Lucerastat + ERT (*n* = 10): 90.3 (21.6) ◦ ERT only (*n* = 4): 88.8 (23.8) Week 12 mean (SD) eGFR ◦ Lucerastat + ERT (*n* = 10): 92.9 (23.1) ◦ ERT only (*n* = 4): 95.0 (24.1)
Hughes 2008 [121]	Agalsidase alfa: 7 Placebo: 8 Randomized phase: 6 months OLE: 2 years		Placebo-controlled study followed by open-label extension for 2 years Mean baseline GFR showed a statistically significant increase within the normal range (106–131 mL/min; *p* = 0.007) in the drug group, whereas the placebo groups did not show any statistically significant change throughout the 6-month study
Hughes 2019 * [120]	Agalsidase alfa: 21 Untreated: 52 NR		Mean (SD) eGFR changes in women with FD from before to after pregnancy (mL/min/1.73 m^2^) ◦ ERT: −1.5 (11.7) ◦ Untreated: −3.1 (13.6)
Jovanovic 2017 * [122] FACETS ATTRACT	FACETS: Migalastat or placebo: 67; OLE, migalastat: 54, 24 months ATTRACT: Migalastat or ERT: 52, 18 months Efficacy analyses focused on patients with amenable mutations, FACETS: (*n* = 41); ATTRACT: (*n* = 34)		Over 24 months in FACETS, the annualized rate of change in eGFR CKD-EPI (SEM) with migalastat was −0.3 (0.664) mL/min/1.73 m^2^ The annualized rate of change (SEM) for eGFR in the range of 60 to <90 mL/min/1.73 m^2^ was −0.8 (1.01), which was comparable to the rate in the range of ≥90 mL/min/1.73 m^2^, 0.2 (0.90) During the 18-month controlled period of ATTRACT, the annualized means (SD) for eGFR for migalastat and ERT were comparable, −0.40 (4.3) and −1.03 (7.4), respectively The annualized rates of change in eGFR (SEM) in the range of 60 to <90 mL/min/1.73 m^2^ for migalastat and ERT were −0.2 (1.25) and −7.3 (6.01), respectively; the annualized rates in the range of ≥90 mL/min/1.73 m^2^ were −2.0 (0.57) and −2.1 (1.60) During the 12-month OLE, eGFR remained stable
Prabakaran 2014 [123]	Agalsidase beta/agalsidase alfa: 13 Untreated: 4 ≤6 years		Median [range] baseline eGFR (mL/min/1.73 m^2^) ◦ ERT-treated: 100 [64, 159] ◦ Untreated: 99 [88, 124] Mean annualized (95% CI) change in eGFR, (mL/min/1.73 m^2^) ◦ ERT-treated: −1.45 (95% CI: −0.31, −2.58; *p* = 0.012) ◦ Untreated: −0.89 (95% CI: −1.51, 3.30; *p* = 0.47) ◦ Decline in the two groups was not significantly different, *p* = 0.68
Schiffmann 2018 * [124]	Migalastat cohort: 52 Natural history cohort: 90 (untreated patients) 3–5 years		Mean annualized (SE) eGFR rate of change (mL/min/1.73 m^2^) by baseline 24-h urine protein (mg/24 h) ◦ Migalastat males - <100 (*n* = 3): 1.2 (1.2) - 100–1000 (*n* = 16): 0.9 (1.0) - 1000 (*n* = 2): −4.3 (0.1) ◦ Migalastat females - <100 (*n* = 9): −0.9 (0.5) - 100–1000 (*n* = 19): 1.3 (1.5) - 1000 (*n* = 3): −1.7 (1.1) ◦ Natural history cohort males - <100 (*n* = 18): −1.6 (1.5) - 100–1000 (*n* = 21): −3.3 (1.8) - 1000 (*n* = 22): −6.9 (1.5) ◦ Natural history cohort females - <100 (*n* = 7): −0.6 (2.6) - 100–1000 (*n* = 17): −2.2 (2.2) - >1 000 (*n* = 5): −4.6 (2.3)
Sirrs 2018 * [27]	Agalsidase alfa 0.2 mg/kg: 76 Agalsidase beta 1 mg/kg: 56 Median [range]: 99 [5, 123] months		A prospective multi-center study of ERT in Canada Males: rate of decline of eGFR (mL/min/1.73 m^2^/year) tended to be lower in patients on agalsidase beta than those on agalsidase alfa: −1.98 vs. −4.15, respectively; *p* = 0.09 Females: no difference in the rate of decline in eGFR was seen between the two treatment groups in females
Skuban 2017 * [108] FACETS ATTRACT	FACETS: Migalastat or placebo: 67 OLE, migalastat: 60 FACETS: 6-month double-blind followed by open-label migalastat from 6 to 12 months (stage 2) plus an additional year ATTRACT: Migalastat or ERT: 53.18 months		Analysis of 2 randomized phase 3 clinical studies FACETS: mean annualized rate of change (SEM) over 24 months in eGFR CKD-EPI with migalastat was −0.3 (0.7) mL/min/1.73 m^2^ ATTRACT: migalastat and ERT had comparable effects on renal function during the 18-month controlled period Compared with natural history, the rate of GFR decline in FACETS was slower than that of untreated patients when matched for sex and baseline proteinuria
van der Veen 2022 [33]	Agalsidase beta 0.5 mg/kg: 3 Agalsidase beta 1 mg/kg: 4 Untreated: 23 10 years		Cross-sectional retrospective study comparing outcomes in patients with classic FD after 10 years of ERT to a group of untreated patients with the same phenotype of comparable age Median [range] eGFR (mL/min/1.73 m^2^) ◦ Treated: 116 [92, 132] ◦ Untreated: 116 [46, 165]; *p* = 0.96 Reduced renal function (eGFR < 90 mL/min/1.73 m^2^) ◦ Treated: *n* = 0 ◦ Untreated: *n* = 4 Hyperfiltration (eGFR > 140 mL/min/1.73 m^2^) ◦ Treated: *n* = 0 ◦ Untreated: *n* = 3
Vedder 2007 [28]	Agalsidase alfa: 18 Agalsidase beta: 16 24 months		Median (SD) [range] GFR (mL/min) ◦ 12 months: agalsidase beta, 111 (30) [56, 159]; agalsidase alfa, 102 (42) [16, 150] ◦ 24 months: agalsidase beta, 107 (31) [49, 144]; agalsidase alfa, 100 (40) [14, 158] ◦ No significant differences between groups or from baseline in either group
Wallace 2022 * [125], Wallace 2024 [127] NCT02795676	Pegunigalsidase alfa: 52 Agalsidase beta: 25 24 months		Median [range] eGFR at baseline (mL/min/1.73 m^2^) ◦ Pegunigalsidase alfa: 73.5 [30.2, 125.9] ◦ Agalsidase beta: 74.9 [34.1, 107.6] ◦ Overall: 74.5 [30.2, 125.9] (*p* = 0.82) Median eGFR change from baseline at 24 months (mL/min/1.73 m^2^) ◦ Pegunigalsidase alfa: −2.39 ◦ Agalsidase alfa: −3.20 Median (95% CI) annual eGFR slope at 24 months (mL/min/1.73 m^2^/year) ◦ Pegunigalsidase alfa (*n* = 52): −2.51 (−3.79, −1.24) ◦ Agalsidase beta: (*n* = 25): −2.16 (−3.81, −0) ◦ Difference: −0.36 (−2.44, 1.73) ◦ Males - Pegunigalsidase alfa (n = 28): −3.44 (−5.38, −1.73) - Agalsidase beta (n = 18): −2. 01 (−3.98, −0.04) - Difference: −1.43 (−3.96, 1.10) ◦ Females - Pegunigalsidase alfa (n = 23): −1.15 (−3.11, 0.81) - Agalsidase beta (n = 7): −2.79 (−6.28, 0.70) - Difference: 1.64 (−2.56, 5.84)
Eng 2001 [117], Wilcox 2004 [126]	Agalsidase beta: 29 Placebo: 29 Initial trial: 20 weeks Follow-up: 30–36 months		Mean baseline (SD) eGFR (mL/min) ◦ Agalsidase beta: 83.0 (22.0) ◦ Placebo: 96.6 (35.5) Mean eGFR at start of extension study (mL/min/1.73 m^2^) ◦ Agalsidase beta/agalsidase beta: 109.6 ◦ Placebo/agalsidase beta: 138.3 Mean eGFR at 30 months of extension study (mL/min/1.73 m^2^) ◦ Agalsidase beta/agalsidase beta: 107.1 ◦ Placebo/agalsidase beta: 129.5 Patients with (<90 mL/min/1.73 m^2^) at baseline (*n* = 10): ◦ Mean (SD) eGFR (mL/min/1.73 m^2^) ◦ Agalsidase beta: 68.6 (19.7) at baseline; and 79.6 (6.3) at 30–36 months after treatment ◦ After 30–36 months of agalsidase beta treatment, 7 (70%) of the 10 patients had either stabilized their eGFR values (GFR: ± 20%) or improved

* Congress abstract. ACEI, angiotensin-converting enzyme inhibitor. aGAL, α-galactosidase A. ARB, angiotensin receptor blocker. CI, confidence interval. CKD, chronic kidney disease. CKD-EPI, Chronic Kidney Disease Epidemiology Collaboration. eGFR, estimated glomerular filtration rate. EOW, every other week. ERT, enzyme replacement therapy. ESRD, end-stage renal disease. FD, Fabry disease. FOS, Fabry Outcome Survey. GFR, glomerular filtration rate. HRI, high renal involvement. iGFR, iohexol glomerular filtration rate. IgG, immunoglobulin G. IQR, interquartile range. LRI, low renal involvement. LSM, least-squares mean. MDRD, Modification of Diet in Renal Disease. NA, not applicable. NR, not reported. NS, not significant. OLE, open-label extension. q2w, every 2 weeks. q4w, every 4 weeks. SD, standard deviation. SE, standard error. SEM, standard error of the mean. UACR, urine albumin-to-creatinine ratio. UPCR, urine protein-to-creatinine ratio.

### 3.2. Cardiac Outcomes

Cardiovascular disease is a leading cause of death in patients with FD. Cardiac manifestations of FD include left ventricular hypertrophy (LVH), diastolic dysfunction, valvular irregularities, and conduction disturbances [1]. Publications reporting LVMI data comprised: single-arm studies of agalsidase alfa (*n* = 19) [39,40,41,42,46,47,48,50,51,52,55,61,63,65,66,128,141,142,143], agalsidase beta (*n* = 11) [30,69,73,76,80,144,145,146,147,148,149], mixed or non-specified ERT (*n* = 19) [31,35,36,85,86,88,89,90,91,93,150,151,152,153,154,155,156,157,158], and other treatments (*n* = 13) [95,98,99,100,101,103,104,105,106,107,108,111,159], comparator studies (*n* = 15) [29,33,37,112,118,119,121,160,161,162,163,164,165,166], and switch studies (*n* = 7) [14,128,129,130,134,135,139,140]. Most of the studies (63 studies in 69 publications) assessed LVMI using echocardiography. An overview of the LVMI data from agalsidase alfa and agalsidase beta single-arm studies and the comparator studies is presented in Table 2. Key results from the other types of studies are provided in Supplementary Table S6.

For the 19 publications reporting LVMI data from agalsidase alfa single-arm studies, treatment/follow-up duration varied widely, from 26 weeks to 13 years (Table 2A). Agalsidase alfa stabilized LVMI (i.e., a non-statistically significant change in LVMI or LVM over the follow-up period) in nine studies [42,46,50,52,55,61,66,128,143] and reduced LVMI in three others [39,63,141]. In addition, five studies showed improvement in or stabilization of LVMI for different subgroups [41,47,48,65,142]. A large post-marketing surveillance study of 495 patients in Japan found that treatment with agalsidase alfa for up to 8 years helped to stabilize LVMI in both females and males [52]. Early initiation of treatment with agalsidase alfa in children and adolescents maintained LVMI at pretreatment levels over several years of ERT [50,51,55,76]. In an analysis of FOS registry data (mean treatment duration: ~11 years), male patients with classic FD who started agalsidase alfa treatment before 25 years of age (*n* = 32) had a nonsignificant mean (standard error [SE]) annual increase in LVMI of 0.28 (0.30) g/m^2.7^, whereas those who started treatment at or after 25 years of age (*n* = 58) had a significant mean (SE) annual LVMI increase of 1.03 (0.21) g/m^2.7^ (*p* < 0.0001) [41]. Females with FD responded well to agalsidase alfa, with stable or improved LVMI observed over 4–10 years of treatment [47,48,63,142].

All 11 articles that reported the effects of agalsidase beta on LVMI in single-arm studies described a decrease or stabilization of LVMI over treatment periods ranging from 22 weeks to 5 years [30,69,73,76,80,144,145,146,147,148,149] (Table 2A). Three studies that were designed specifically to evaluate the effects of ERT with agalsidase beta on cardiac manifestations demonstrated statistically significant decreases in LVMI over treatment periods of 3–5 years [69,146,147]. Moreover, significant improvements in left ventricular morphology were seen in both males and females, and regardless of FD severity [147]. Analyses by baseline LVH (LVMI > 134 g/m^2^ in men and >110 g/m^2^ in women) showed that LVMI reduced significantly in those with baseline LVH (*n* = 42) and was stable in those without LVH (*n* = 24) over a median follow-up period of 3 years [147].

The majority of the single-arm studies (10/17) evaluating the effects of mixed or non-specified ERT showed stabilization of LVMI (Supplementary Table S6), with only three studies (two in Taiwanese patients) and one sub-analysis (adults with LVH) reporting a reduction in LVMI during ERT [31,86,151,155]. Most (10/12) of the single-arm studies for other treatments evaluated migalastat. Migalastat led to regression of LVMI in six studies [98,99,100,103,104,105,111] and stabilization in four studies [101,106,108,159] over treatment periods of 48 weeks to 5 years.

Multiple ERT studies assessing LVMI outcomes included a placebo or untreated control arm [33,37,121,156,161,162,165,166], two of which were meta-analyses of previously published data [162,164] (Table 2B). Reductions in LVMI with ERT were demonstrated in both adults [121,161] and adolescents [33], as well as among treatment-naive and treatment-experienced patients [162]. Only one study did not show a difference in LVMI between patients who received ERT (*n* = 47; median follow-up: 8 years) or no ERT (*n* = 19; median follow-up: 6 years) [37]. Early initiation of ERT before cardiac disease progression appeared to result in the best LVMI outcomes [156,166]. A randomized, controlled, open-label study found no differences between the efficacy of agalsidase alfa (*n* = 18) and agalsidase beta (*n* = 16) on LVM after 12 or 24 months of treatment [28]. In a retrospective cohort study, both agalsidase alfa and agalsidase beta reduced LVMI after 1 year in patients with LVH at baseline (*n* = 168), with no significant difference between the treatment groups [29]. A greater proportion of patients treated with agalsidase beta than agalsidase alfa had a decrease in LVMI over that period (79% vs. 62%) [29]. In the placebo-controlled FACETS study (*n* = 67), reductions in LVMI with migalastat were sustained over 30 months in the open-label extension (mean [95% CI] change from baseline: −17.0 [−26.2, −7.9] g/m^2^; *n* = 15), including in patients with baseline LVH [160].

The effect of treatment switching on LVMI was assessed in three clinical trials and two observational studies (Supplementary Table S6). In a prospective clinical trial, LVMI was maintained after 24 months in patients who switched from agalsidase beta to agalsidase alfa (*n* = 71) [128]. No change in LVMI was observed over 12 months in men (*n* = 15) who switched from agalsidase alfa to pegunigalsidase alfa in the phase 3 BRIDGE trial [139]. In the phase 3 ATTRACT study (*n* = 36), switching from ERT to migalastat was associated with a mean (95% CI) change in LVMI of −6.6 (−11.0, −2.2) g/m^2^ after 18 months of treatment [129,130]. However, the inclusion of a broad patient age range (18–72 years) with mild *GLA* variants associated with residual enzyme activity, may have influenced these results because some patients in this population would not have developed early-onset or severe disease symptoms [167].

Identified studies that assessed other cardiac structure outcomes (e.g., left ventricular posterior wall thickness, interventricular septal thickness, and left ventricular wall thickness) in patients receiving FD treatments are shown in Supplementary Table S7. These included single-arm studies of agalsidase alfa (*n* = 6) [39,42,46,47,48,61], agalsidase beta (*n* = 13) [70,73,75,76,77,80,82,144,145,146,147,149,168], mixed or non-specified ERT (*n* = 9) [85,86,88,89,93,132,152,153,157,163], and other treatments (*n* = 3, all on migalastat) [98,135,159]. There were seven publications of comparator studies [37,156,158,161,165,169,170] and three publications describing switch studies [129,131,140]. Overall, ERT stabilized cardiac structural changes associated with FD and improved outcomes in patients with LVH. As observed for LVMI, early treatment initiation was more effective at preventing progression than interventions during established disease.

**Table 2:** Overview of LVMI data from (**A**) single-arm agalsidase alfa or agalsidase beta studies and (**B**) comparator studies.

(A)			
Author Year	*N*	Treatment Duration Assessment Method	Key Results
Agalsidase alfa single-arm studies			
Baehner 2003 [141]	15	Up to 55 weeks Echocardiography	In heterozygous female patients with FD, mean (SEM) change from baseline in LVMI (g/m^2^) ◦ Week 13 (*n* = 15): −5.5 (5.94); *p* = 0.372 ◦ Week 27 (*n* = 11): −23.0 (5.78); *p* = 0.003 ◦ Week 41 (*n* = 7): −25.2 (8.12); *p* = 0.021
Beck 2004 [39]	188	Mean [max]: 17 [56] months Echocardiography	In a population of patients with FD who were 52% male, echocardiographic data were available for 52/188 (28%) patients treated for 1 year and for 17/92 (18%) patients treated for 2 years Individual data on mean LVM from serial echocardiographic examinations of patients with LVH (LVM > 50 g/m^2.7^) indicate a decrease in height-adjusted LVM, particularly in those patients with the greatest degree of hypertrophy Overall, there was a significant (*p* < 0.05) decrease in LVM at 1 and 2 years of treatment with agalsidase alfa
Giugliani 2023 * [40]	66	Median [IQR]: 20.0 [19.6, 20.6] NR	Observational study using data from FOS Mean (SD) baseline LVMI: 49.79 (14.23) g/m^2.7^ Mean (SE) annual rate of change (*n* = 31): 0.83 (0.32) g/m^2.7^ Annual increase ◦ Males: 0.65 g/m^2.7^ ◦ Females: 1.34 g/m^2.7^
Goker-Alpan 2015 [128]	100	24 months Echocardiography	A large cohort study of patients who were started on or switched to agalsidase alfa during a worldwide shortage of agalsidase beta Number of patients with or without baseline LVH ◦ Naive to ERT (*n* = 29): with LVH, 10 (34.5%); without LVH, 15 (51.7%); not determined, 4 (13.8%) ◦ Switch from agalsidase beta to agalsidase alfa (n = 71): with LVH, 27 (38.0%); without LVH, 25 (35.2%); not determined, 19 (26.8%) Mean LVMI did not change significantly in any patient subgroup, including those with or without LVH at baseline Cardiac data presented in figures
Goker-Alpan 2016 [46]	14	Median [range]: 54.5 [54.0, 59.0] weeks Echocardiography	An open-label safety study of agalsidase alfa in children naive to ERT LVMI values remained within the normal range throughout the study Mean [95% CI] change from baseline at week 55 ◦ Males (*n* = 5): −0.74 [−6.01, 4.53] g/m^2.7^ ◦ Females (*n* = 9): 0.66 [−4.77, 6.08] g/m^2.7^
Hughes 2009 * [142] FOS	250	4 years Echocardiography	Observational study using data from FOS Cohort of females (*n* = 78) and males (*n* = 172) followed for 4 years of ERT After 4 years of agalsidase alfa, there was a significant decrease (*p* = 0.0312) in mean LVM in females (*n* = 24), which was not apparent in males (*n* = 45)
Hughes 2011 [48] FOS	250	≥4 years Echocardiography	Observational study using data from FOS Mean (SD) LVMI (g/m^2.7^) at 4 years ◦ Females (*n* = 24): 43.7 (14.3); *p* = 0.031 vs. baseline ◦ Males (*n* = 45): 52.2 (19.2); *p* = 0.247 vs. baseline Males with LVH at baseline (*n* = 22): mean difference after 4 years, −9.11 g/m^2.7^; *p* = 0.0115 Males with normal LVMI at baseline (*n* = 23): mean difference after 4 years, −3.82 g/m^2.7^; *p* = 0.0741
Kampmann, 2009 [143]	45	30–42 months (defined as 36 months) Echocardiography	Retrospective, blinded, pooled analysis of 4 industry-sponsored clinical studies including patients with FD who had received≥ 36 months of ERT LVH was present in 14/45 patients at baseline LVMI (g/m^2.7^) at 12 months in patients with LVH at baseline (*n* = 9): ◦ Mean (SD) change from baseline: −9.2 (7.9) ◦ Median change from baseline: −8.9, *p* vs. baseline: 0.008 LVMI (g/m^2.7^) at 36 months in patients with LVH at baseline (*n* = 10): ◦ Mean (SD) change from baseline: −5.1 (7.5) ◦ Median change from baseline: −9.0, *p* vs. baseline: 0.037 LVMI (g/m^2.7^) at 12 months in patients without LVH at baseline (*n* = 28): ◦ Mean (SD) change from baseline: 3.6 (5.7) ◦ Median change from baseline: 3.2, *p* vs. baseline: 0.002 LVMI (g/m^2.7^) at 36 months in patients without LVH at baseline (*n* = 26): ◦ Mean (SD) change from baseline: 2.1 (7.9) ◦ Median change from baseline: 1.5, *p* vs. baseline: 0.27
Kampmann 2015 [47]	45	Median [range]: 10.8 [9.6, 12.5] years Echocardiography	A single-center observational study in Germany At start of treatment, LVMI values suggested a varying degree of LVH (LVMI ≥ 50 g/m^2.7^) ◦ Men: 71% (*n* = 15/21) ◦ Women: 67% (*n* = 16/24) After 1 year of treatment ◦ Males with LVH: marked improvement in LVMI; LS mean [95% CI] change, −16.46 [−23.81, −9.11] g/m^2.7^; *p* < 0.0001 ◦ Females with LVH: LS mean [95% CI] change, −16.69 [−23.62, −9.75] g/m^2.7^; *p* < 0.0001; sustained for 3 years and no longer significantly different from baseline after 10 years After 10 years of treatment ◦ Patients without baseline LVH: no change from baseline ◦ Males with LVH: LVMI was significantly reduced; LS mean [95% CI] change from baseline, −13.55 [−23.05, −4.06] g/m^2.7^; *p* = 0.0061 ◦ Females with LVH: LVMI was not significantly changed
Mehta 2009 [65] FOS	181	Mean (SD): 3.1 (2.1) years Echocardiography	Observational study using data from FOS Patients with baseline LVH Year 1 (*n* = 23): ◦ Mean baseline LVMI: 68.5 (20.0) g/m^2.7^ ◦ Mean LVMI at year end: 58.4 (19.6) g/m^2.7^ ◦ Mean change from baseline: − 10.1 (12.0) g/m^2.7^, *p* = 0.0006 Year 2 (*n* = 24) ◦ Mean baseline LVMI: 70.1 (19.7) g/m^2.7^ ◦ Mean LVMI at year end: 56.9 (16.7) g/m^2.7^ ◦ Mean change from baseline: −13.3 (17.2) g/m^2.7^, *p* = 0.001 Year 3 (*n* = 29): ◦ Mean baseline LVMI: 70.1 (21.2) g/m^2.7^ ◦ Mean LVMI at year end: 59.8 (18.0) g/m^2.7^ ◦ Mean change from baseline: −10.3 (20.8) g/m^2.7^, *p* = 0.0121 Year 5 (*n* = 32): ◦ Mean baseline LVMI: 71.4 (22.5) g/m^2.7^ ◦ Mean LVMI at year end: 64.1 (18.7) g/m^2.7^ ◦ Mean change from baseline: −7.3 (15.3) g/m^2.7^, *p* = 0.0111 Patients without baseline LVH Year 1 (*n* = 17): ◦ Mean baseline LVMI: 38.5 (6.5) g/m^2.7^ ◦ Mean LVMI at year end: 35.0 (5.7) g/m^2.7^ Mean change from baseline: −3.5 (6.8) g/m^2.7^, *p* = NS Year 2 (*n* = 18): ◦ Mean baseline LVMI: 38.9 (6.5) g/m^2.7^ ◦ Mean LVMI at year end: 35.6 (7.4) g/m^2.7^ ◦ Mean change from baseline: −3.4 (9.2) g/m^2.7^, *p* = NS Year 3 (*n* = 24): ◦ Mean baseline LVMI: 38.4 (6.2) g/m^2.7^ ◦ Mean LVMI at year end: 36.9 (6.9) g/m^2.7^ ◦ Mean change from baseline: −1.5 (8.5) g/m^2.7^, *p* = NS Year 5 (*n* = 25): ◦ Mean baseline LVMI: 38.7 (6.3) g/m^2.7^ ◦ Mean LVMI at year end: 40.5 (10.0) g/m^2.7^ ◦ Mean change from baseline: +1.8 (10.1) g/m^2.7^, *p* = NS
Pintos-Morell 2023 [41] FOS	285	Mean (SD): Early initiators group: 11.2 (5.7); Late initiators group: 11.8 (5.8) NR	Observational study using data from FOS Annual LVMI increase ◦ Early-initiators group (*n* = 32): - Mean (SE) increase: 0.28 (0.30) g/m^2.7^/year, *p* = 0.3457 ◦ Late-initiators group (*n* = 58): - Mean (SE) increase: 1.03 (0.21) g/m^2.7^/year, *p* < 0.0001 The rate of LVMI change was significantly different between the groups (*p* = 0.0419)
Ramaswami 2011 [50] FOS	8	Mean (SD): 4.2 (1.9) years Echocardiography	Observational study in pediatric patients using data from FOS Patients received their first dose of agalsidase alfa before reaching the age of 7 years Mean (SD) [range] LVMI (g/m^2.7^) ◦ Baseline (*n* = 6): 40.6 (6.51) [33.3, 52.3] ◦ At follow-up (*n* = 6): 31.8 (7.89) [22.4, 45.1]
Ramaswami 2012 [66] FOS	98	≥6 months Echocardiography	Observational study in pediatric patients (<18 years old) using data from FOS 12 months (*n* = 26): no change in LVMI observed in 26 children 24 months (*n* = 17): mean (SD) LVMI declined from 35.7 (5.7) at baseline g/m^2.7^ to 31.7 (5.6) g/m^2.7^
Ramaswami 2019 [42] FOS	69	Median [range] time from start of ERT to FOS data extraction (years): Evaluable treated cardiac cohort (*n* = 69): 13.6 [10.1–17.1]; Females (*n* = 34): 12.8 [10.1–16.5]; Males (*n* = 35): 14.3 [10.2–17.1] Echocardiography	Observational study using data from FOS Number of patients (%) with LVH (defined as LVMI > 48 g/m^2.7^ in females and LVMI > 50 g/m^2.7^ in males) ◦ Evaluable treated cardiac cohort: 32/69 (46.4%) ◦ Females: 18/34 (52.9%) ◦ Males: 14/35 (40.0%) Mean (SD) [95% CI] LVMI (g/m^2.7^) ◦ At baseline - Evaluable treated cardiac cohort (*n* = 69): 50.65 (16.90) - Females (*n* = 34): 52.21 (16.99) [46.3–58.1] - Males (*n* = 35): 49.13 (16.92) [43.3–54.9] ◦ At year 10 - Females (*n* = 34): 58.8 [48.7–68.9] - Males (*n* = 35): 52.0 [43.2–60.8] Mean [95% CI] LVMI slope (g/m^2.7^/year) ◦ Patients without LVH at baseline - Females (*n* = 16): 0.52 [−0.13, 1.17] - Males (*n* = 21): 0.57 [0.02, 1.13] ◦ Patients with LVH at baseline - Females (*n* = 18): 1.51 [0.91, 2.12] - Males (*n* = 14): 0.87 [0.19, 1.55] Mean [95% CI] LVMI (g/m^2.7^) among cardiac cohort patients without renal involvement (eGFR > 90 mL/min/1.73 m^2^ and urinary protein <0.5 g/day) at baseline ◦ At baseline - Females (*n* = 6): 43.3 [27.1, 59.5] - Males (*n* = 10): 39.3 [34.1, 44.5] ◦ At year 10 - Females (*n* = 6): 45.9 [22.9, 69.0] - Males (*n* = 10): 43.2 [36.3, 50.1]
Ries 2006 [51]	24	26 weeks Echocardiography	Open-label multi-center study in pediatric patients Pediatric patients naive to ERT Mean (SEM) LVMI (g/m^2.7^) at baseline ◦ Boys (*n* = 19): 32.4 (1.3) ◦ Girls (*n* = 5): 36.0 (4.0) Mean (SEM) LVMI (g/m^2.7^) after 25 weeks of treatment ◦ Boys: 31.4 (1.4) ◦ Girls: 32.8 (2.3), *p* vs. baseline NS Children (*n* = 3) with high–normal LVMI at baseline (>40 g/m^2.7^) had a mean decrease of 15% in LVM/h after 25 weeks of treatment
Sasa 2019 [52]	493	Mean [range]: 3.5 [0.0, 7.9] years Echocardiography	A post-marketing surveillance study involving patients with FD in Japan Mean (SD) LVMI at each year, mean (SD) change in LVMI (g/m^2.7^) from baseline *p* versus baseline ◦ Males (classic phenotype) - Year 0.5 (*n* = 43): 69.1 (45.7), 1.6 (13.6), *p* = 0.459 - Year 1 (*n* = 40): 64.9 (40.0), 0.6 (14.3), *p* = 0.777 - Year 2 (*n* = 35): 66.8 (42.2), 3.4 (20.0), *p* = 0.320 - Year 3 (*n* = 31): 69.8 (42.1), 3.3 (18.5), *p* = 0.328 - Year 4 (*n* = 23): 61.8 (38.2), 7.4 (15.7), *p* = 0.035 - Year 5 (*n* = 8): 68.9 (50.9), 15.0 (23.4), *p* = 0.112 - Year 6 (*n* = 4): 81.3 (73.2), 23.5 (34.7), *p* = 0.268 - Year 7 (*n* = 2): 122.8 (121.8), 51.7 (58.7), *p* = 0.430 - Year 8 (*n* = 1): 192.1 (NA), 76.4 (NA) ◦ Males (non-typical variant) - Year 0.5 (*n* = 23): 79.4 (39.6), −1.4 (13.9), *p* = 0.638 - Year 1 (*n* = 19): 79.9 (35.7), −5.3 (9.9), *p* = 0.031 - Year 2 (*n* = 15): 70.3 (26.7), −8.5 (16.1), *p* = 0.060 - Year 3 (*n* = 14): 68.8 (37.3), −4.2 (13.8), *p* = 0.271 - Year 4 (*n* = 14): 67.2 (33.2), −3.4 (10.3), *p* = 0.240 - Year 5 (*n* = 5): 75.4 (23.3), −0.2 (14.4), *p* = 0.973 - Year 6 (*n* = 2): 91.0 (63.5), 15.5 (16.5), *p* = 0.412 ◦ Females - Year 0.5 (*n* = 50): 65.9 (22.8), 1.8 (14.5), *p* = 0.384 - Year 1 (*n* = 45): 64.2 (25.1), 2.8 (12.7), *p* = 0.147 - Year 2 (*n* = 39): 66.9 (25.5), 2.7 (14.0), *p* = 0.240 - Year 3 (*n* = 27): 74.3 (27.1), 4.3 (16.4), *p* = 0.187 - Year 4 (*n* = 19): 71.3 (30.0), 2.6 (22.7), *p* = 0.626 - Year 5 (*n* = 7): 79.8 (34.0), 4.3 (30.8), *p* = 0.727
Schiffmann 2014 [55]	17	Mean (SD): 6.5 (0.6) years Echocardiography	Open-label multi-center extension study for pediatric patients who had completed a 26-week study This study represented the longest assessment of ERT in children with FD in a clinical trial setting Pediatric patients with FD (7–17 years of age at study enrollment) received 6 months of 0.2 mg/kg agalsidase alfa This study was divided into two phases, before (phase 1) and after (phase 2) a change in the agalsidase alfa manufacturing process For all 17 patients, baseline mean (SD) [range] LVMI (g/m^2.7^) measurements were within the normal range (upper limit of normal range: males = 51 g/m^2.7^; females = 48 g/m^2.7^): 30.66 (5.96) [22.7, 42.3] Through phase 1, LVMI decreased from baseline (mean change: −3.25 at week 185) LVMI continued to decrease through phase 2 (except week 104) Despite LVMI fluctuations observed in individual patients, levels remained below LVH criteria throughout the study
Tsuboi 2017 [61] Japan Fabry Research—002	36	Median [range]: 62.5 [8, 84] months Echocardiography	Prospective observational study in Japan Cohort included ERT treatment-naive patients and those who were receiving agalsidase alfa 0.2 mg/kg every 2 weeks At 60 months, LVMI had decreased from 50.3 at baseline to 46.6 g/m^2.7^
Whybra 2009 [63]	36	4 years Echocardiography	Prospective single-center open-label study of adult women in Germany A cohort of female patients with FD who were experiencing manifestations of FD and had not been previously treated with ERT ◦ LVH (defined as LVM ≥ 48 g/m^2.7^) at baseline (*n*/*N* [%]): 25/36 (69.4%) ◦ For patients with LVH, mean (SD) [range] LVM (g/m^2.7^) at baseline: 89.4 (42.1) [52.8, 201.1] Number of patients with LVH at baseline (*n* = 25) with decreases in LVM ◦ LVM decrease of >20%: 13/25 (52%) ◦ LVM decreases of 10–20%: 7/25 (28%) ◦ LVM decreases of <10%: 5/25 (20%) Mean (SD) LVMI (g/m^2.7^) over time by number of months of treatment and LVM classification (* *p* < 0.05 vs. baseline; ** *p* < 0.01 vs. baseline; *** *p* < 0.001 vs. baseline) ◦ LVM classification, > 85 (*n* = 9) - Month 12: 98.1 (31.1) *** - Month 24: 98.1 (31.7) *** - Month 36: 96.3 (34.0) *** - Month 48: 91.9 (29.7) *** ◦ LVM classification, > 60–85 (*n* = 9) - Month 12: 57.4 (13.3) *** - Month 24: 56.5 (14.6) *** - Month 36: 58.2 (13.2) *** - Month 48: 62.0 (17.7) *** ◦ LVM classification, > 48–60 (*n* = 8) - Month 12: 46.3 (4.7) *** - Month 24: 44.0 (5.2) *** - Month 36: 47.0 (2.8) *** - Month 48: 48.7 (4.1) *** ◦ LVM classification, <48 (*n* = 11) - Month 12: 36.2 (5.8) ** - Month 24: 35.3 (7.8) ** - Month 36: 37.0 (10.1) * - Month 48: 36.2 (7.1) **
**Agalsidase beta single-arm studies**			
Cabrera 2017 [30]	39	Mean (SD): 68.2 (36.9) months Echocardiography	Long-term prospective and historical analysis of a cohort from Argentina Mean (SD) LVMI in males slightly increased from 113.2 (45) to 127.4 (64) g/m^2^ 11 male patients had LVH at baseline In 4/11 males, baseline LVMI was reduced from 163.1 (64.7) to 123.4 (49.8) g/m^2^, 2 patients had a stabilized LVMI and 4 patients had an increased LVMI from 157.9 (32.3) to 261.6 (48.6) g/m^2^ 9 female patients had LVH at baseline, only 1 patient increased their LVMI from 158 to 204 g/m^2^; all remaining patients reduced or stabilized their LVMI All patients without LVH at baseline remained with a normal LVMI at the end of the study
Elliot 2006 [144]	5	Mean (SD): 10.1 (2.3) months Echocardiography	Males receiving 1 or 2 mg/kg agalsidase beta EOW LVMI did not change from baseline to follow-up: mean (SD) [range] change, 0.4 (37.8) [−66.3, 26.4] g/m^2^; *p* = NS
Hwang 2022 [73]	10	22-week follow-up Mean (SD) [range]: 4.6 (3.2) [1, 9] years Echocardiography	A phase 2 open-label study in Korea 8 patients had received previous ERT with agalsidase beta, 2 patients were treatment naive Mean (SD) LVMI at week 22: 111.66 (61.74) g/m^2^; change from baseline, 4.00 (13.44); *p* = 0.625
Kalliokoski 2006 [145]	10	12 months Echocardiography	Observational study in Finland Mean (SD) LVM (g) ◦ Before ERT: 275 (28) ◦ After 12 months of treatment: 292 (35); *p* = 0.27 Mean (SD) LVMI (g/m^2^) ◦ Before ERT: 151 (12) ◦ After 12 months of treatment: 156 (14); *p* = 0.37
Messalli 2012 [146]	16	48 months MRI	Observational study in Italy Study specifically on cardiac structures (MRI study) Baseline: mean (SD) LVM baseline (g), 187 (59) LVM: mean (SD), 149 (44) g; *p* < 0.001 vs. baseline
Motwani, 2012 [147]	66	Median [range]: 3 [2, 5] years Echocardiography	Observational study in the United Kingdom The overall mean (SD) LVMI was significantly reduced by ERT at follow-up (*n* = 66): 116 (28) vs. 113 (26) g/m^2^; *p* < 0.001 On sub-group analysis, this improvement was seen in both sexes and in both mild (FOS-MSSI ≤18) and moderate/severe (FOS-MSSI > 18) disease groups (all *p* < 0.05) In patients with LVH at baseline (*n* = 42), mean (SD) LVMI was significantly reduced by ERT 135 (13) vs. 133 (3) g/m^2^; *p* < 0.05 There was no significant change in mean (SD) LVMI in those patients without baseline LVH (*n* = 24): 82 (6) vs. 82 (5) g/m^2^; *p* > 0.05
Pisani 2005 [148]	9	24-months Echocardiography	Non-randomized, open-label, prospective study in male patients undergoing maintenance dialysis in the context of renal replacement therapy After 24 months of ERT (*n* = 9), LVMI did not change compared with basal values Pre- vs. post-changes in LVMI ◦ In the 24 months before ERT, LVMI increased in all patients, and during the 24 months of therapy, progression of LVMI decreased in all except 2 patients Average percentage of increase per year in LVMI was 6% during the 24 months preceding ERT and 3% during the 2 years of treatment This also was shown by the comparison (*p =* 0.06) of mean (SD) LVMI curve slopes for the pretreatment period 0.98 (0.017) g/height^2.7^ with those obtained during the treatment period 0.46 (0.960) g/height^2.7^
Ramaswami 2019 [76]	31	5 years Echocardiography	Open-label parallel-group phase 3b study in male pediatric patients treated with 2 low-dose regimens (0.5 mg/kg q2w, *n* = 16 or 1.0 mg/kg q4w, *n* = 15) Mean, median (SD) [range] LVMI, g/m^2.7^ ◦ Baseline (*n* = 23): 33.2, 32.0 (5.1) [20.5, 44.1] ◦ Year 5 (*n* = 24): 31.4, 31.9 (7.2) [22.1, 53.4]
Veloso 2023 * [69]	9	Mean (SD): 70.2 (12.9) months MRI and Echocardiography	Follow-up study of a family with FD with a classic pathogenic variant (*p*.G35V) in Brazil Female patients presented a mean reduction of LVMI of 9.73 g/m^2^ (95% CI: −26.9, 6.8); *p* = NR
Weidemann 2013 [80]	40	≥5 years Median [IQR]: 6.0 [5.1, 7.2] MRI and Echocardiography	Assessment of long-term effects of agalsidase beta in Germany Patients treated for at least 5 years with agalsidase beta Mean (SD) LVM (g) ◦ Baseline: 270 (87) ◦ At follow-up: 224 (71); *p* < 0.0001 vs. baseline
Wuest 2011 [149]	14	13 ± 1 months Echocardiography	Assessment of the effect of ERT on right ventricular cardiac morphology and function ◦ Baseline: mean (SD) normalized LVM, 102 (26) g/m^2^ ◦ At follow-up: mean (SD) normalized LVM, 94 (27) g/m^2^; *p* < 0.05 between baseline and follow-up
**(B)**			
**Author Year**	**Comparator Arms, *n*/*N***	**Treatment Duration** **Assessment Method**	**Key Results**
**Comparator studies**			
Arends 2018 [29]	Agalsidase alfa: 248 Agalsidase beta: 139	Median [range]: All patients: 4.9 [0.8, 14.4] years; Agalsidase alfa: 5.2 [0.8, 14.4] years; Agalsidase beta: 3.8 [0.8, 12.1] years Echocardiography	Retrospective cohort study involving three European centers and the Canadian FD Initiative ◦ In patients without LVH at baseline (*n* = 110), there was no change in LVMI after 1 year of ERT ◦ In patients with LVH (*n* = 168), there was a decrease after 1 year of treatment The magnitude of the decrease depended on the LVMI at baseline (*p* < 0.001) and was independent of sex and phenotype ◦ Patients with an LVMI above the reference value but <75 g/m^2.7^ treated with agalsidase beta showed a larger but non-significant decrease of LVMI over the first year compared with alfa (beta alfa-beta: −3.31 g/m^2.7^; 95% CI: −6.84, 0.23; *p* = 0.07), but no difference for the entire group was found (beta alfa-beta: −2.26 g/m^2.7^; 95% CI: −5.39, 0.87; *p* = 0.15) The decrease over the first year was followed by stabilization of LVMI in the following years (beta time on ERT: 0.22; *p* = 0.33) The analysis of the number of patients who showed a decrease in LVMI after 1 year of treatment revealed that treatment with agalsidase beta resulted in a higher proportion of patients with a decrease in LVMI compared with agalsidase alfa (79% vs. 62%) (OR: 2.27; 95% CI: 1.11, 4.86; *p* = 0.03), adjusted for the LVMI at baseline
Beck 2015 [112]	Agalsidase alfa: 164 Previously published untreated cohort: 78	Median [range]: 5.4 [1.5, 13.7] years Echocardiography	Retrospective comparisons with previously published data (agalsidase alfa-treated patients from the FOS registry with well-described cohorts of untreated individuals from previously published studies) Mean annualized LVMI slope (SEM) [95% CI], g/m^2.7^/year by baseline LVH status ◦ Evaluable treated cardiac cohort from FOS - Males ▪ Total (*n =* 71): 0.33 (0.10) [0.13, 0.53] ▪ LVH (*n =* 29): 0.19 (0.16) [−0.13, 0.50] ▪ No LVH (*n =* 42): 0.47 (0.13) [0.22, 0.72] Females ▪ Total (*n =* 93): 0.48 (0.09) [0.30, 0.66] ▪ LVH (*n =* 45): 0.77 (0.14) [0.49, 1.05] ▪ No LVH (*n =* 48): 0.19 (0.11) [−0.03, 0.41] Previously published external untreated comparator cohort - Males ▪ Total (*n =* 39): 4.07 (1.03) ▪ LVH (*n =* 18): 6.59 (8.5) ▪ No LVH: not available Females ▪ Total (*n =* 39): 2.31 (0.81) ▪ LVH (*n =* 15): 3.77 (7.7) ▪ No LVH: not available A sensitivity analysis evaluating available 5-year follow-up LVMI FOS data was conducted in 38 males and 40 females (median [range] treatment duration, 8.45 [0.9, 11.7] years) ◦ The rates of change in LVMI overall and in patients with baseline LVH were not significantly different from the corresponding values for the full FOS ERT cardiac cohort
Figliozzi 2024 [164]	445	Median [IQR]: 45 [24–58] months MRI	Systematic review and meta-analysis of patients with baseline and follow-up cardiac MRI after ERT Baseline LVM range: 95–270 g LVMI range: 53–176 g/m^2^ Meta-analysis (random-effects model) of LVMI following ERT showed no changes; mean difference: −1 g/m^2^ (95% CI: −6, 3; 6 studies; 290 patients; I^2^ = 81%); *p* < 0.01 Meta-analysis of LVM following ERT: minimal decrease (mean difference: −18 g; 95% CI: −33, −3; 7 studies; 107 patients, I^2^ = 96%); *p* < 0.01
Germain 2013 [166]	Untreated group: 48 Agalsidase beta: 115 (11 included in both panels, because they had at least 2 years of LVM data during each observation period)	Mean [range]: Untreated group, 4.1 [2.1, 12.7] years; Agalsidase beta, 4.8 [1.8, 9.5] years Echocardiography	Fabry Registry study to document the progression of LVH in male patients Patients with LVH (LVM ≥ 225 g) at baseline, *n*/*N* (%) ◦ Untreated group: 29/48 (60%) ◦ Agalsidase beta: 66/115 (57%) At follow-up, LVM slope (g/year) (SEM) by age category ◦ Age 18–29 years - During untreated period (*n* = 15): 9.5 (2.36) - During treatment (*n* = 31): −3.6 (1.62) - Difference in LVM slope: −13.0 (2.72); *p* < 0.0001 Age 30–39 years - During untreated period (*n* = 17): 8.4 (3.55) - During treatment (*n* = 44): 2.8 (2.20) - Difference in LVM slope: −5.6 (4.12); *p* = 0.1760 Age 40–49 years - During untreated period (*n* = 7): 13.4 (6.63) - During treatment (*n* = 23): 3.4 (2.87) - Difference in LVM slope: −10.0 (7.21); *p* = 0.1691 Age ≥ 50 years - During untreated period (*n* = 9): 0.4 (9.41) - During treatment (*n* = 17): 7.7 (4.48) - Difference in LVM slope: 7.3 (10.33); *p* = 0.4843
Golan 2015 [118]	Agalsidase alfa 0.2 mg/kg EOW: 20 Agalsidase alfa 0.2 mg/kg weekly: 19 Agalsidase alfa 0.4 mg/kg weekly: 5	1 year Echocardiography	Phase 3/4 multi-center open-label study in treatment-naive adults Evaluated the efficacy and safety of two agalsidase alfa dosing regimens (0.2 mg/kg EOW and 0.2 mg/kg weekly) in a specific population of adults with FD and LVH (defined as > 50 g/m^2.7^ for males and > 47 g/m^2.7^ for females) Change from baseline in mean LVMI, g/m^2.7^ ◦ 0.2 mg/kg EOW (*n* = 15): 3.2 ◦ 0.2 mg/kg weekly (*n* = 18): 0.5 ◦ 0.4 mg/kg weekly (*n* = 5): −10.3 % change (95% CI) ◦ 0.2 mg/kg EOW (*n* = 15): 3.9 (−3.71, 10.14) ◦ 0.2 mg/kg weekly (*n* = 18): 3.4 (−7.35, 8.32) ◦ 0.4 mg/kg weekly (*n* = 5): −9.3 (−24.9, 4.3) 0.2 mg/kg weekly minus EOW: LSM, −2.20 (95% CI: −12.26, 7.85); unadjusted *p* = 0.6585
Guerard 2018 [119]	Lucerastat + ERT: 10 ERT only: 4	Lucerastat 1000 mg b.i.d. for 12 weeks Echocardiography	Single-center, open-label randomized study of lucerastat in adult patients in Germany Investigated the safety and pharmacodynamics of lucerastat as oral substrate reduction therapy in patients with FD receiving ERT All patients were on ERT for at least 24 months with no change in dose in the last 6 months Mean (SD) baseline LVMI (g/m^2^) ◦ Lucerastat + ERT (*n* = 10): 111.7 (38.4) ◦ ERT only (*n* = 4): 88.6 (17.9) Mean (SD) LVMI (g/m^2^) at week 4 ◦ Lucerastat + ERT (*n* = 10): 104.1 (21.5) ◦ ERT only (*n* = 4): 95.3 (35.7) Mean (SD) LVMI (g/m^2^) at week 8 ◦ Lucerastat + ERT (*n* = 10): 105.9 (24.7) ◦ ERT only (*n* = 4): 90.2 (25.1) Mean (SD) LVMI (g/m^2^) at week 12 ◦ Lucerastat + ERT (*n* = 10): 104.4 (24.3) ◦ ERT only (*n* = 4): 94.3 (24.3) No effect of treatment with lucerastat on LVMI was observed when comparing values obtained at each visit up to 12 weeks with those at baseline
Hughes 2008 [121]	Agalsidase alfa: 7 Placebo: 8	Up to 4 years MRI and Echocardiography	Placebo-controlled study followed by open-label extension for 2 years Mean (SEM) LVMI (g/m^2^) similar at baseline between groups ◦ Agalsidase alfa: 176 (10) ◦ Placebo: 186 (18); *p* = 0.7 Randomized phase ◦ MRI - Agalsidase alfa: mean decrease in LVM of 11.5 g - Placebo: mean increase of 21.8 g (*p* = 0.041 between groups) - Equated to mean increase in LVMI for the placebo group of 12 g/m^2^ compared with a decrease in the treated group of 6.4 g/m^2^ (*p* = 0.02 vs. placebo) ◦ Echocardiography - Placebo: 6.3% increase in LVM 21.5 (20.4) g - Agalsidase alfa: 6.2% decrease −20.4 (27.2) g; *p* > 0.05 ◦ Open-label extension - MRI measurements in the 10 patients who continued to receive agalsidase alfa in the open-label extension phase of the trial demonstrated individual heterogeneity in response - After 2 years of active treatment mean LVM was not significantly changed (*p* = 0.18) Subsequent to the trial, 11 patients continued to receive uninterrupted therapy with agalsidase alfa and have been monitored by annual echocardiography After 4 years, 7 patients demonstrated a reduction in LVM while 4 showed increases compared with their pre-treatment baseline
Jovanovic 2017 * [160] FACETS ATTRACT	FACETS Migalastat or placebo: 67 ATTRACT Migalastat or ERT: 60	FACETS: up to 24 months (plus OLE) ATTRACT: up to 30 months (including OLE) Echocardiography	To assess changes in cardiac parameters with long-term migalastat treatment (150 mg once daily; data from 2 phase 3 RCTs) versus placebo (FACETS) or ERT (ATTRACT) FACETS (migalastat vs. placebo) ◦ After 18 or 24 months of migalastat treatment in FACETS (18 months for patients initially randomized to placebo, 24 months for patients randomized to migalastat), there was a statistically significant mean change from baseline in LVMI −7.7 g/m^2^; 95% CI: −15.4, −0.01; *n* = 27 ◦ Among patients who entered the OLE, further reductions were seen month 30/36: −17.0 g/m^2^; 95% CI: −26.2, −7.9; *n* = 15, including statistically significant changes in patients with LVH at baseline −20.8 g/m^2^; 95% CI: −37.4, −4.1; *n* = 11; 82% (9/11) had reductions and 45% (5/11) had normalizations of LVMI ATTRACT vs. ERT ◦ LVMI was reduced in patients treated with migalastat in the ATTRACT trial ◦ Mean changes from baseline at 18 months - Migalastat (*n* = 31): −6.6 g/m^2^ (95% CI: −11.0, −2.1) - ERT (*n* = 13): −2.0 g/m^2^ (95% CI: −11.0, 7.0); Mean changes from baseline at 30 months - Migalastat (*n* = 30): −3.8 g/m^2^ (95% CI: −8.9, 1.3) Among those with baseline LVH (*n* = 13), LVMI was reduced by −9.0 g/m^2^; 85% (11/13) had reductions and 31% (4/13) had normalizations of LVMI and regression of LVH
Kramer 2014 [161]	ERT: 57 No ERT: 16	Mean (SD): 4.8 (2.4) years MRI	Observational study of adults in a single center in Germany Investigated the impact of myocardial fibrosis in FD; ERT vs. no ERT (type of ERT not defined) Mean (SD) LVM (g/m^2^) ◦ Baseline - ERT (*n* = 57): 92 (33) - *p* < 0.05 vs. no ERT at baseline - No ERT (*n* = 16): 59 (11) At follow-up - ERT (*n* = 56): 90 (30) - *p* < 0.05 vs. no ERT at follow-up - No ERT (*n* = 16): 61 (14)
Lee 2022 [162]	ERT: 267 No ERT: 285	4.1 years NR	Meta-analysis to characterize patients with FD with LVH, with or without ERT, and to assess the effectiveness of ERT in LVH improvement Patients (either ERT-experienced [agalsidase alfa or agalsidase beta]) or ERT-naive) included from 5 clinical cohort studies and 2 RCTs Additional subgroup analysis for 4 years after ERT (*n* = 214) and before ERT (*n* = 228; patients from 3 cohort studies and 1 RCT) LVMI at follow-up ◦ Difference in means of LVMI between the ERT treatment group and the ERT treatment-naive group: −0.149 [95% CI: −0.431, 0.132]; *p* = 0.034 ◦ Difference in means of LVMI between the before ERT group and the 4 years after ERT group: −0.448 [95% CI: −0.787, −0.108]; *p* = 0.037
Madsen 2017 [37]	ERT: 47 No ERT: 19	Median [range]: Patients in ERT 8 [0, 12] years; Patients without ERT 6 [0, 13] years Echocardiography	Progression of cardiac involvement was assessed in a Danish cohort Baseline LVMI (g/m^2^), median [range] ◦ ERT (*n* = 45): 102 [54, 231] ◦ Non-ERT (*n* = 17): 89 [50, 271] LVMI (g/m^2^) at follow-up, median [range] ◦ ERT (*n* = 39): 97 [47, 306] ◦ No ERT (*n* = 14): 104 [55, 328] ◦ ERT with cardiac disease (*n* = 30): 100 [66, 306] ◦ ERT without cardiac disease (*n* = 9): 73 [54, 113] Progression in LVMI (g/m^2^) per year, estimate (SEM) ◦ ERT: −0.8 (0.6) ◦ No ERT: 0.2 (0.7) ◦ ERT with cardiac disease: −1.0 (0.6) ◦ ERT without cardiac disease: −0.5 (1.2)
Mignani 2008 [163]	Dialysis: 17 Transplant: 17	Mean (SD): Dialysis patients: 45.1 (19.8) months; Transplant patients: 48.4 (13.2) months Echocardiography	Cross-sectional survey study of patients in Italy with FD and ESRD receiving RRT (dialysis or renal transplant) and ERT ERT comprised agalsidase beta (*n* = 30) 1 mg/kg or agalsidase alfa (*n* = 3) 0.2 mg/kg Mean baseline LVMI (g/m^2^) ◦ Dialysis patients (*n* = 7): 210.9 ◦ Transplant patients (*n* = 11): 234.6 LVMI percentage change from baseline at year 3 ◦ Dialysis patients (*n* = 7): 19% ◦ Transplant patients (*n* = 11): −6%
Nordin 2019 [156]	Pre-ERT group, 20 Established ERT, 18 No ERT, 18	Mean (SD): 1.1 (0.2) years Median [range]: Pre-ERT group: 1 year; Established ERT: 4.2 [1.4, 12.2] years; No ERT: 1 year MRI	Pre-ERT (initiated ERT, *n* = 20) ◦ Overall, there was no change in mean (SD) LVMI: 93 (42) g/m^2^ at baseline to 92 (40) g/m^2^ (*p* = 0.186) over 1 year after ERT initiation ◦ Among LVH-positive patients, change in mean (SD) LVMI: −2 (3) g/m^2^; *p* = 0.048 ◦ Among LVH-negative patients, no change in mean (SD) LVMI: 0 (4) g/m^2^; *p* = 0.766 Established ERT (advanced stable disease, *n* = 18) ◦ Over 1 year, there was no significant difference in LVMI: 124 (45) g/m^2^ at baseline to 125 (45) g/m^2^ (*p* = 0.070) ◦ LVH-positive vs. LVH-negative: NA (sample size too small) No ERT (early disease, mostly females, *n* = 18) ◦ Over 1 year, there was a small increase in LVMI: 65 (15) g/m^2^ at baseline to 67 (16) g/m^2^ (*p* = 0.005) ◦ LVH-positive vs. LVH-negative: NA (sample size too small) Interactions between all three groups revealed statistically significant difference in LVMI (*p* = 0.009; difference for pre-ERT vs. both established ERT and no ERT)
Pogoda 2023 [165]	Untreated controls: 30 Migalastat-treated: 20 ERT-treated: 48	Up to 81 months Echocardiography	A prospective observational single-center study in Germany The control group was untreated females with FD who were younger than the treated group LVMI at baseline, median [range] g/m^2^ ◦ Untreated females: 82.9 [46.6–155.2] ◦ Migalastat-treated females: 88.2 [48.6–151.3] ◦ ERT-treated females: 114.4 [64.9–297.8] ◦ Migalastat-treated males: 138 [76–220] ◦ ERT-treated males: 130.2 [80.1–396.0] Change per year in LVMI from baseline, median [range] g/m^2^ ◦ Untreated females: 1.44 [−44.6, 45.6] ◦ Migalastat-treated females: 2.57 [−36.8, 10.0] ◦ ERT-treated females: 0.86 [−10.9, 13.9] ◦ Migalastat-treated males: −7.2 [−14.2, 27.9] ◦ ERT-treated males: −1.17 [−15.6, 15.7] Independent of treatment, yearly changes in cardiac parameters including LVMI in females and males were stable, pointing at disease stabilization
Van der Veen 2022 [33]	0.5 mg/kg agalsidase beta: 3 1.0 mg/kg agalsidase beta: 4 Untreated: 23	Median [range]: 10.4 [9.5, 10.7] years MRI and Echocardiography	Compared outcomes in young male patients (median [range]: treated, 24 [14, 26] years; untreated, 22 [13, 27] years) after 10 years of treatment with ERT with those of a group of untreated patients with FD with the same phenotype (classic) and of comparable age 6/7 patients switched to full dose (1 mg/kg EOW) after a median treatment duration of 9 years (range: 8.3, 10) LVM median [range] g/m^2^ at follow-up (baseline values NR) ◦ Based on echocardiography - Treated: 80 [67, 84] - Untreated: 94 [59, 149] - *p* = 0.02 Based on MRI (including papillary muscles) - Treated: 53 [46, 59] - Untreated: 68 [53, 99] - *p* = 0.02 Analyses were repeated after excluding patients with renal involvement; this did not change the outcome Mass measured by echocardiography and MRI correlated well (ϱ = 0.72, *p* = 0.002)
Vedder 2007 [28]	Agalsidase alfa: 18 Agalsidase beta: 16	24 months Echocardiography	Median LVM (SD) [range], g ◦ 12 months: agalsidase beta, 296 (76) [169, 401]; agalsidase alfa, 244 (89) [157, 424] ◦ 24 months: agalsidase beta, 308 (90) [196, 471]; agalsidase alfa, 294 (87) [196, 502] No significant differences between groups

* Congress abstract. B.i.d., twice a day. CI, confidence interval. EOW, every other week. ERT, enzyme replacement therapy. ESRD, end-stage renal disease. FD, Fabry disease. FOS, Fabry Outcome Survey. IQR, interquartile range. LS, least-squares. LSM, least-squares mean. LVH, left ventricular hypertrophy. LVM, left ventricular mass. LVMI, left ventricular mass index. max, maximum. MRI, magnetic resonance imaging. NA, not applicable. NR, not reported. NS, non-significant. OLE, open-label extension. OR, odds ratio. q2w, every 2 weeks. q4w, every 4 weeks. RCT, randomized controlled trial. RRT, renal replacement therapy. SD, standard deviation. SE, standard error. SEM, standard error of the mean.

### 3.3. Cerebrovascular Outcomes

Cerebrovascular disease is a common manifestation of FD [1]. Overall, 24 studies assessing cerebrovascular outcomes were identified, with publications describing single-arm studies of agalsidase alfa (*n* = 6) [21,48,51,60,171,172], agalsidase beta (*n* = 4) [30,71,80,148], mixed or non-specified ERT (*n* = 12) [31,32,34,35,36,76,88,89,90,173,174,175], and other treatments (*n* = 2, both on migalastat) [101,176]. In total, eight publications reported the results of comparator studies [33,37,108,126,163,177,178,179] and one publication described a switch study [131]. An overview of cerebrovascular data from agalsidase alfa and agalsidase beta single-arm studies and the comparator studies is presented in Table 3. Key results from the other types of studies are provided in Supplementary Table S8.

The six agalsidase alfa single-arm studies of cerebrovascular outcomes had follow-up periods ranging from 6 months to 4 years (Table 3A). The largest of these was a long-term study in 677 patients from the FOS registry, of whom 85.4% had no cerebrovascular events over 3 years of treatment with agalsidase alfa [21]. Differences were noted in the frequency of cerebrovascular events between males and females, although these were inconsistent between studies [21,48]. Some smaller studies reported the development of WMLs in patients during treatment, possibly related to aging [51,60,171]. In the four agalsidase beta single-arm studies reporting on cerebrovascular outcomes, follow-up periods ranged from 1 to 20 years (Table 3A). Two studies reported no cerebrovascular events after at least 12 or 24 months of follow-up (*n* = 39 and 9, respectively) [30,148], although the development of new lesions in two asymptomatic male patients was noted in one of these studies [30]. In another study of Fabry Registry data, 10% of patients had a stroke or TIA over a median treatment duration of 6 years (*n* = 40) [80].

Single-arm studies of the effects of mixed or non-specified ERT on cerebrovascular outcomes in adults had median follow-up periods of 3.5 to 9 years, and all found that some patients receiving ERT developed cerebrovascular events (such as stroke or TIA) and/or WMLs (Supplementary Table S8). Two congress publications reported cerebrovascular outcomes in patients receiving migalastat in single-arm studies (one meta-analysis and one observational study) [101,176]. The meta-analysis of data from phase 2 and 3 clinical trials (*n* = 114) found that 7% of patients had cerebrovascular events over a median treatment duration of 4.4 years, with 2% having a first cerebrovascular event during treatment [176]. The observational study (*n* = 97) reported an incidence of 13.2 cerebrovascular events per 1000 patient-years over a median follow-up of 5.1 years [101].

Overall, studies reporting the efficacy of ERT versus placebo or other comparators in relation to cerebrovascular outcomes did not show a significant effect of treatment (Table 3B). One observational study (*n* = 112) investigated cerebrovascular outcomes in patients who either remained on agalsidase beta throughout the whole study, switched from agalsidase beta to alfa or switched from agalsidase beta to alfa and switched back to beta [131] (Supplementary Table S8). Over 24 months, the relative risk for stroke/TIA was lower in the agalsidase beta and switch groups than in the re-switch group [131].

**Table 3:** Overview of cerebrovascular data from (**A**) single-arm agalsidase alfa or agalsidase beta studies and (**B**) comparator studies.

(A)			
Author, Year	*N*	Treatment Duration	Key Results
Agalsidase alfa single-arm studies			
Beck 2018 [21] FOS	Total: 677 Female: 317 (47%) Male: 360 (53%)	Median [IQR]: Overall: 3.0 (1.1, 6.7) years; Female: 2.9 (1.2, 6.3) years Male: 3.2 (1.0, 7.0) years	Observational study from FOS 578 patients (85.4%) (258 female [81.4%] and 320 male [88.9%]) did not experience a CBV event (stroke/TIA/PRIND) during follow-up The Kaplan–Meier point estimate (95% CI) for median age in years at first CBV event was 70.9 (68.1, NE; due to the small number of events) in female patients and NE (64.9, NE) in male patients
Hughes 2011 [48] FOS	Total: 250 Female: 78 (31.2%) Male: 172 (68.8%)	4 years	Observational study using data from FOS Retrospective chart review to determine whether women respond to ERT in a similar way to men Data on CBV events (stroke/TIA/PRIND) were available for 21 women (27%) 4 women (19%) experienced a CBV event at baseline 1 (25%) of these 4 patients was reported to have also had an event between 42 and 54 months after the start of ERT Of the 17 women (81%) who did not experience a CBV event at baseline, 1 (6%) experienced an event between 42 and 54 months after the start of ERT Data on CBV events were available for 23 men (13%) Of the 7 men (30%) in whom a CBV event was reported at baseline, 4 (57%) were noted to have had a subsequent event at the 4-year time point Among the 16 male patients (70%) who were not reported to have had an event at baseline, there were 2 (13%) new reports of a CBV event
Jardim 2006 [171]	8	24 months	Prospective observational open-label study observing central nervous system manifestations during ERT At baseline, 4 patients (50%) showed a widespread pattern of deep WML In this group, WML showed a trend related to age and to the presence of hypoacusia After 12 months of ERT, MRI remained normal in 4 patients (50%), showed the same WML in 2 other patients (25%) and showed worsening in 2 patients (25%) who were the eldest Of the 6 (75%) who completed 24 months on ERT, MRI was stable in 3 (50%); it was normal in 2 patients and showed the same lesions in the other patient. WML worsened in 1 patient (16.7%), fluctuated in 1 patient (16.7%) and disappeared in 1 patient (16.7%), who was the youngest These variations in WML numbers did not correlate with residual enzymatic activity In 2 years, 4 lesions disappeared and 8 appeared Number of WML by patient (age in years at baseline), at baseline/12 months/24 months ◦ Patient 1 (46): 6/9/10 ◦ Patient 2 (36): 6/8/8 ◦ Patient 3 (31): 1/1/1 ◦ Patient 4 (27): 0/0/0 ◦ Patient 5 (27): 0/0 ◦ Patient 6 (24): 2/2/0 ◦ Patient 7 (24): 0/0 ◦ Patient 8 (NR): 0/0/0
Lee 2017 [172] FOS	Total: 37 IVS4: 25 Classic FD: 12	NR	Retrospective analysis comparing central nervous system manifestations in Taiwanese patients with the IVS4 mutation or classic FD mutations Infarcts were observed in similar proportions of patients with IVS4 and classic FD (32.0% and 33.3%, respectively), although there were differences in the site of occurrence Anterior circulation stroke and posterior circulation stroke alone each occurred in 8.0% of patients with IVS4 Anterior and posterior circulation stroke occurred in combination in 16.0% of patients with IVS4 Posterior circulation stroke alone was not observed in patients with classic FD Anterior circulation stroke alone and anterior and posterior circulation stroke combined each occurred in 16.7% of classic patients with FD There was a high prevalence of non-specific signs and symptoms or silent infarcts in both groups (62.5% in IVS4 and 50.0% in classic FD) The pulvinar sign was observed in a greater proportion of patients with classic FD (50.0%) than IVS4 (32.0%) Hemorrhage was noted in MRIs from 16.7% of patients with classic FD but not in patients with IVS4 (*p* = 0.0991) Median (range) basilary artery diameter (mm) was 2.7 (1.4, 4.0) in patients with IVS4 and 3.2 (2.3, 4.7) in patients with classic FD (*p* = 0.0293)
Ries 2006 [51]	24 pediatric patients	24 weeks	Open-label study at 3 tertiary care centers in pediatric patients One CBV accident occurred and was classed as a serious adverse event, considered unrelated to treatment One 16-year-old patient had repeated small-vessel strokes, commencing at 14 years of age, which continued after >18 months on ERT The patient had an intensive work-up that did not uncover a prothrombotic disorder other than FD. However, an unrecognized susceptibility factor for strokes (in addition to FD) was not excluded owing to the uncommonness of recurrent small-vessel strokes at that age
Thofehrn 2009 [60]	9	36 months	Single-center, open-label study in Brazil One patient had a transient CBV ischemic episode at 8 months of ERT and received aspirin and ticlopidine
**Agalsidase beta single-arm studies**			
Cabrera 2017 [30]	39 (switching owing to treatment shortage was allowed)	≥12 months	Long-term prospective and historical analysis of cohort of patients from Argentina Observational cohort study analyzing the effectiveness of long-term follow-up of ERT on renal, cardiac and CBV parameters No patient had a stroke at baseline or during the follow-up Two male patients presented progression in the number of new MRI lesions, all were asymptomatic
Dutra-Clarke 2021 [71]	Total: 26 Adults Female: 13 (50%) Male: 11 (42%) Children Male: 2 (8%)	2–20 years	Retrospective cohort study in the US WMLs were present in 4 adult participants (25%) who had brain imaging (3/10 female patients [30%], 1/6 male patients [17%]) One male patient in this cohort had a history of two CBV accidents, one TIA at 52 years of age and one stroke at 60 years of age Another male patient and one female patient in this cohort had a history of TIAs while on ERT; they were not taking low dose aspirin at the time Adult male patients had higher frequencies of clinical manifestations than adult female patients in all categories, with the exception of neuropathic pain, WMLs and corneal verticillate
Pisani 2005 [148]	9	24 months	Non-randomized, open-label, prospective study evaluating the impact of 2 years of ERT on the clinical expression of FD in patients undergoing maintenance dialysis, and to investigate changes in symptoms and the echocardiographic evolution of FD cardiomyopathy in the context of RRT 2 patients (22%) had CBV symptoms (TIA and stroke) at baseline (before starting ERT) During the entire follow-up period, no patient experienced CBV events, and antihypertensive therapy had to be intensified in only 2 patients
Weidemann 2013 [80]	40	Median [IQR]: 6 (5.1, 7.2) years	Prospective observational study in patients with advanced FD at a single center in Germany 4 patients (10%) developed a stroke during follow-up at ages 41, 46, 54, and 60 years Only 1 of these patients experienced a TIA before the onset of stroke 3 patients (8%) developed new TIA
**(B)**			
**Author, Year**	***N***	**Treatment Duration**	**Key Results**
**Comparator studies**			
Banikazemi 2007 [177]	Agalsidase beta: 51 Placebo: 31	Mean (SD): 18.4 (8.8) months	Randomized, double-blind, multi-center placebo-controlled trial There were no stroke or TIA events in the agalsidase beta group compared with 2 strokes in the placebo group (OR: 0 [95% CI: 0, 3.2]; *p* = 0.14)
Fellgiebel 2014 [178]	Agalsidase beta: 25 Placebo: 16	Mean [range]: 27 [12, 33] months	Post hoc analysis of data from a phase 4 placebo-controlled study The effect of agalsidase beta on WML progression and CBV events were assessed longitudinally At baseline, MRIs showed signs of brain infarction in 20% (5 of 25) of patients in the ERT group and 19% (3/16) of patients in the placebo group At follow-up, MRIs showed that 13% (2/16) of patients in the placebo group and 4% (1 of 25) of patients in the ERT group had developed new infarcts WML burden was present in 63% of patients at baseline and correlated with LVH and previous or recent strokes Mean normalized WML diameter (SD) at baseline, follow-up, follow-up minus baseline: ◦ Agalsidase beta (*n* = 25): 3.5 (5.2), 4.2 (5.6), 0.7 (1.9) ◦ Placebo (*n* = 16): 2.8 (3.0), 3.9 (4.3), 1.0 (2.1) Overall, there were no significant differences in WML burden between the ERT and placebo groups at baseline or at follow-up Patients > 50 years had a significantly higher WML burden at follow-up and significantly higher increases in WML burden over time compared with patients aged ≤ 50 years In a subgroup analysis of patients ≤ 50 years of age, the proportion of patients with a stable or decreased WML load was significantly higher in the agalsidase beta group, (8/18 [44%]) than in the placebo group (4/13 [31%]; *p* = 0.014)
Lenders 2015 [179]	ERT: 187 No ERT: 117	Mean [range]: 6.1 [1, 12]	Observational, retrospective study investigating the risk of thromboembolic events in Germany The additional risk of thromboembolic events with the concurrence of FVL was assessed Among all patients, 14.1% had experienced ≥ 1 stroke or TIA, while 3.3% experienced a deep vein thrombosis or pulmonary embolism ◦ Overall (*n* = 304): 89 thromboembolic events reported, 1.68 events per 100 person-years ◦ ERT (*n* = 8): 15 thromboembolic events, 1.94 events per 100 person-years ◦ No ERT (*n* = 15): 8 thromboembolic events, 3.73 events per 100 person-years Patients with FD not receiving ERT have an ~2.8-fold increased risk of thromboembolic events compared with those receiving ERT The occurrence of thromboembolic events was significantly increased for patients with FVL compared with those without FVL; HR: 5.45; 95% CI: 2.29, 12.99; *p* < 0.0001
Madsen 2017 [37]	Agalsidase alfa or beta (switching allowed): 47 No ERT: 19	Median [range]: ERT: 8 [0, 12] years; No ERT: 6 [0, 13] years	Retrospective cohort study in a Danish cohort At baseline, CBV events (stroke/TIA) occurred in 6 patients (13%) in the ERT group and 1 patient (5%) in the no ERT group At follow-up, CBV events occurred in 7 patients (16%) in the ERT group and 0 patients (0%) in the no ERT group (*p* = 0.37)
Mignani 2008 [163]	Dialysis: 17 Kidney transplant: 17	Mean (SD): Dialysis: 45.1 (19.8) months; Transplant: 48.4 (13.2) months	Cross-sectional survey study in Italian patients with FD and ESRD During ERT, 9 acute cardiac and CBV events occurred in the dialysis group and 3 in the group of allograft recipients (*p* < 0.05); all were considered to unrelated to ERT
Skuban 2017 * [108] FACETS ATTRACT	FACETS Migalastat or placebo: 67 OLE, migalastat: 60	FACETS: 6-month double-blind followed by open-label migalastat from 6 to 12 months (stage 2) plus an additional year ATTRACT: Migalastat or ERT: 53 18 months	Analysis of 2 randomized phase 3 clinical studies ATTRACT: 1 CBV event (6%), a TIA, was reported in the ERT group
Van der Veen 2022 [33]	Treated Agalsidase beta: 3 Agalsidase alfa: 4 Untreated: 23	Median [range]: 10.4 [9.5, 10.7] years	Cross-sectional retrospective study comparing outcomes in patients with classic FD after 10 years of treatment with ERT to a group of untreated patients with the same phenotype of comparable age WMLs were present in 1 treated patient (14%) and 6 untreated patients (26%) (*p* = 0.6) In the untreated group, a lacunar infarction was described in one patient and microbleeds were found in another
Wilcox 2004 [126]	Agalsidase beta: 29 Placebo: 29	30–36 months	Phase 3 randomized, placebo-controlled, double-blind trial 5/58 (8.6%) patients reported CBV events (stroke or TIA) at the time of the report 1 of these patients had an extensive past history of stroke; the other 4 patients, presumably, did not have a prior history of stroke The lack of a placebo group makes interpretation of these data difficult To note, the phase 3 extension study was not designed to assess the effect of ERT on clinical outcomes Data on stroke and CV events were not collected as part of the study but rather as AEs

* Congress abstract. AE, adverse event. CBV, cerebrovascular. CI, confidence interval. CV, cardiovascular. ERT, enzyme replacement therapy. FD, Fabry disease. FOS, Fabry Outcome Survey. FVL, factor V Leiden. HR, hazard ratio. IQR, interquartile range. MRI, magnetic resonance imaging. NE, not estimable. NR, not reported. OLE, open-label extension. OR, odds ratio. PRIND, prolonged reversible ischemic neurologic deficit. RRT, renal replacement therapy. SD, standard deviation. TIA, transient ischemic attack. WML, white matter lesion.

### 3.4. Disease Severity

The MSSI was developed to measure the severity of general, renal, cardiovascular, and neurological signs and symptoms of FD and to monitor the clinical course of disease response to treatment [180]. The MSSI ranges from 0 to 76, with higher scores indicating greater disease severity. Scores are categorized as mild (<20 points), moderate (20–40 points), and severe (>40 points) [180]. Overall, 15 studies were identified that explored the impact of FD treatment on MSSI scores: four single-arm studies of agalsidase alfa [63,180,181,182], one with mixed ERT [28], two with other treatments (migalastat and pegunigalsidase alfa) [107,159], four comparator studies [113,183,184,185,186], and four switch studies [131,133,136,137,187]. No single-arm studies with MSSI data were identified for agalsidase beta. An overview of MSSI data is presented in Table 4.

Three of the four single-arm studies on agalsidase alfa demonstrated significant improvements in MSSI scores after 1–4 years of treatment [63,180,181] (Table 4A). In the fourth study (*n* = 85), 77% of patients had no improvement in MSSI score, and 14% progressed to more severe disease after 1 year of home therapy [182]. A mixed ERT study including agalsidase alfa and agalsidase beta (*n* = 20) found that a severe MSSI score at baseline was associated with treatment failure in male patients after 2 years of follow-up [28]. In an observational study of migalastat (*n* = 16), no significant changes in MSSI scores were observed over 18 months of treatment [159]. An observational study of pegunigalsidase alfa (*n* = 19) showed improvements in each MSSI subscale after 12 months of treatment [107].

Four comparator studies reporting MSSI scores were identified, all of which compared patients receiving unspecified ERT with those not receiving ERT (Table 4B). Two studies found no significant differences in MSSI scores between males in either group, while females on ERT had significantly higher scores [183,185]. One study found a significant improvement in cardiovascular MSSI scores with ERT after 12 months of treatment, although the number of patients was not reported [184,186]. The final study, which used the adapted FOS-MSSI in patients who underwent a kidney transplantation, did not find any differences between the treatment groups [113]. Of the four switch studies, two showed stable MSSI scores after switching from agalsidase beta to agalsidase alfa for 12 or 24 months [137,187], one found similar stable scores after re-switching to agalsidase beta [131], and one showed similar increases in MSSI scores in patients regardless of switching [133].

**Table 4:** Overview of MSSI data in (**A**) single-arm studies and (**B**) comparator and switch studies.

(A)			
Author, Year Study Identifier	*N*	Treatment Duration	Key Results
Agalsidase alfa single-arm studies			
Concolino 2017 [182]	85	Mean [range]: 1.9 years [3 months, 4.5 years]	Multi-center observational study of Italian patients receiving home infusions MSSI total score, mean [range] ◦ Diagnosis (*n* = 73): 16.1 [1, 55] ◦ Starting home therapy (*n* = 75): 18.6 [2, 56] ◦ Last follow-up (*n* = 74): 19.3 [2, 54]
Parini 2008 [181]	30	Median [range]: 2.9 [1.0, 6.2] years	Multi-center observational study in Italy Change from baseline to follow-up in MSSI score, median [range] ◦ Total: −4 [−21, 6]; *p* < 0.0001 ◦ General: −1 [−5, 3]; *p* = 0.0005 ◦ Neurological: −1 [−13, 5]; *p* = 0.0056 ◦ CV and renal MSSI scores did not change from baseline
Whybra 2004 [180]	39	1 year	Single-center study of adults with FD in Germany Decrease in MSSI score from baseline, median [range] ◦ Total: 9 [6, 12]; *p* < 0.001 ◦ General: 2 [1, 3]; *p* < 0.001 ◦ Neurological: 3 [2, 5]; *p* < 0.001 ◦ CV: 2 [0, 4]; *p* < 0.001 ◦ Renal: 0 [0, 2]
Whybra 2009 [63]	40	4 years	Prospective single-center open-label study of female patients in Germany MSSI total score decreased significantly after the first year of treatment and remained significantly reduced through 4 years ◦ Changes in the neurologic and CV subscores were responsible for most of the change in MSSI
**Mixed ERT single-arm studies**			
Vedder 2007 [28]	34	2 years	Randomized open-label study of Dutch and Norwegian patients MSSI score, mean [range] ◦ Males with treatment failure (progression of cardiac, renal or cerebrovascular disease): 44 [17, 59] ◦ Males without treatment failure: 21 [12, 37]; *p* = 0.02 vs. with treatment failure
**Migalastat single-arm studies**			
Camporeale 2023 [159] MAIORA	16	18 months	Prospective, observational single-center study to evaluate cardiac outcomes in Italian patients MSSI (Median [range]) ◦ Total score: baseline: 19.0 [16.0, 23.0]; follow-up: 18.5 [13.5, 24.5]; *p* = 0.31 ◦ General score: baseline: 5.0 [3.0, 6.0]; follow-up: 5.0 [2.0, 6.0]; *p* = 0.31 ◦ Neurological score: baseline: 5.0 [3.0, 7.0]; follow-up: 6.5 [3.5, 8.0]; *p* = 0.85 ◦ CV score: baseline: 9.0 [0.0, 13.0]; follow-up: 9.5 [3.0, 13.0]; *p* = 1.00 ◦ Renal score: baseline: 0.0 [0.0, 2.0]; follow-up: 0.0 [0.0, 0.0]; *p* = 1.00
**Pegunigalsidase alfa single-arm studies**			
Schiffmann 2019 [107] NCT01678898, NCT01769001	19	12 months	Improvements in each subscale of the MSSI were observed during the study ◦ Total MSSI score: baseline: 6.6, follow-up change from baseline: −1.9 ◦ Neurological score: baseline: 7.8, follow-up change from baseline: −2.6 ◦ CV score: baseline: 3.7, follow-up change from baseline: −0.8 ◦ Renal score: baseline: 2.5, follow-up change from baseline: −1.0
**(B)**			
**Author, year** **Study Identifier**		**Treatment** **Groups, *n***	**Key Results**
**Comparator studies**			
Biegstraaten 2010 [183]		ERT: 30 No ERT: 18	Cohort from the Netherlands MSSI total/general/neurological/CV/renal, median [range] ◦ ERT male (*n* = 13) - 27 [10, 47]/5 [2, 11]/6 [3, 13]/9 [1, 15]/0 [0, 12] ◦ Non-ERT male (*n* = 2) - 11 [5, 17]; *p* = 0.23 vs. ERT male/3.5 [2, 5]; *p* = 0.31 vs. ERT male/0.5 [0, 1]; *p* = 0.02 vs. ERT male/7 [3, 11]; *p* = 0.69 vs. ERT male/0; *p* = 0.38 vs. ERT male ◦ ERT female (*n* = 17) - 23 [3, 35]/5 [1, 8]/4 [1, 12]/9 [0, 15]/4 [0, 12] ◦ Non-ERT female (*n* = 16) - 7 [1, 18]; *p* = 0.00 vs. ERT female/2 [0, 5]; *p* = 0.00 vs. ERT female/1.5 [0, 7]; *p* = 0.04 vs. ERT female/1.5 [0, 11]; *p* = 0.07 vs. ERT female/0; *p* = 0.00 vs. ERT female
Chen 2016 [184], Chen 2017 [186]		ERT: 25	MSSI CV score from baseline to 12 months, mean (SD) ◦ ERT baseline: 13.8 (2.8) ◦ ERT follow-up: 12.4 (2.3); *p* < 0.05 vs. baseline
Cybulla 2008 [113] FOS		Kidney transplant + ERT: 20 Kidney transplant + no ERT: 7	FOS-MSSI score, mean (SD) at baseline ◦ ERT: 34.7 (9.2) ◦ No ERT: 31.6 (6.3)
Rosa 2021 [185]		ERT: 22 No ERT: NR	MSSI score, mean (SD) at baseline ◦ ERT men: 29.0 (11.1) ◦ No ERT men: 26.7 (14.9); *p* = 0.78 vs. ERT men ◦ ERT women: 25.8 (8.2) ◦ No ERT women: 10.3 (7.1); *p* = 0.0003 vs. ERT women
**Switch studies**			
Kramer 2018 [131]		Regular dose agalsidase beta: 37 Switch group to agalsidase alfa: 38 Re-switch group to agalsidase beta after 12 months agalsidase alfa: 37	MSSI score, after 12 months of treatment/24 months of treatment, mean (SD) ◦ Regular dose: 25 (13)/27 (12) ◦ Switch: 23 (12)/25 (12) ◦ Re-switch: 21 (10)/22 (11) ◦ *p* < 0.05 switch v re-switch after 12 months
Lenders 2021 [133]		Agalsidase beta regular dose: 17 Switch: 22 (patients treated with agalsidase beta for ≥12 months, then dose-reduced and subsequently switched to agalsidase alfa) Re-switch: 39 (patients treated with agalsidase beta for ≥12 months, then dose-reduced or switched to agalsidase alfa for ≥24 months and then re-switched to agalsidase beta)	All groups showed a significant increase in MSSI scores over the follow-up period of ~80 months ◦ Re-switch group: MSSI increase: 1.0 (95% CI: 0.7, 1.4) per year (*p* < 0.001) ◦ Switch group: MSSI increase: 0.8 (95% CI: 0.2, 1.5) per year(*p* = 0.0189) ◦ Regular agalsidase-beta group: MSSI increase: 1.1 (95% CI: 0.7, 1.4) per year (*p* < 0.001) Comparison between groups: no significant differences in yearly MSSI increase between the three groups (*p* = 0.7483)
Ripeau 2017 [187]		Agalsidase beta switch to agalsidase alfa: 33	MSSI score, mean (SD) ◦ Baseline: 22.1 (1.4) ◦ 24 months: 22.5 (1.4); *p* = NS vs. baseline
Tsuboi 2012 [136], Tsuboi 2014 [137]		Agalsidase beta switch to agalsidase alfa: 11	MSSI score general/neurological/CV/renal/total, mean ◦ Baseline of switch: 4.09/5.18/8.73/4.73/22.73 ◦ After 12 months: 3.91/5.00/8.73/4.73/22.36

CV, cardiovascular. ERT, enzyme replacement therapy. FD, Fabry disease. FOS, Fabry Outcome Survey. IQR, interquartile range. MSSI, Mainz Severity Score Index. NR, not reported. NS, non-significant. SD, standard deviation.

## 4. Discussion

This SLR identified a total of 247 publications reporting findings from 231 studies that reported data on renal, cardiovascular, cerebrovascular, and disease severity (MSSI) outcomes with FD treatments. The majority of these were real-world studies involving adults. As expected, the approved treatments for FD were well represented in the literature, with 187 studies evaluating ERT with agalsidase alfa and/or agalsidase beta, 30 studies examining migalastat, and 11 studies investigating pegunigalsidase alfa. The treatments for which the longest follow-up data were available were agalsidase alfa (up to 20 years [40,43]) and agalsidase beta (up to 10 years [82]). In general, FD treatments stabilized or slowed kidney function decline and cardiac structural changes. However, in the absence of a randomized control group, as was the case for many of the studies, and owing to the variable presentation and slow progression of FD, such findings should be interpreted with caution. There was substantial heterogeneity in the disease severity and clinical characteristics of patients at baseline, both within and between studies, as well as differences in study designs with regard to the length of follow-up, sex balance, age groups, treatment duration, and treatment doses. As a result, cross-study conclusions about the effectiveness of different therapies cannot be accurately made. This is aligned with SLRs by Orsborne et al. (2023) [188], Besekar et al. (2023) [189], and Riccio et al. (2023) [190,191].

FD can significantly impair kidney function, leading to reduced GFR and increased proteinuria [1]. The results of this SLR suggest that ERT with agalsidase alfa or agalsidase beta has renoprotective effects and stabilizes GFR over time. Migalastat also demonstrated stability in renal function in many studies. Cardiac manifestations of FD, including LVH, also contribute significantly to morbidity and mortality [1]. ERT with agalsidase alfa or agalsidase beta or oral chaperone therapy with migalastat stabilized or reduced LVMI, indicating beneficial effects on cardiac structure. A study of 2171 patients from FOS treated with agalsidase alfa for up to 20.8 years (published in April 2025) also demonstrated improved renal and cardiac outcomes compared with external untreated cohorts [192]. Renal outcomes improved regardless of sex or baseline proteinuria, and LVMI stabilized regardless of sex or baseline LVH status. Agalsidase alfa also delayed composite morbidity events and mortality compared with untreated external cohorts [192].

Evidence from studies directly comparing agalsidase alfa and agalsidase beta (limited to two cohort studies and one randomized, controlled, open-label trial) suggests they have similar efficacy with respect to renal and cardiac outcomes. The importance of early treatment initiation was demonstrated, with early initiation of ERT in childhood or young adulthood, before established organ involvement, associated with improved preservation of GFR outcomes and LVMI. In subgroup analyses of ERT studies, patients with impaired kidney function/high renal involvement at baseline experienced a greater decline in eGFR than those with preserved kidney function/low renal involvement at baseline.

Patients with FD are also at increased risk of cerebrovascular events, such as strokes [1]. Studies so far have provided little evidence of a benefit with ERT or migalastat treatment on cerebrovascular outcomes. Comparisons between different ERTs, which do not cross the blood–brain barrier, and migalastat, which may be able to cross the blood–brain barrier [193], require further investigation to establish the optimal treatment strategy for cerebrovascular risk reduction. However, the improvements in cardiovascular function observed with ERT and migalastat treatment may contribute to better cerebrovascular outcomes. After the completion of the literature search for this SLR (17 June 2024), the incidence of stroke was reported in a publication from the Fabry Registry [194]. In agalsidase beta-treated patients, the stroke incidence was 5.55 per 1000 person-years compared with 11.18 per 1000 person-years in matched untreated patients and agalsidase beta-treated patients had a significantly lower risk of stroke compared with untreated patients (HR: 0.36; 95% CI: 0.23, 0.56) [194].

Compared with other outcomes of interest, there was a paucity of research into the effects of FD treatments on MSSI, which incorporates multiple clinical parameters to assess and monitor FD severity as a multisystem disease and helps to stratify patients by clinical phenotype [180]. Agalsidase alfa showed improvements in MSSI scores in several studies (particularly in the general, neurological, and cardiovascular subscores), whereas the effects of agalsidase beta on MSSI scores remain less explored. Studies on the impact of switching treatments showed stability in MSSI scores, suggesting comparable efficacy between agalsidase alfa and beta. Although not evaluated in this SLR, ERT has been shown to improve neuropathic pain and quality of life in patients with FD [195].

Following the completion of the literature search for this SLR (17 June 2024), nine further clinical studies reporting renal and cardiac outcomes in FD have been published. This comprised two studies of agalsidase alfa, two studies of agalsidase beta, four studies of migalastat, and one study of pegunigalsidase alfa [196,197,198,199,200,201,202,203,204]. Overall, these publications support the efficacy of ERT and migalastat (in patients with amenable *GLA* variants) on improving renal and cardiac function and reducing the risk of severe clinical events.

The slow and variable progression of FD mean that careful baseline phenotyping and long-term follow-up is required to demonstrate the clinical benefit–risk profile of treatments and patient adherence with them. Results from long-term studies are awaited for the more recent pegunigalsidase alfa, gene therapy, and substrate reduction therapies. Regarding cardiac outcomes, the major limitation of most of the clinical trials is the limited follow-up time and the inadequacy of surrogate biomarkers for cardiomyopathy [167]. While LVM and LVMI measure the hypertrophic aspect of cardiac deterioration, they do not reflect the risk of arrhythmias and diastolic heart failure, which are the prevalent complications in patients with FD [167]. There were also limited data on the use of additional supportive medications in patients with FD, such as renin–angiotensin blockade, anti-platelet therapies etc. Patients with FD often receive multiple treatments, such as those to manage primary and secondary cardiovascular risks. Use of concomitant medicines are also likely to affect treatment outcomes [15]. Therefore, future studies should address the potential effect of concomitant medications on the efficacy of FD treatments.

Published congress abstracts were included in this SLR to capture all available published data on the outcomes of interest. Unlike journal articles, however, congress publications are typically not peer reviewed and often contain limited study details. Therefore, the results of such studies should be considered with caution. Different studies were identified in this SLR compared with previous SLR reports [188,189,190], owing to differences in methodology and focus. Laboratory measurements, such as serum levels of globotriaosylsphingosine (lyso-Gb3) or the presence of antidrug antibodies, have been suggested to relate to the effectiveness of FD treatments; however, they do not translate directly to clinical outcomes and were therefore not evaluated in this SLR. Although safety outcomes were reported in various identified publications, these outcomes have not been covered here owing to the heterogenous nature of safety reporting in the clinical studies.

A major limitation of this SLR is the small number and heterogeneous phenotypes of patients enrolled in many of the identified clinical trials and observational studies. Although this issue is common in studies of rare diseases, such limited sample sizes may lead to difficulty in generalizing results for larger populations. In addition, follow-up times varied substantially between studies. Long-term follow-up studies with larger patient cohorts, stratified according to clinical phenotype, will be essential for the accurate assessment of the sustained benefits and potential side effects of FD treatments over extended periods. The variability of study designs and the small treatment effects also meant that it was not possible to directly compare different subgroups and studies, so the differential effects for certain patient groups could not be ascertained. Furthermore, studies comparing agalsidase alfa and agalsidase beta had significant limitations. Specifically, studies with a retrospective design can introduce significant biases related to patient selection and data collection, while open-label studies may suffer from biases in data analysis and interpretation. Future studies should address the effects of different FD treatments in different age groups on specific outcomes, with methodology that is reproducible enough to produce robust data for meta-analysis.

In conclusion, after more than 20 years of experience with ERT, data indicate that agalsidase alfa and agalsidase beta demonstrate similar efficacy in stabilizing renal and cardiac outcomes in patients with FD. Early initiation of ERT in childhood or young adulthood before established organ involvement is critical for optimizing treatment outcomes. Treatment with migalastat has also been associated with the stabilization of renal and cardiac outcomes in patients with amenable *GLA* variants. Large cohort studies of current and emerging treatments will require long-term follow-up periods. In addition, better stratification according to clinical phenotype and prognosis will be crucial for addressing evidence gaps and providing clinicians and policymakers with comprehensive and reliable data to offer informed treatment options and share treatment decisions with their patients.

## Data Availability

The data included in this report are from the published literature.

## Supplementary Materials

The following supporting information can be downloaded at: https://www.mdpi.com/article/10.3390/jcm14145131/s1, Figure S1: Study locations; Table S1: EMBASE, MEDLINE and Cochrane search strategies; Table S2: Eligibility search criteria; Table S3: Risk of bias assessment for included studies; Table S4: Overview of GFR data from (A) mixed or non-specified ERT single-arm studies and single-arm studies for other treatments and (B) switch studies; Table S5: Overview of proteinuria data from (A) single-arm studies and (B) comparator and switch studies; Table S6: Overview of LVMI data from (A) mixed or non-specified ERT single-arm studies and single-arm studies for other treatments and (B) switch studies; Table S7: Overview of data for other structural cardiovascular parameters from (A) single-arm studies, (B) comparator and (C) switch studies; Table S8: Overview of cerebrovascular data from (A) mixed or non-specified ERT single-arm studies and single-arm studies for other treatments and (B) switch studies.
