# Supplementary material for: Clinical Efficacy and Real-World Effectiveness of Fabry Disease Treatments: A Systematic Literature Review
Source: J Clin Med. 2025 Jul 18;14(14):5131. doi: 10.3390/jcm14145131 (PMC12295217; doi:10.3390/jcm14145131)
Supplement: Supplementary file 1 [file jcm-14-05131-s001.zip › jcm-3689402-supplementary.pdf]

SUPPLEMENTARY INFORMATION

Supplementary Table S1. EMBASE, MEDLINE and Cochrane search

**Embase**		
**#**	**Searches**	**Type**
1	Fabry Disease/	Fabry disease
2	fabry*.ti,ot,ab,hw,kw.	
3	(angiokeratoma adj3 diffusum).ti,ot,ab,kw,hw.	
4	diffuse angiokeratoma*.ti,ot,ab,kw,hw.	
5	(galactosidase adj3 deficiency).ti,ot,ab,kw,hw.	
6	exp alpha-Galactosidase/	
7	alpha galactosidase.ti,ot,ab,kw,hw.	
8	(GLA adj3 deficiency).ti,ot,ab,kw,hw.	
9	or/1-8	
10	exp agalsidase alfa/	Interventions
11	(replagal or agalsidase alfa).mp.	
12	exp agalsidase beta/	
13	fabrazyme.mp.	
14	exp enzyme replacement/	
15	((enzyme replacement adj (therap* or treatment*)) or ERT).mp.	
16	(migalastat or (chaperone adj3 therapy) or galafold or pegunigalsidase or PRX-102).tw.	
17	(lucerasat or venglustat of st-920 or AVR?RD?01 or DRX005B or FLT190 or gene therapy or substrate reduction therapy or best supportive care or standard of care or SoC).tw.	
18	or/10-17	
19	clinical trial/ or randomi*ed control* trial/ or controlled clinical trial/ or multicenter study/ or phase 3 clinical trial/ or phase 4 clinical trial/ or single blind procedure/ or double blind procedure/ or placebo/	RCTs (SIGN)
20	exp RANDOMIZATION/	
21	PLACEBO/	
22	(randomi?ed control* trial$ or rct or (random$ adj2 allocat$) or single blind$ or double blind$ or ((treble or triple) adj blind$) or placebo$).tw.	
23	Prospective Study/	
24	or/19-23	
25	(editorial or letter or note or case report).tw,pt. or case study/	
26	24 not 25	
27	clinical study/ or case control study/ or family study/ or longitudinal study/ or retrospective study/ or prospective study/	Observational studies (SIGN)
28	(Cohort adj (study or studies)).mp.	
29	(Case control adj (study or studies)).tw.	
30	(follow up adj (study or studies)).tw.	
31	(observational adj (study or studies)).tw.	
32	(epidemiologic$ adj (study or studies)).tw.	
33	(cross sectional adj (study or studies)).tw.	
34	or/27-33	
35	(claims or database or registry or survey or questionnaire or register or medical record or (chart adj3 review) or database or (real adj world) or RWE or RWD).tw.	
36	26 or 34 or 35	Boolean combinations
37	9 and 18 and 36	
38	limit 37 to english language	
39	limit 38 to (books or chapter or "conference review" or editorial or erratum or letter or "review")	
40	38 not 39	
41	limit 40 to dd=20221001-20240610	Search update
**MEDLINE**		
**#**	**Searches**	**Type**
1	Fabry Disease/	Fabry’s disease

2	fabry*.ti,ot,ab,hw,kw.	
3	(angiokeratoma adj3 diffusum).ti,ot,ab,kw,hw.	
4	diffuse angiokeratoma*.ti,ot,ab,kw,hw.	
5	(galactosidase adj3 deficiency).ti,ot,ab,kw,hw.	
6	exp alpha-Galactosidase/	
7	alpha galactosidase.ti,ot,ab,kw,hw.	
8	(GLA adj3 deficiency).ti,ot,ab,kw,hw.	
9	or/1-8	
10	(replagal or agalsidase alfa).mp.	Interventions
11	fabrazyme.mp.	
12	((enzyme replacement adj (therap* or treatment*)) or ERT).mp.	
13	(migalastat or (chaperone adj3 therapy) or galafold or pegunigalsidase or PRX-102).tw.	
14	(lucerasat or venglustat of st-920 or AVR?RD?01 or DRX005B or FLT190 or gene therapy or substrate reduction therapy or best supportive care or standard of care or SoC).tw.	
15	or/10-14	
16	Randomized Controlled Trials as Topic/ or randomized controlled trial/ or Random Allocation/ or Double Blind Method/ or Single Blind Method/ or clinical trial/	RCTs (SIGN)
17	clinical trial, phase i.pt.	
18	clinical trial, phase ii.pt.	
19	clinical trial, phase iii.pt.	
20	clinical trial, phase iv.pt.	
21	(control* clinical trial$ or multicent* stud* or clinical trial$).pt. or exp Clinical Trials as topic/	
22	((clinical adj trial$) or ((singl$ or doubl$ or treb$ or tripl$) adj (blind$3 or mask$3)) or placebo$ or (random* adj2 allocat*)).tw. or PLACEBOS/	
23	or/16-22	
24	case report.tw. or letter/ or historical article/	
25	23 not 24	
26	Epidemiologic studies/ or exp case control studies/ or exp cohort studies/ or Cross-sectional studies/	Observational studies (SIGN)
27	(case control or (cohort adj (study or studies)) or cohort analy$ or (Follow up adj (study or studies)) or (observational adj (study or studies)) or longitudinal or retrospective or cross?sectional).tw.	
28	26 or 27	
29	(claims or database or registry or survey or questionnaire or register or medical record or (chart adj3 review) or database or (real adj world) or RWE or RWD).tw.	
30	9 and 15 and (25 or 28 or 29)	Boolean combinations
31	limit 30 to english language	
32	limit 31 to (editorial or letter)	
33	31 not 32	
34	limit 33 to dt=20221001-20240610	Search update
35	limit 33 to rd=20221001-20240610	
36	34 or 35	
**Cochrane**		
**#**	**Searches**	**Type**
1	Fabry Disease/	Fabry disease
2	fabry*.ti,ot,ab,hw,kw.	
3	(galactosidase adj3 deficiency).ti,ot,ab,kw,hw.	
4	exp alpha-Galactosidase/	
5	alpha galactosidase.ti,ot,ab,kw,hw.	
6	or/1-5	
7	(replagal or agalsidase alfa).mp.	Interventions
8	fabrazyme.mp.	
9	((enzyme replacement adj (therap* or treatment*)) or ERT).mp.	
10	(migalastat or (chaperone adj3 therapy) or galafold or pegunigalsidase or PRX-102).tw.	

11	(lucerasat or venglustat of st-920 or AVR?RD?01 or DRX005B or FLT190 or gene therapy or substrate reduction therapy or best supportive care or standard of care or SoC).tw.	
12	or/7-11	
13	Randomized Controlled Trials as Topic/ or randomized controlled trial/ or Random Allocation/ or Double Blind Method/ or Single Blind Method/ or clinical trial/	RCTs (SIGN)
14	(control* clinical trial$ or multicent* stud* or clinical trial$).pt. or exp Clinical Trials as topic/	
15	((clinical adj trial$) or ((singl$ or doubl$ or treb$ or tripl$) adj (blind$3 or mask$3)) or placebo$ or (random* adj2 allocat*)).tw. or PLACEBOS/	
16	or/13-15	
17	case report.tw. or letter/ or historical article/	
18	16 not 17	
19	Epidemiologic studies/ or exp case control studies/ or exp cohort studies/ or Cross-sectional studies/	Observational studies (SIGN)
20	(case control or (cohort adj (study or studies)) or cohort analy$ or (Follow up adj (study or studies)) or (observational adj (study or studies)) or longitudinal or retrospective or cross?sectional).tw.	
21	19 or 20	
22	(claims or database or registry or survey or questionnaire or register or medical record or (chart adj3 review) or database or (real adj world) or RWE or RWD).tw.	
23	6 and 12 and (18 or 21 or 22)	Boolean combinations
24	limit 30 to english language [Limit not valid in DARE,CLCMR,CDSR,ACP Journal Club,CCA; records were retained]	
25	limit 31 to (editorial or letter) [Limit not valid in DARE,CLEED,CLHTA,CLCMR,CDSR,CCA; records were retained]	
26	24 not 25	
27	limit 26 to yr="2022 -Current"	Search update

Supplementary Table S2. Eligibility search criteria

Category	Inclusion criteria	Exclusion criteria
Population	• Patients with Fabry disease<br>○ Age: Any<br>○ Sex: Any<br>○ Race: Any<br>○ Ethnicity: Any	• Patients without Fabry disease
Interventions	• ERT (i.e. agalsidase alfa, agalsidase beta, mixed or non-specified ERT)<br>• Chaperone therapy (i.e. migalastat)<br>• Next-generation ERT (i.e. pegunigalsidase alfa)<br>• Gene therapy (e.g. FLT190, ST-920)<br>• Substrate reduction therapy (e.g. lucerastat, venglustat)<br>• Any other investigational therapy in phase 2/3 (e.g. AVR-RD-01, DRX005B)	• Not applicable
Comparator	• Any of the included interventions<br>• Placebo, best supportive care, observation	
Outcomes	• Clinical efficacy, real-world effectiveness, safety	• Health-related quality of life outcomes, economic burden, other outcomes
Study design	• Observational studies (including case series, retrospective, cross-sectional, prospective, case-control, registry, cohort, database studies)<br>• Clinical studies, any phase (randomized controlled trials, single-arm intervention studies)<br>• Meta-analyses	• Case studies and case reports
Country	• Not restricted by country or region	
Language	• Studies published in English language	
Dates	• Not restricted by publication date[a]	

[a]Electronic database searches were run on 28 November 2022 and then repeated using the same criteria on 17 June 2024
ERT, enzyme replacement therapy; FD, Fabry disease

Supplementary Table S3. Risk of bias assessment for included studies

	RoB2 tool			ROBINS-I tool				JBI checklist		
**Studies, n (%)**	**Low**	**Some concerns**	**High**	**Low**	**Moderate**	**Serious**	**Critical**	**Met all 11 criteria**	**Met > 5 criteria**	**Met ≤ 5 criteria**
Randomized clinical trials										
Articles	6 (38)	8 (50)	2 (13)	–	–	–	–	–	–	–
Congress publications	0	2 (50)	2 (50)	–	–	–	–	–	–	–
Non-randomized clinical trials										
Articles	–	–	–	8 (22)	20 (56)	8 (22)	0	–	–	–
Congress publications	–	–	–	3 (20)	3 (20)	6 (40)	3 (20)	–	–	–
Observational studies										
Articles	–	–	–	51 (40)	46 (36)	26 (20)	5 (4)	–	–	–
Congress publications	–	–	–	2 (8)	8 (31)	15 (58)	1 (4)	–	–	–
Meta-analyses										
Articles	–	–	–	–	–	–	–	1 (33)	2 (67)	0
Congress publications	–	–	–	–	–	–	–	1 (33)	1 (33)	1 (33)

JBI, Joanna Biggs Institute; ROBINS-I, Risk Of Bias In Non-randomized Studies – of Interventions.

Supplementary Table S4

Overview of GFR data from (A) mixed or non-specified ERT single-arm studies and single-arm studies for other treatments and (B) switch studies.

A

**Author, year**	***N***	**Treatment duration**<br>***Treatment detail***	**Key results**
**Mixed or non-specified ERT single-arm studies**			
Arends 2017a [1]	293	Median [range] : 6.8 [0.8, 15.4] years<br><br>*100 patients changed in dose or preparation during follow-up. In 78 patients the dose of agalsidase beta was temporarily lowered or switched to agalsidase alfa, as a result of a shortage of agalsidase beta*	• Effect of age, sex and phenotype on eGFR slope, HR (95% CI); p value<br>○ Age (per 10 years): 0 (−0.3, 0.2); p < 0.01<br>○ Classic men (n = 121): −2.7 (−3.1, −2.3); p < 0.001<br>○ Non-classic men (n = 42): −1.8 (−2.5, −1.1); p < 0.001<br>○ Classical women : −1.3 (−1.7, −0.8); p < 0.001<br>○ Non-classical women (n = 48): −1.4 (−2.0, −0.8); p < 0.001<br>○ Men with classical FD had a more severe decline in renal function compared with the other subgroups<br>• With every 10 mL/min/1.73 m^2 lower eGFR at baseline, there was a 19% increased risk of developing a clinical event (HR: 1.19; p < 0.001)<br>• A lower eGFR at baseline was associated with an increased risk of developing a first renal event (HR per −10 mL/min/1.73 m^2: 5.57; p = 0.02)<br>• Proteinuria (> 0.5 g/day or equivalent) at baseline did not result in a higher clinical event rate, but in patients with proteinuria an additional decline in eGFR of −0.9 mL/min/1.73 m^2 per year (p = 0.005) was found, which was more prominent in patients with severe proteinuria (> 1 g/day or equivalent (β: −1.5 mL/min/1.73 m^2; p < 0.001)
Goicoechea 2021 [2]	69	Median [range]: 60 [24, 120] months	• Mean (SD) eGFR at baseline (mL/min/1.73 m^2)<br>○ Males (n = 42): 61.2 ±(36.1)<br>○ Females (n = 27): 84.4 (27.1)<br>• Mean (SD) eGFR (mL/min/1.73 m^2) after a median of 5 years on ERT<br>○ Males: 64.9 (33.1)<br>○ Females: 92.1 (39.4)<br>• Mean (SD) eGFR (mL/min/1.73 m^2) difference between patients who did or did not have clinical events<br>○ Clinical events (n = 21): 55.1 (31.9)<br>○ No clinical events (n = 48): 77.2 (33.8)
Lin 2013 [3]	36	Median [range]: 16 [6, 39] months	• Mean (SD) eGFR (mL/min/1.73 m^2)<br>○ Baseline (*n* = 35): 87.9 (22)<br>○ After ERT: 89.5 (24.6); NS
Lin 2014b [4]	9	Agalsidase beta: range, 0.7, 88.6 months<br>Agalsidase alfa: ≥ 1 year; range (*n* = 8), 15, 112 months	• eGFR (mL/min/1.73m^2), range<br>○ Baseline: 31.5, 133.8<br>○ After ERT: 31.6, 141.2
Madsen 2019 [5]	52	Median [range]: 335 [23, 636] weeks	• Mean (SD) eGFR slope (mL/min/1.73 m^2/year) by reported age group at baseline<br>○ 0–29 years (*n* = 21): −1.5 (0.6)<br>○ 30–49 years (*n* = 20): −0.6 (0.5)<br>○ ≥ 50 years (*n* = 11): −0.6 (0.5)

Author, year	*N*	Treatment duration<br>*Treatment detail*	Key results
			○ Combined (*n* = 52): −0.8 (0.3)<br>• Mean (SD) measured GFR slope (mL/min/1.73 m^2/year) by reported age group<br>○ 0–29 years (*n* = 21): −0.1 (0.3); vs ≥ 50 years *p* < 0.001; vs 30–49 years *p* = 0.045<br>○ 30–49 years (*n* = 20): −1.0 (0.3)<br>○ ≥ 50 years (*n* = 11): −2.0 (0.4)<br>○ Combined (*n* = 52): −0.9 (0.2)<br>• Males (*n* = 20): mean (SD) measured GFR slope: −0.7 (0.4) mL/min/1.73 m^2/year<br>• Females (*n* = 32): mean (SD) measured GFR slope: −1.0 (0.3) mL/min/1.73 m^2/year<br>• Males (*n* = 20): eGFR slope:−1.8 (0.5) mL/min/1.73 m^2/year<br>• Females (*n* = 32): eGFR slope:−0.01 (0.4) mL/min/1.73 m^2/year<br>• Mean (SD) eGFR slope (mL/min/1.73 m^2/year) by reported UACR group<br>○ < 30 mg/g (*n* = 21): −0.8 (0.4)<br>○ 30−299 mg/g (*n* = 18): −0.3 (0.5)<br>○ ≥ 300 mg/g (*n* = 9): −2.1 (0.7); vs 30−299 mg/g; *p* = 0.038<br>• Mean (SD) measured GFR slope (mL/min/1.73 m^2/year) by reported UACR group<br>○ < 30 mg/g (*n* = 21): −0.4 (0.3)<br>○ 30−299 mg/g (*n* = 18): −0.8 (0.3)<br>○ ≥ 300 mg/g (*n* = 9): −3.1 (0.5); vs < 30 mg/g *p* < 0.001; vs 30−299 mg/g; *p* = 0.001<br>• Mean (SD) measured GFR slope (mL/min/1.73 m^2/year) in patients with normal GFR at baseline, by reported UACR grou*p*<br>○ < 30 mg/g (*n* = 20): −0.4 (0.3)<br>○ 30−299 mg/g (*n* = 16): −0.8 (0.4)<br>○ ≥ 300 mg/g (*n* = 5): −2.7 (0.6); vs < 30 mg/g *p* = 0.002; vs 30−299 mg/g; *p* = 0.011<br>• Mean (SD) measured GFR slope during ERT (mL/min/1.73 m^2/year) in patients with normal GFR at baseline, by reported by age<br>○ 0–29 years (*n* = 19): −0.1 (0.3); vs ≥ 50 years; *p* = 0.001<br>○ 30–49 years (*n* = 17): −0.8 (0.3); vs 30–49 years; *p* = 0.036<br>○ ≥ 50 years (*n* = 9): −1.9 (0.4)<br>○ Combined (*n* = 45): −0.7 (0.2)<br>• Slope of annualized progression of renal function during ERT by UACR at baseline (deviations from mean or renal healthy patients in SD/year)<br>○ < 30 mg/g (*n* = 21): 0.00 (0.02); *p* = 0.992 vs renal healthy<br>○ 30–299 mg/g (*n* = 18): −0.02 (0.02); *p* = 0.317 vs renal healthy; *p* = 0.435 vs < 30 mg/g<br>○ ≥ 300 mg/g (*n* = 9): −0.15 (0.03); *p* < 0.001 vs renal healthy; *p* < 0.001 vs < 30 mg/g; *p* = 0.002 vs 30–299 mg/g
Miwa 2019 [6], Miwa 2018* [7]	30	Mean (SD): 7.2 (4.6) years	• Mean (SD) eGFR (mL/min1.73m^2)<br>○ Baseline cohort (*n* = 30): 86.0 (31.2)<br>○ Follow-up cohort (*n* = 21): 81.3 (35.4), *p* = 0.36<br>○ Males (*n* = 14): 92.3 (38.7) at baseline<br>○ Female (*n* = 16): 80.6 (23) at baseline, *p* = 0.33<br>• Mean (SD) annual change in eGFR (mL/min1.73 m^2)<br>○ Follow-up cohort: −3.86 (5.42)<br>○ Males: −4.91 (6.50)<br>○ Females: −2.96 (4.34)
Rombach 2013 [8]	57	ERT cohort, median [range] follow-up<br>• Adults: 5.5 [0.51, 10.0] years	• Mean (SD) baseline eGFR, mL/min/1.73 m^2<br>○ Males: 88.5 (40.6)<br>○ Females: 86.6 (31.3)<br>○ Adolescents: 150.4 (42.8)

Author, year	*N*	**Treatment duration**<br>***Treatment detail***	**Key results**
		• Adolescents: 4.7 [2.0, 6.8] years	• Analysis of function, eGFR mean (SE) change, mL/min/1.73 m^2 per year<br>○ Males: −3.4 (0.2); *p* < 0.001<br>○ Females: −0.8 (0.3); *p* = 0.001<br>○ Adolescents: −7.3 (1.0); *p* < 0.001<br>• By CKD stage, eGFR mean (SE) change, mL/min/1.73 m^2 per year<br>○ Males (CKD stages 1/2/3/4/5): −4.5 (0.4) / −2.1 (0.3) / −4.1 (0.2) / −2.0 (0.2) / NR; all *p* < 0.001<br>○ Females (CKD stages 1/2/3/4/5): −1.3 (0.7); *p* = 0.07 / −0.3 (0.3); *p* = 0.25 / −1.4 (0.5); *p* = 0.015 / −3.5 (0.3); *p* NR/ NR
Sirrs 2014 [9]	362	• Median Cohort 1a (previously received ERT): 64 months<br>• Cohort 1b (started ERT): 59 months<br>• Cohort 1c (no ERT): 52 months	• Cohort 1a, mean (SD) MDRD GFR (mL/min/1.73 m^2) at baseline<br>○ Combined: 87 (40.2)<br>○ Females: 78.8 (25.4)<br>○ Males: 90.2 (44.6)<br>• Cohort 1b, mean (SD) MDRD GFR (mL/min/1.73 m^2) at baseline<br>○ Combined: 79.1 (25.4)<br>○ Females: 79.5 (23.2)<br>○ Males: 78.3 (29.7)<br>• Cohort 1c, mean (SD) MDRD GFR (mL/min/1.73 m^2) at baseline<br>○ Combined: 96.1 (21.6)<br>○ Females: 95.5 (20.5)<br>○ Males: 100.1 (27.3)<br>• Cohort 1a, mean (SE) proteinuria at entry ≤ 0.5 g/day subgroup, annual change from baseline in eGFR (mL/min/1.73 m^2/year)<br>○ Combined: −1.87 (0.88)<br>○ Females: −0.43 (0.86)<br>○ Males: −2.45 (1.23)<br>• Cohort 1a, mean (SE) proteinuria at entry > 0.5 g/day subgroup, annual change from baseline in eGFR (mL/min/1.73 m^2/year)<br>○ Combined: −3.73 (1.04)<br>○ Females: −1.11 (3.68)<br>○ Males: −4.49 (0.57)<br>• Cohort 1b, mean (SE) proteinuria at entry ≤ 0.5 g/day subgroup, annual change from baseline in eGFR (mL/min/1.73 m^2/year)<br>○ Combined: −0.25 (0.67)<br>○ Females: 0.21 (0.65)<br>○ Males: −1.97 (2.5)<br>• Cohort 1b, mean (SE) proteinuria at entry > 0.5 g/day subgroup, annual change from baseline in eGFR (mL/min/1.73 m^2/year)<br>○ Combined: −1.93 (0.68)<br>○ Females: −0.1 (0.6)<br>○ Males: −3.74 (1.07)
Skrunes 2017 [10]	20	Median [range]<br>• Lower fixed dose group: 7.8 [4.9, 9.9] years<br>• Higher dose group: 10.5 [4.8–13.8] years	• Mean (95% CI) eGFR, mL/min/1.73 m^2 at baseline<br>○ Total (n = 20): 105 (95, 115)<br>○ Lower dose (n = 10): 98 (85, 111)<br>○ Higher dose (n = 10): 112 (95, 129)<br>• Mean (95% CI) measured GFR, mL/min/1.73 m^2 at baseline<br>○ Total (n = 20): 98 (88, 107)<br>○ Lower dose (n = 10): 96 (80, 111)

Author, year	N	Treatment duration<br>*Treatment detail*	Key results
		• Total cohort: 9.4 [4.8, 13.8] years<br><br>*Variable doses of agalsidase alfa or beta. Some patients switched from agalsidase alfa to beta or vice versa*	○ Higher dose (*n* = 10): 100 (85, 114)<br>• Mean (95% CI) change in measured GFR, mL/min per 1.73 m^2<br>○ Total (n = 20): −5 (−11, 0)<br>○ Lower dose (n = 10): −3 (−12, 6)<br>○ Higher dose (n = 9) −9 (−15, 0)<br>○ *p* = 0.34 (Patient 1 was excluded from analyses due to *de novo* glomerulonephritis during follow-up).
Talbot 2015 [11]	25	Up to 10 years	• Assessed the impact of ESRD on renal outcomes in male patients in Australia<br>• Renal function in patients without ESRD was stable over the 10 years<br>• Mean (SD) GFR, mL/min/1.73 m2<br>○ Baseline (n = 15): 86.9 (16.6)<br>○ Year 10 (n = 12): 82.2 (25.4); *p* = 0.57<br>○ Patients with ESRD: NR
Wyatt 2012 [12], Anderson 2014 [13]	311	Mean, median (SD) [range]<br>• Adult males (*n* = 120): 3.74, 3.51 (2.66) [0, 9.72] years<br>• Adult females (*n* = 169): 3.34, 3.55 (2.25) [0, 8.77] years<br>• Children males (*n* = 11): 1.6, 1.2 (1.8) [0, 4.2] years<br>• Children females (n = 11): 2.3, 2.4 (0.7) [1.7, 3.0] years	• Estimate of change in eGFR mL/min/1.73 m^2 (in patients with no proteinuria pre-treatment) by time on ERT (95% CI), mean at start of ERT; < 12 months; 12–36 months; > 36 months; *p* value<br>○ All adults (*n* = 178): 84.7; 2.20 (−0.90, 5.31); 3.58 (0.78, 5.93); 6.01 (3.28, 8.73); *p* < 0.001<br>○ Male adults (*n* = 77): 86.5; −0.10 (−4.74, 4.53); 5.44 (1.59, 9.28); 4.74 (0.74, 8.75); *p* = 0.01<br>○ Female adults (*n* = 101): 83.6; 4.04 (0.03, 8.11); 0.37 (−3.0 ,3.75); 7.98 (4.34, 11.6); *p* < 0.001<br>○ Children (*n* = 22): 109.5; −2.35 (−3.26, 0.55); 15.8 (2.11, 29.4); 5.40 (−21.6, 32.4); *p* = 0.12<br>• Estimate of change in eGFR mL/min/1.73 m^2 (in patients with proteinuria pre-treatment) by time on ERT (95% CI), mean at start of ERT; < 12 months; 12–36 months; > 36 months; *p* value<br>○ All adults (*n* = 94): 82.8; −1.05 (−5.53, 3.43); 0.16 (−3.43, 3.75); 1.04 (−2.89, 4.97); *p* = 0.85<br>○ Male adults (*n* = 33): 75.2; −6.28 (−14.3, 1.72); −3.61 (−9.38, 2.16); −3.89 (−10.8, 3.08); *p* = 0.40<br>○ Female adults (*n* = 61): 87.8; 0.98 (−4.28, 6.25); 1.99 (−2.51, 6.49); 3.58 (−1.07, 8.23); *p* = 0.49<br>○ Children (*n* = 5): 81.0; 14.0 (−30.9, 58.9); 21.0 (−4.02, 46.1); 7.58 (−18.7, 33.9); *p* = 0.36<br>• There was a significant decline in eGFR levels in adults with age; −0.95 mL/min/1.73 m^2<br>○ After adjusting for age, there was a statistically significant association between increased eGFR and time on ERT (*p* = 0.002) when time on ERT was categorized as 'not treated', < 12 months, 12–36 months and > 36 months<br>• There was a significant decline in eGFR levels in male and female adults with age, the slope of decline was greater for males than for females (−1.40 vs −0.69 mL/min/1.73 m^2)<br>○ After adjusting for age, there was no statistically significant association between increased eGFR and time on ERT in male patients (*p* = 0.11) when time on ERT was categorized as 'not treated', < 12 months, 12–36 months and > 36 months<br>○ However, in female patients there was a significant increase in the slope of the eGFR with time on ERT (*p* < 0.001)<br>• When time on ERT was treated as a continuous variable, no statistically significant association between eGFR in adult males and time on ERT (slope = 0.36 mL/min/m^2/year, edf = 1; *p* = 0.21)<br>○ However, for adult female patients, there was a statistically significant linear increase in eGFR with time on ERT after adjusting for age (slope = 1.15 mL/min/m^2/year, edf = 1; *p* < 0.001)<br>• In children (*n* = 17), a statistically significant decrease in the slope of eGFR was seen with age (–2.24 mL/min/1.73 m^2; 95% CI: –4.27, –0.22; *p* = 0.04)<br>○ After adjusting for age, there was an increase in the slope of eGFR with time on ERT, but this did not reach statistical significance (*p* = 0.06)<br>• When time on ERT was treated as a continuous variable, there was no statistically significant evidence of an association between age-adjusted eGFR levels and time on ERT (edf = 2.2; *p* = 0.14)

Author, year	*N*	Treatment duration<br>*Treatment detail*	Key results
			○ There was a significant decline in eGFR levels in girls with age (–2.68 mL/min/1.73 m^2/year; 95% CI: –5.17, –0.19; *p* = 0.04), whereas no significant association between age and eGFR was observed for boys (*p* = 0.32)<br>○ After adjusting for age, there was no statistically significant association between eGFR and time on ERT in either boys (*p* = 0.21) or girls (*p* = 0.15)
**Migalastat single-arm studies**			
Bichet 2021 [14]	78	Median [range], years: ERT-naive: 7.0 [2.0–8.6]; ERT-experienced: 5.1 [2.1–7.2]	• Mean (SD) annualized change from baseline in eGFR<br>○ ERT-naive: −1.6 (3.1) mL/min/1.73 m²<br>○ ERT-experienced: −1.6 (3.6) mL/min/1.73 m²
Hughes 2023a* [15]	125	Mean: 3.9 years	• Median (IQR) eGFR CKD-EPI annualized rate of change for all patients: −1.2 (−3.7, 1.0) mL/min/1.73 m^2/year<br>• Median (IQR) rate of change by gender<br>○ Males: −1.4 (−3.7, 1.1) mL/min/1.73 m^2/year<br>○ Females: −1.1 (−3.4; 1.0) mL/min/1.73 m^2/year<br>• Statistical analyses not reported
Germain 2016 [16], Germain 2019 [17]	50	6 months plus 6- to 12-month open-label extension plus an additional year	• Baseline measured GFR, mean (SD), mL/min/1.73 m^2<br>○ Total (*n* = 48): 81.3 (27.5)<br>○ Double-blind migalastat → open-label migalastat (*n* = 27): 80.0 (30.9)<br>○ Double-blind placebo → open-label migalastat (*n* = 21): 83.1 (22.8)<br>• Baseline eGFR, mean, mL/min/1.73 m^2<br>○ Total (*n* = 50): 92.7 (23.0)<br>○ Double-blind migalastat → open-label migalastat (*n* = 28): 94.4 (27.0)<br>○ Double-blind placebo → open-label migalastat (*n* = 22): 90.6 (17.1)<br>• In the modified ITT population with suitable mutations, there were no significant between-group differences in the change in eGFR or measured GFR from baseline to month 6<br>• In patients followed for up to 24 months of migalastat, the mean (SE) annualized changes from baseline in estimated GFR and measured GFR were −0.30 (0.66) and −1.51 (1.33) mL/min/1.73 m^2, respectively<br>• Annualized rate of change in eGFR CKD-EPI from baseline to month 24, mean (SD), mL/min/1.73 m^2<br>○ Classic phenotype subgroup (*n* = 11): −0.3 (3.76) (95% CI: −2.80, 2.25)<br>○ Other patients (*n* =30): −0.3 (4.47) (95% CI: −2.0, 1.4)<br>• Annualized rate of change in mGFRiohexol,[a] mean (SD), mL/min/1.73 m^2<br>○ Classic phenotype subgroup (*n* = 9): −3.0 (6.04) (95% CI: −7.65, 1.64)<br>• Other patients (*n* = 28): −1.0 (8.66) (95% CI: −4.39, 2.33)
Giugliani 2013 [18]	9	48 weeks	• eGFR at baseline in females with FD, range, (mL/min/1.73 $m^{2)}$<br>○ Amendable *GLA* mutations: 76, 116<br>○ Non-amendable *GLA* mutations: 73, 108<br>• eGFR at week 48 in females with FD, range, (mL/min/1.73 m^2)<br>○ Amendable *GLA* mutations: 68, 120<br>○ Non-amendable *GLA* mutations: 73, 152
Hopkin 2022* [19]	78	Median [IQR]: 5.1 [2.3, 6.8] years	• Patients on migalastat for a median [IQR] of 5.9 [4.7, 7.0] years (*n* = 78) had stable mean eGFR (SD) of −1.57 (3.33) mL/min/1.73 m^2/year
Lenders 2020 [20], Lenders 2021 [21]	59	24 months	• Mean (SD) eGFR (mL/min/1.73 m^2)<br>○ Female: baseline, 98.6 (15.2); follow-up at 12 months, 92.9 (19.4); *p* = 0.0059<br>○ Male: baseline, 99.8 (22.6); follow-up at 12 months, 96.6 (23.0); *p* = 0.0011<br>○ Female: baseline, 98.6 (15.2); follow-up at 24 months, 90.4 (14.7); *p* = 0.0317<br>○ Male: baseline, 99.8 (22.6); follow-up at 24 months, 92.4 (24.0); *p* = 0.0028

Author, year	*N*	Treatment duration<br>*Treatment detail*	Key results
			• Mean (SD) eGFR slope at 12 months (mL/min/1.73 m^2/year)<br>○ Patients with unstable kidney function (≥ 3 mL/min/1.73 m^2/year, *n* = 28): −10.4 (7.3)<br>○ Patients with stable kidney function (< 3 mL/min/1.73 m^2/year, *n* = 20): 0.4 (2.4); *p* < 0.0001
Orsborne 2020 [22]	78	Median [range]: 594 days [235, 791]	• Mean eGFR declined from 81 to 79 mL/min/1.73 m^2
Ramaswami 2022* [23]	21	1 year	• Mean (SD) baseline eGFR in adolescent patients: 117.0 (19.93) mL/min/1.73 m^2<br>○ Mean (SD) annualized eGFR change (*n* = 19), −1.5 (15.11) mL/min/1.73 m^2
Skuban 2017* [24]	120[a]	FACETS: 6 months; ATTRACT: 18 months with 12-month OLE	• FACETS, the mean annualized rate of change over 24 months in eGFR CKD-EPI ± SEM with migalastat was −0.3 ± 0.7 mL/min/1.73 m^2<br>• ATTRACT, migalastat and ERT had comparable effects on renal function during the 18-month controlled period<br>• Compared with natural history, the rate of GFR decline in FACETS was slower than that of untreated patients when matched for gender and baseline proteinuria
Torra 2018* [25]	NR	FACETS: 24 months ATTRACT: 18 months	• eGFR stable in both renal subgroups<br>• Annualized rate of change:<br>○ FACETS: NR<br>○ ATTRACT:<br>○ Baseline eGFR 30–60 mL/min/1.73 m²: mean (SD): -3.3 (3.2)<br>○ Baseline eGFR 30 to ≥ 60 mL/min/1.73 m²: mean (SD): -0.4 (4.3)
West 2023* [26]	125	Mean: 3.9 years	• Mean (SD) annualized eGFR change (mL/min/1.73 m^2/year)<br>○ Overall cohort: −0.9 (4.9)<br>• Mean (SD) annualized eGFR change by eGFR category at enrolment (mL/min/1.73 m^2/year)<br>○ eGFR ≥ 90 (33.6% of patients): −1.0 (3.9)<br>○ eGFR 60–90 (43.2% of patients): −1.0 (5.9)<br>○ eGFR 30–60 (12.8% of patients): −0.4 (3.5)
**Pegunigalsidase alfa single-arm studies**			
Bernat 2022* [27]	30	1 year	• Patients were switched from agalsidase alfa or beta > 80% labelled dose/kg (7 patients [23.3%] received agalsidase alfa, 23 patients [76.6%] received agalsidase beta)<br>• eGFR values were stable with a mean (SE) change from baseline of −1.27 (1.39) mL/min/1.73 m^2 at 1 year (*n* = 29)
Bernat 2023* [28]	29	Mean [range]: 38.3 [25.3, 44.8] person-months	• eGFR change from baseline after 2 years of treatment (week 108)<br>○ Mean (SE; median): −5.10 (1.96; −4.23) mL/min/1.73 m^2<br>• Median [range] annualized eGFR slope (mL/min/1.73 m^2/year)<br>○ Overall: −2.47 [−8.7, 1.4]<br>○ Males: −2.82 [−8.7, 1.4]<br>○ Females: −1.45 [−6.5, 1.4]<br>○ ADA+: −3.49 [−8.7, 1)<br>○ ADA-: −1.45 (−8.5, 1.4]<br>○ eGFR > 120: −3.49 [−7.9, −1.2]<br>○ eGFR ≤ 120: −1.66 [−8.7, 1.4]<br>Statistical analyses not reported
Hughes 2023b [29]	16	12 months followed by 60 month open-label extension	• Baseline eGFR values, mL/min/1.73 m^2<br>○ All patients: mean, 111.7 (SE, 5.5; median, 114.29)<br>○ Males: mean, 118.1 (SE, 7.7; median, 116.76)<br>○ Females: mean, 104.4 (SE, 7.5; median, 105.70)<br>• eGFR values at 60 months, mL/min/1.73 m^2<br>○ All patients: mean, 97.0 (SE, 6.4; median, 101.98)

Author, year	*N*	Treatment duration<br>*Treatment detail*	Key results
			○ Males: mean, 100.0 (SE, 8.3; median, 103.89)<br>○ Females: mean, 92.4 (SE, 11.4; median, 93.84)<br>• Annualized eGFR slopes up to 72 months, mL/min/1.73 m^2/year<br>○ All patients: mean, −1.6 (SE, 0.8; median, −1.5)<br>○ Males: mean, −2.4 (SE, 0.9; median, −2.8)<br>○ Females: mean, −0.7 (SE, 1.3; median, −1.3)<br>○ Statistical analyses not reported
Schiffmann 2019 [30]	19	3 months with 9 months OLE	• Mean (SD) [range] eGFR mL/min/1.73 m^2<br>○ Baseline: 111.2 (20.9) [78, 156]<br>○ 12 months: 110.5 (23.4) [68, 152]<br>○ Change: −0.8 (7.7)<br>• In the 10 patients with classic phenotype FD: mean [range] annualized eGFR slope (mL/min/1.73 m^2/year; calculated from monthly measurement, total of 6–7 data points per patient), −1.8 [range −18.18, 6.35]<br>• Excluding one male patient who was treated with doxycycline, nine patients with classic FD: mean [range] eGFR slope, 0.01 [−6.35, 6.35], mL/min/1.73 m^2/year
**Lentivirus-mediated gene therapy single-arm studies**			
Khan 2021a [31]	5	Range: 12, 33 months	• eGFR increased during the treatment phase in all patients<br>• Following gene therapy, eGFR returned to baseline levels and was relatively stable in all patients, except for Patient 2. This patient displayed progressive CKD with significant proteinuria during screening<br>• eGFR slope/year was near zero for four of the five patients: range, −3.61, 3.01 mL/min/1.73 m^2/year

[a]mGFRiohexol, mGFR as assessed by plasma clearance of iohexol.

*Congress abstract.

CI, confidence interval. CKD, chronic kidney disease. CKD-EPI, Chronic Kidney Disease Epidemiology Collaboration. edf, empirical distribution function. eGFR, estimated glomerular filtration rate. EOW, every other week. ERT, enzyme replacement therapy. FD, Fabry disease. GFR, glomerular filtration rate. HR, hazard ratio. IQR, interquartile range. ITT, intention-to-treat. MDRD, Modification of Diet in Renal Disease. MRI, magnetic resonance imaging. NR, not reported. NS, not significant. Q1, first quartile. Q3, third quartile. SD, standard deviation. SE, standard error. SEM, standard error of the mean. UACR, urine albumin–creatinine ratio.

B

**Author, year**	**Treatment groups, *n***<br>***Treatment duration***	**Key results**
**Switch studies**		
Goker-Alpan 2015 [32]	Naïve (no history of prior ERT) agalsidase alfa, 29<br>Switch (switched from agalsidase beta) agalsidase alfa, 71<br><br>*≤ 24 months*	• CKD stage 1A, 1B, 2, 3, 4, *n*<br>○ Naïve: 3 (10.3%), 12 (41.4%), 7 (24.1%), 4 (13.8%), 3 (10.3%)<br>○ Switch: 5 (7.0%), 27 (38.0%), 21 (29.6%), 9 (12.7%), 9 (12.7%)<br>• In patients with no dialysis or kidney transplant experience, mean (SEM) baseline eGFR was 94.2 (7.65) mL/min/1.73 m^2 in naïve patients (*n* = 27) and 87.45 (4.40) mL/min/1.73 m^2 in switch patients (*n* = 59)<br>• eGFR remained clinically stable among naïve and switch patients (data shown in figure)<br>• In patients with no dialysis or kidney transplant experience, the mean (SEM) annualized rates of eGFR change were −1.68 (2.21) mL/min/1.73 m^2 in the naïve group (*n* = 27) and −2.40 (1.04) mL/min/1.73 m^2 in the switch group (*n* = 59)
Linhart 2023 [33]	Patients previously treated with agalsidase alfa for ≥ 2 years before starting pegunigalsidase alfa, 22<br><br>*12 months*	• All patients eGFR<br>○ Pre-switch: −5.9 (1.34) mL/min/1.73 m^2/year<br>○ Post-switch: −1.19 (1.77) mL/min/1.73 m^2/year<br>○ Mean (SD) change: + 4.70 (2.26) mL/min/1.73 m^2/year<br>• Men (*n* = 13)<br>○ Pre-switch: −6.36 (1.89) mL/min/1.73 m^2/year<br>○ Post-switch: −1.73 (2.64) mL/min/1.73 m^2/year<br>○ Mean (SD) change: + 4.63 (3.48) mL/min/1.73 m^2/year<br>• Women (*n* = 7)<br>○ Pre-switch: −5.03 (1.65) mL/min/1.73 m^2/year<br>○ Post-switch: −0.21 (1.47) mL/min/1.73 m^2/year<br>○ Mean (SD) change: + 4.83 (1.09) mL/min/1.73 m^2/year
Hughes 2017 [34], Narita 2020 [35]	Migalastat, 36<br>ERT, 24<br><br>*18 months*<br><br>Japanese population<br>• Migalastat, 5<br>• ERT, 1<br><br>*30 months*	• Migalastat: baseline GFR (mL/min/1.73 m^2), mean (SEM)<br>○ eGFR (CKD-EPI): 89.6 (3.7)<br>○ mGFR (iohexol): 82.4 (3.0)<br>○ eGFR (MDRD): 83.6 (3.7)<br>• ERT: baseline GFR (mL/min/1.73 m^2), mean (SEM)<br>○ eGFR (CKD-EPI): 95.8 (4.1)<br>○ mGFR (iohexol): 83.6 (5.2)<br>○ eGFR (MDRD): 87.8 (19.0)<br>• LS mean (SEM) annualized GFR from baseline to 18 months (mL/min/1.73 m^2/year)<br>○ Migalastat (*n* = 34)<br>– eGFR (CKD-EPI): −0.40 (0.93)<br>– mGFR (iohexol): −4.35 (1.64)<br>– eGFR (MDRD): −1.51 (0.95)<br>– In Japanese patients treated with migalastat (*n* = 5)<br>▪ eGFR (CKD-EPI): −1.8<br>▪ mGFR (iohexol): −7.6<br>○ ERT (*n* = 18)<br>– eGFR (CKD-EPI): −1.03 (1.29<br>– mGFR (iohexol): −3.24 (2.27)<br>– eGFR (MDRD): −1.53 (1.32)<br>– In Japanese patients receiving ERT (*n* = 1)<br>▪ eGFR (CKD-EPI): 1.6

Author, year	Treatment groups, *n*<br>*Treatment duration*	Key results
		▪ mGFR (iohexol): −6.4<br>• Mean (SD) GFR from baseline to 30 months and up to 48 months (mL/min/1.73 m^2)<br>○ In Japanese patients treated with migalastat (*n* = 5)<br>– eGFR (CKD-EPI): −2.94 (2.93)<br>– mGFR (iohexol): −3.39 (2.52)<br>▪ Stable up to month 48
Kramer 2018 [36]	Regular dose agalsidase beta, 37<br>Switch to agalsidase alfa group, 26<br>Re-switch group, 37 (patients re-switched to agalsidase alfa after agalsidase beta shortage)<br><br>*Long-term follow-up 1 (LTFU1); ≥ 12 months*<br>*Long-term follow-up 2 (LTFU2); ≥ 24 months*	• Mean (SD) baseline eGFR (creatinine-cystatin C) mL/min/1.73 m^2<br>○ Regular dose: 71 (40)<br>○ Switch: 89 (34)<br>○ Re-switch: 80 (34)<br>• Mean (SD) long-term follow-up 1 (LTFU1); change in eGFR from baseline to LTFU1; per cent change eGFR per year from baseline to LTFU1; LTFU2; change in eGFR from LTFU1 to 2; per cent change in LTFU1 to 2<br>○ Regular dose<br>– eGFR (creatinine-cystatin C): 93 (38); 4.2 (15.8), $p < 0.05$ vs switch and vs re-switch; 4.7 (19.1), $p < 0.05$ vs switch and re-switch; 90 (38); −2.4 (13.2), *p* < vs switch; −2.6 (14.9), *p* < vs switch<br>– eGFR (creatinine): 92 (32); 5.0 (14.2), $p < 0.05$ vs switch and vs re-switch; 5.6 (16.3), $p < 0.05$ vs switch and vs re-switch; 89 (37); −3.2 (11.2), $p < 0.05$ vs switch; −3.7 (13.1), $p < 0.05$ vs switch<br>– eGFR (cystatin C): 104 (34); 6.1 (19.2); 6.6 (19.2); 99 (45); −2.2 (9.8); −2.3 (10.1)<br>○ Switch<br>– eGFR (creatinine-cystatin C): 81 (28), $p < 0.05$ vs LTFU2; −6.0 (5.1); −5.5 (6.8); 75 (31); −4.6 (9.1), $p < 0.05$ vs re-switch; −5.7 (14.3), $p < 0.05$ vs re-switch<br>– eGFR (creatinine): 83 (31), $p < 0.05$ vs LTFU2; −7.2 (9.3); −6.2 (11.3); 72 (32); −8.1 (12.1), $p < 0.05$ vs re-switch; −10.6 (16.0), $p < 0.05$ vs re-switch<br>– eGFR (cystatin C): 3 (29); −8.9 (9.9); −8.8 (11.1); 89 (39); −5.4 (14.3); −6.0 (16.3)<br>○ Re-switch<br>– eGFR (creatinine-cystatin C): 76 (25), $p < 0.05$ vs LTFU2; −5.8 (8.9); −6.6 (12.5); 74 (31); −2.2 (4.4); −3.2 (5.9)<br>– eGFR (creatinine): 78 (23), $p < 0.05$ vs LTFU2; −5.6 (7.7); −7.5 (10.8); 77 (24); −1.8 (5.2); −5.3 (6.9)<br>eGFR (cystatin C): 87 (36); −6.1 (8.9); −5.8 (9.9); 86 (34); −1.3 (6.2); −1.4 (6.9)
Lenders 2016 [37]	Agalsidase beta regular dose, 24<br>Agalsidase beta dose reduction, 28<br>Switch (agalsidase alfa), 37<br><br>*2 years*	• Mean (SD) baseline eGFR (mL/min/1.73 m^2)<br>○ Regular dose<br>– eGFR (creatinine): 69.6 (36.6)<br>– eGFR (cystatin): 79.9 (42.4)<br>– eGFR (creatinine-cystatin): 73.7 (39.9)<br>○ Dose reduction<br>– eGFR (creatinine): 74.2 (34.5)<br>– eGFR (cystatin): 91.1 (46.7)<br>– eGFR (creatinine-cystatin): 83.3 (41.2)<br>○ Switch<br>– eGFR (creatinine): 72.8 (35.9)<br>– eGFR (cystatin): 89.4 (37.9)<br>– eGFR (creatinine-cystatin): 83.7 (34.1)<br>• Change in eGFR per year (95% CI), (mL/min/1.73 m^2/year)<br>○ Regular dose<br>– eGFR (creatinine): 0.13 (−3.75, 4.00)<br>– eGFR (cystatin): −1.77 (−7.51, 3.98)<br>– eGFR (creatinine-cystatin): −0.47 (−4.50, 3.55)

Author, year	Treatment groups, *n*<br>*Treatment duration*	Key results
		○ Dose reduction (*n* = 15)<br>– eGFR (creatinine): −3.74 (−6.63, −0.85)<br>– eGFR (cystatin): −14.02 (−19.43, −8.60)<br>– eGFR (creatinine-cystatin): −9.33 (−13.32, −5.34)<br>○ Switch (*n* = 22)<br>– eGFR (creatinine): −2.45 (−4.67, −0.24)<br>– eGFR (cystatin): −8.64 (−12.62, −4.65)<br>eGFR (creatinine-cystatin): −4.97 (−7.73, −2.20)
Lenders 2021 [38]	Agalsidase beta regular dose, 17<br>Switch, 22 (agalsidase beta for ≥ 12 months, then dose-reduced and subsequently switched to agalsidase alfa)<br>Re-switch, 39 (treated with agalsidase beta for ≥ 12 months, then dose-reduced or switched to agalsidase alfa for ≥ 24 months and then re-switched to agalsidase beta)	• Mean (SD) baseline eGFR (mL/min/1.73 m^2)<br>○ Agalsidase beta: 103 (24)<br>○ Switch: 102 (30)<br>○ Re-switch: 80 (29)<br>• Mean (SD) follow-up eGFR (mL/min/1.73 m^2)<br>○ Agalsidase beta: 87 (31)<br>○ Switch: 83 (34)<br>○ Re-switch: 66 (28)<br>• Mean (SD) eGFR change per year (mL/min/1.73 m^2/year)<br>○ Agalsidase beta: −1.7 (3.1)<br>○ Switch: −3.3 (4.2), $p = 0.01$<br>○ Re-switch: −2.7 (2.8), $p < 0.01$
Müntze 2019 [39], Muentze 2018* [40]	Migalastat, treatment naïve, 8<br>Migalastat, switch from ERT, 6	• Mean [IQR] GFR CKD-EPI (mL/min/1.73 m^2)<br>○ All patients (*n* = 13)<br>– Baseline: 87 [75.5, 102]<br>– Follow-up 1 (3–6 months): 84 [73.5, 95.5]<br>– Follow-up 2 (12 months): 78 [71.5, 99]; $p = 0.012$<br>○ Treatment-naïve (*n* = 7)<br>– Baseline: 76 [69, 95]<br>– Follow-up 1 (3–6 months): 75 [68, 84]<br>– Follow-up 2 (12 months): 72 [65, 78]; $p = 0.006$<br>○ Switch from ERT (*n* = 6)<br>– Baseline: 95.5 [84.3, 108]<br>– Follow-up 1 (3–6 months): 95 [87.8, 96.8]<br>– Follow-up 2 (12 months): 95 [88.8, 103.3]; $p = 0.607$
Riccio 2020 [41]	ERT switch to migalastat, 7<br><br>*12 months*	• Mean (SD) eGFR in male patients, mL/min/1.73 m^2<br>○ Baseline: 102.57 (40.45)<br>○ 12 months ERT: 99.85 (41.11)<br>○ 12 months migalastat: 98.28 (40.46)
Tsuboi 2012 [42], Tsuboi 2014 [43]	Agalsidase beta switch to agalsidase alfa, 11<br><br>*≤ 12 months*	• eGFR, mL/min/1.73 m^2, in group treated with agalsidase beta, before switch<br>○ Mean (SD): 89.97 (21.51)<br>○ Median [range]: 93.4 [54.7, 124.4]<br>• eGFR, mL/min/1.73 m^2<br>○ 6 months after switch<br>– Mean (SD): 89.99 (21.52)<br>– Median [range]: 86.8 [52.6, 122.2]

Author, year	Treatment groups, *n*<br>*Treatment duration*	Key results
		○ 12 months after switch<br>– Mean (SD): 90.60 (22.28)<br>– Median [range]: 89.9 [54.7,120.0]<br>– *p* = 0.9844<br>• eGFR, which had been stable during the 24 months prior to the switch, did not change significantly during the 12 months after switching
Weidemann 2014 [44]	Agalsidase beta regular dose, 38<br>Agalsidase beta dose reduction, 29<br>Switch (agalsidase alfa), 38<br><br>*≤ 12 months*	• eGFR, mL/min/1.73 m^2<br>○ Agalsidase beta regular dose group, median [range]<br>– GFR (cystatin-C) (*n* = 22): 1-year retrospective visit, 91 [21, 125], *p* = 0.44; baseline visit, 104 [18, 158], *p* = 0.98; 1-year follow-up visit, 101 [49, 151]<br>– eGFR (CKD-EPI) (*n* = 37): 1-year retrospective visit, 98 [64, 120], *p* = 0.25; baseline visit, 99 [63, 119], *p* = 0.49; 1-year follow-up visit, 101 [58, 120]<br>– eGFR (CKD-EPI) men only: 1-year retrospective visit, 99 [64, 120], *p* = 0.55; baseline visit, 101 [63, 119], *p* = 0.69; 1-year follow-up visit, 101 [58, 120]<br>– eGFR (CKD-EPI) women only: 1-year retrospective visit, 93 [72, 118], *p* = 0.61; baseline visit, 91 [71, 117], *p* = 0.13; 1-year follow-up visit, 99 [75, 119]<br>○ Agalsidase beta dose reduction group, median [range]<br>– GFR (cystatin-C) (*n* = 13): 1-year retrospective visit, 96 [35, 132], *p* = 0.33; baseline visit, 109 [65, 132], *p* = 0.02; 1-year follow-up visit, 92 [69, 135]<br>– eGFR (CKD-EPI) (*n* = 27): 1-year retrospective visit, 94 [61, 116], *p* = 0.34; baseline visit, 98 [86, 114], *p* = 0.01; 1-year follow-up visit, 95 [82, 114]<br>– eGFR (CKD-EPI) men only: 1-year retrospective visit, 94 [61, 116], *p* = 0.49; baseline visit, 102 [86, 112], *p* = 0.02; 1-year follow-up visit, 99 [82, 114]<br>– eGFR (CKD-EPI) women only: 1-year retrospective visit, 94 [84, 112], *p* = 0.16; baseline visit, 95 [86, 114], *p* = 0.05; 1-year follow-up visit, 92 [82, 110]<br>○ Agalsidase alfa switch group, median [range]<br>– GFR (cystatin-C) (*n* = 23): 1-year retrospective visit, 108 [53, 130], *p* = 0.47; baseline visit, 101 [52, 151], *p* = 0.09; 1-year follow-up visit, 96 [64, 141]<br>– eGFR (CKD-EPI) (*n* = 37): 1-year retrospective visit, 99 [68, 123], *p* = 0.53; baseline visit, 99 [66, 112], *p* = 0.05; 1-year follow-up visit, 95 [64, 116]<br>– eGFR (CKD-EPI) men only: 1-year retrospective visit, 103 [79, 123], *p* = 0.70; baseline visit, 99 [71, 112], *p* = 0.19; 1-year follow-up visit, 97 [64, 101]<br>– eGFR (CKD-EPI) women only: 1-year retrospective visit, 98 [68, 112], *p* = 0.25; baseline visit, 99 [66, 112], *p* = 0.31; 1-year follow-up visit, 93 [68, 116]

[a]Because of a shortage of agalsidase beta in 2010/2011, most patients subsequently received agalsidase alfa 0.2 mg/kg for the remaining follow-up period.

*Congress abstract.

CI, confidence interval. CKD, chronic kidney disease. CKD-EPI, Chronic Kidney Disease Epidemiology Collaboration. eGFR, estimated glomerular filtration rate. EOW, every other week. ERT, enzyme replacement therapy. GFR, glomerular filtration rate. IQR, interquartile range. ITT, intention-to-treat. LS, least-squares. MDRD, Modification of Diet in Renal Disease. SD, standard deviation. SEM, standard error of the mean.

Supplementary Table S5

Overview of proteinuria data from (A) single-arm studies and (B) comparator and switch studies.

A

Author, year *Study identifier*	*N*	Treatment duration	Results
**Agalsidase alfa single-arm studies**			
Cybulla 2022 [45] *FOS*	121	Mean / median (SD) [range]: 12.6 / 11.9 (5.0) [5.0, 21.6] years	• Annual rate of change in urinary protein in male patients (g/24 h), mean (SEM)<br>○ Low proteinuria (≤ 0.5 g/24 h; *n* = 82): 0.02 (0.01); *p* = 0.0028 vs baseline<br>○ High proteinuria (> 0.5 g/24 h; *n* = 39): 0.03 (0.01); *p* = 0.0222 vs baseline<br>○ *p* = 0.7531 low vs high proteinuria
Feriozzi 2012 [46] *FOS*	208	Mean [range]: 7.4 [5.0, 11.2] years	• Change in proteinuria to year end (mg/24 h), mean (SD)<br>○ Males year 5 (*n* = 61): 124.5 (944.5); *p* = 0.31<br>○ Males year 5+ (*n* = 84): 182.1 (71.6); *p* = 0.06<br>○ Females year 5 (*n* = 39): −18.9 (413.0); *p* = 0.78<br>○ Females year 5+ (*n* = 46): 88.9 (1024.5); *p* = 0.56
Goker-Alpan 2016 [47]	14	Median [range]: 54.5 [54.0, 59.0] weeks	• Changes from baseline at week 55 (mg/dL) in children, mean [95% CI] / median [range]<br>○ Urine protein: −1.79 [−5.95, 2.37] / 0.00 [−26.2, 3.5]<br>○ Urine microalbumin: 0.61 [−0.53, 1.75] / 0.00 [−0.7, 7.1]
Hughes 2011 [48] *FOS*	113	4 years	• Protein (mg/24 h) at baseline / 4 years, median [10th, 90th percentile]<br>○ Females (*n* = 36): 199.5 [50.0, 910.0] / 206.0 [80.0, 800.0]; *p* = 0.800 vs baseline<br>○ Males (*n* = 77): 333.0 [99.2, 2200.0] / 370.0 [100.0, 1700.0]; *p* = 0.465 vs baseline
Jardim 2006 [49]	6	2 years	• Proteinuria (mg/24 h), range<br>○ Baseline: 100, 13000<br>○ 24 months: 50, 1500
Kampmann 2015 [50]	45	Median [range]: 10.8 [9.6, 12.5] years	• Change in urinary protein levels over 10 years (mg/24 h), mean (SD)<br>○ Males (*n* = 8): 112.5 (35.8); NS<br>○ Females (*n* = 6): 115.3 (48.0); NS<br>○ Males with proteinuria at baseline (≥ 200 mg/24 h; *n* = 10): baseline 659.0 (889.0); 10-year change 297.0 (376.7); *p* = 0.0342<br>○ Females with proteinuria at baseline (*n* = 7): 507.6 (388.9); NS
Ries 2006 [51]	19	6 months	• Proteinuria in children without proteinuria or microalbuminuria at baseline (*n* = 15; mg/24 h), median [range]<br>○ Baseline: 8.6 [1.7, 19.2]<br>○ 6 months: 8.9 [2.4, 31.2]<br>• Albumin excretion in children with microalbuminuria at baseline (*n* = 4; mg/24 h), median [range]<br>○ Baseline: 50 [39.7, 108]<br>○ 6 months: 27.6 [15.9, 74.9]
Sasa 2019 [52]	493	Years: *n*<br>• < 0.5: 35 (7.1%)<br>• > 0.5–1: 31 (6.3%)<br>• > 1–2: 48 (9.7%)<br>• > 2–3: 69 (14.0%)<br>• > 3–4: 86 (17.4%)<br>• > 4–5: 132 (26.8%)<br>• > 5–6: 55 (11.2%)	• Change in proteinuria from baseline (*p* value vs baseline) in males / females (mg/day), mean (SD)<br>○ Year 0.5: 28 (592); *p* = 0.625 / 33 (403); *p* = 0.445<br>○ Year 1: −44 (483); *p* = 0.367 / 58 (387); *p* = 0.162<br>○ Year 2: 62 (632); *p* = 0.354 / 25 (337); *p* = 0.511<br>○ Year 3: 60 (551); *p* = 0.348 / 12 (348); *p* = 0.795<br>○ Year 4: 244 (567); *p* = 0.003 / 98 (389); *p* = 0.153<br>○ Year 5: 473 (787); *p* = 0.007 / 181 (418); *p* = 0.129<br>○ Year 6: 737 (1042); *p* = 0.144 / 121 (222); *p* = 0.141

Author, year *Study identifier*	*N*	Treatment duration	Results
		• > 6–7: 20 (4.1%)<br>• > 7–8: 17 (3.4%)	○ Year 7: 337 (477); *p* = 0.189 / −39 (56); *p* = 0.504
Schiffmann 2006 [53]	20	≥ 36 months	• Change in protein level (mg/24 h) in males, median [range]: 7 [−3.00, 2.72]<br>• Post-treatment protein level (mg/24 h), median: 543
Schiffmann 2007 [54]	12	2 years	• Urinary protein excretion (mg/24 h) in males, mean (SD)<br>○ Baseline: 1217 (1246)<br>○ After long-term EOW dosing: 1485 (1295)<br>○ After 2 years of weekly dosing: 1271 (1131); *p* = 0.41 vs EOW dosing
Schiffmann 2013 [55] *3 RCTs and extensions pooled*	73	1 year	• Change from baseline to 12 months in urinary protein excretion (mg/24 h), mean (SD): −0.1 (0.7)
Schiffmann 2014 [56]	17	Mean (SD): 6.5 (0.6) years	• None of the 8 children with proteinuria during treatment had proteinuria for more than three assessments, nor during their final visit
Schwarting 2006 [57] *FOS*	20	≥ 3 years	• Analysis of proteinuria before and during ERT showed inconsistent findings with some patients showing an increase and some others showing a decrease of urinary protein excretion
Thofehrn 2009 [58]	9	12–36 months	• 24 h proteinuria (mg/mL), mean (SD)<br>○ 3 months (*n* = 8): 400.75 (639.67)<br>○ 6 months (*n* = 7): 396.28 (656.90)<br>○ 12 months (*n* = 8): 317.87 (493.10)<br>○ 18 months (*n* = 7): 366.14 (564.49)<br>○ 24 months (*n* = 5): 203.40 (123.60)<br>○ 36 months (*n* = 5): 555.60 (572.57)<br>○ No significant differences were found
West 2009 [59]	108	Mean (SD): 2.0 (1.0) years	• Proteinuria (g/day) in males, mean (SD) / median [range]<br>○ Baseline (*n* = 80): 1.030 (1.680) / 0.40 [0.006, 10.550]<br>○ After 1 year (*n* = 76): 0.970 (1.520) / 0.420 [0.040, 8.660]<br>○ After 2 years (*n* = 34): 0.970 (1.140) / 0.610 [0.036, 4.680]
**Agalsidase beta single-arm studies**			
Breunig 2006 [60]	10	Mean [range]: 22.7 [12, 37] months	• Proteinuria (mg/day) in patients with eGFR > 90 mL/min/1.73 m^2 (*n* = 6) / < 90 mL/min/1.73 m^2 (*n* = 4), mean (SEM)<br>○ Baseline: 845 (714) / 2091 (743)<br>○ Follow-up: 834 (816) / 102 (1216)<br>○ *p* value baseline vs follow-up = 0.96 / 0.3
Goicoechea 2020* [61]	69	Median [range]: 60 [24, 120] months	• UACR (mg/g)<br>○ Baseline: 242<br>○ Follow-up: 128<br>○ *p* = 0.05 baseline vs follow-up
Hwang 2022 [62]	10	22 weeks	• Change from baseline to week 22 proteinuria (mg/d), mean (SD)<br>○ 157.14 (379.45); *p* = 0.492 vs baseline
Lubanda 2009 [63]	21	96 weeks	• UPCR (g/g), median [range]<br>○ Baseline: 0.3 [0.1, 5.0]<br>○ 24 weeks: 0.4<br>○ 96 weeks: 0.3

Author, year<br>*Study identifier*	*N*	Treatment duration	Results
Ramaswami 2019 [64]<br>*NCT00701415*	31	5 years	• Change from baseline to year 5 UACR (mg/g) in male paediatric patients, median [range]<br>○ −2.0 [−23.0, 51.0]; *p* = 0.0761<br>• Change from baseline to year 5 UPCR (mg/g) in male paediatric patients, median [range]<br>○ −27.3 [−125.1, 164.9]; *p* = 0.0006
Warnock 2012 [65]<br>*Fabry Registry*	213	Mean (SD)<br>• Males: 5 (1.9) years<br>• Females: 4.0 (1.6) years	• Logistic regression modelling of renal disease progression in patients split by quartiles based on eGFR slopes during treatment, OR [95% CI]<br>• UPCR was strongly associated with renal disease progression when men in Q1 and Q4 were compared<br>○ , OR [95% CI] Males (*n* = 75): 112 [4.0, 3108.9]; *p* = 0.0054<br>○ Females (*n* = 62): 11.7 [1.1, 119.7]; *p* = 0.0388
Warnock 2015 [66]<br>*FAACET; NCT00446862*	24	Median [IQR]:<br>3.1 [0.3, 4.4] years	• Safety and efficacy of antiproteinuric therapy with angiotensin-converting enzyme inhibitors and angiotensin II receptor blockers in Fabry patients with nephropathy receiving ERT<br>• UPCR (g/g), median [IQR]<br>○ Overall: 0.7 [0.4, 1.3]<br>○ Patients who met UPCR goal (<0.5 g/g): 0.6 [0.3, 1.1]<br>Patients above UPCR goal: 1.2 [0.4, 2.3]
Weidemann 2013 [67]<br>*Fabry Registry*	40	Median [IQR]: 6.0 [5.1, 7.2] years	• Proteinuria (mg/day), mean (SD)<br>○ Baseline: 633 (961)<br>○ Follow-up: 209 (215)<br>○ *p* = 0.018 baseline vs follow-up
Wraith 2008 [68]	16	48 weeks	• Rate of urinary protein excretion (mg/m^2/24 h) in children, mean (SD)<br>○ Baseline: 118 (55)<br>○ Week 24 (*n* = 15): 77 (29)<br>○ Week 48 (*n* = 13): 95 (35)
**Mixed or non-specified ERT single-arm studies**			
Goicoechea 2021 [2]	69	Median: 5 years	• Albuminuria (mg/g) in males / females, median [range]<br>○ Baseline: 242 [65, 1200] / 123 [10, 786]<br>○ After 5 years on ERT: 540 [40, 958] / 86 [21, 176]
Madsen 2019 [5]<br>*NCT02969200*	52	Median [range]:<br>335 [23, 636] weeks	• Urine albumin (mg/day) / urine protein (g/day) / UACR (mg/g), median [range]<br>○ Baseline: 91 [0, 3284] / 0.2 [0.0, 4.0] / 33 [0, 2898]<br>○ Follow-up: 29 [0, 4172] / 0.1 [0.0, 5.6] / 19 [0, 4250]<br>○ *p* value baseline vs follow-up = 0.6 / 0.5 / 0.2
Skrunes 2017 [10]	19	Median [range]<br>• Total cohort: 9.4 [4.8, 13.8] years<br>• Lower dose: 7.8 [4.9, 9.9] years<br>• Higher dose: 10.5 [4.8, 13.8] years	• Change in UACR (mg/mmol), mean [95% CI]<br>○ Lower dose: 4.34 [−5.26, 13.94]<br>○ Higher dose: −8.64 [−24.79, 7.51]
Wyatt 2012 [12], Anderson 2014 [13] *NCS-LSD*	311	Mean, median (SD) [range]<br>• Adult males: 3.74, 3.51 (2.66) [0, 9.72]<br>• Adult females: 3.34, 3.55 (2.25) [0, 8.77]	• OR [95% CI] of having high proteinuria with time on ERT of < 12 months / 12–36 months / > 36 months<br>○ Males (*n* = 106): 0.78 [0.18, 3.38]; *p* = 0.74 / 0.68 [0.18, 2.51]; *p* = 0.57 / 0.98 [0.25, 3.89]; *p* = 0.98<br>○ Females (*n* = 158): 0.49 [0.17, 1.42]; *p* = 0.27 / 0.44 [0.18, 1.10]; *p* = 0.12 / 0.44 [0.16, 1.17]; *p* = 0.14

Author, year *Study identifier*	*N*	Treatment duration	Results
**Migalastat single-arm studies**			
Ramaswami 2022 [23] *ASPIRE**	21	Follow-up, 1 year	• Urine protein remained stable in adolescent patients
**Pegunigalsidase alfa single-arm studies**			
Schiffmann 2019 [30] *NCT01678898 and NCT01769001*	18	1 year	• Number of patients with UPCR at baseline / 12 months<br>○ Normal (< 200 mg/g): 12 / 6<br>○ Above normal (≥ 200 mg/g): 4 / 2<br>• Two patients with the highest baseline UPCR demonstrated a decrease during treatment

*Congress abstract.

CI, confidence interval. eGFR, estimated glomerular filtration rate. EOW, every other week. ERT, enzyme replacement therapy. IQR, interquartile range. NR, not reported. NS, not significant. OR, odds ratio. SD, standard deviation. SEM, standard error of the mean. UACR, urine albumin–creatinine ratio. UPCR, urine protein–creatinine ratio.

B

Author, year *Study identifier*	Treatment groups, *n*	Key results
**Comparator studies**		
Cybulla 2009 [69] *FOS*	Kidney transplant with ERT, 20<br>Kidney transplant no ERT, 16	• Proteinuria (mg/24 h), median [IQR]<br>○ Baseline<br>– ERT (*n* = 20): 240 [200, 885]<br>– No ERT (*n* = 7): 420 [100, 1300]<br>○ After 2 years<br>– ERT (*n* = 4): 219
Germain 2016 [16], Germain 2019 [17] *NCT00925301 [FACETS] and NCT01458119*	Migalastat, 28<br>Placebo then migalastat, 22	• Change from baseline urinary protein (mg/24 h), mean (SD)<br>○ Baseline to 6 months<br>– Migalastat to migalastat: 2.2 (252)<br>– Placebo to migalastat: −12.9 (224)<br>○ Baseline to 12 months<br>– Migalastat to migalastat: 77.1 (153)<br>– Placebo to migalastat: −17 (271)<br>○ Baseline to 24 months<br>– Migalastat to migalastat: 139.2 (306)<br>– Placebo to migalastat: 251.1 (632)
Golan 2015 [70] *NCT01124643*	Agalsidase alfa 0.2 mg/kg EOW, 20<br>Agalsidase alfa 0.2 mg/kg weekly, 19<br>Agalsidase alfa 0.4 mg/kg weekly, 5	• Change from baseline to week 53 in UACR, median [95% CI]<br>○ 0.2 mg/kg EOW: −7.0 [−237.3, 405.2]<br>○ 0.2 mg/kg weekly: −6.0 [−219.9, 111.8]<br>○ 0.4 mg/kg weekly: −64.0 [−420.3, 311.9]<br>• The median UACR in each 0.2 mg/kg group remained stable over 53 weeks, and no statistically significant difference was found in the change from baseline to week 53 between the two 0.2 mg/kg groups
Guerard 2018 [71]	Lucerastat + ERT, 10<br>ERT, 4	• UACR (mg/g) at day 1 / week 4 / week 8 / week 12, mean (SD)<br>○ Lucerastat + ERT: 56.9 (55.1) / 51.8 (22.3) / 41.2 (25.2) / 47.1 (44.2)<br>○ ERT: 917.7 (809.8) / 531.3 (358.1) / 801.5 (283.1) / 1334.8 (1430.1)

Author, year<br>*Study identifier*	Treatment groups, *n*	Key results
Hughes 2019*<br>[72] *FOS*	Agalsidase alfa, 74<br>Untreated, 57	• Changes in urinary protein from before to after pregnancy (mg/24 h), mean (SD)<br>○ ERT: −123.7 (355.6)<br>○ Untreated: 16.7 (51.4)
Prabakaran<br>2014 [73]	ERT, 13<br>Untreated, 4	• Change in UACR per year, mean (95% CI)<br>○ ERT: −22% (11%, 34%); $p < 0.0001$ vs baseline<br>○ Untreated: 39% (15%, 67%); $p < 0.0001$ vs baseline<br>○ $p < 0.0001$ ERT vs untreated
van der Veen<br>2022 [74]<br>*NCT00701415*	Agalsidase beta, 7<br>Untreated, 23	• UACR (mg/mmol) at 10-year follow-up in male patients, median [range]<br>○ Agalsidase beta: 0.4 [0, 8.8]<br>○ Untreated: 3.7 [0, 248]<br>○ $p = 0.02$ agalsidase beta vs untreated
Vedder 2007<br>[75]	Agalsidase alfa, 18<br>Agalsidase beta, 16	• Proteinuria (g/24 h) agalsidase alfa/agalsidase beta, median [range]<br>○ Baseline: 0.25 [0.06, 2.65] / 0.24 [0.10, 0.68]; $p = 0.59$ between groups<br>○ 12 months: 0.30 [0.08, 2.83] / 0.20 [0.08, 0.50]; $p = 0.16$ between groups<br>○ 24 months: 0.27 [0.10, 1.65] / 0.15 [0.06, 0.57]; $p = 0.33$ between groups<br>○ Change over time from baseline: p = NS at all time points
**Switch studies**		
Riccio 2020 [41]	ERT switch to migalastat, 7	• Proteinuria (mg/24 h) in male patients, mean (SD)<br>○ Baseline: 145.00 (237.19)<br>○ 1-year ERT: 135.00 (177.69)<br>○ 1-year migalastat: 78.57 (128.63)<br>○ $p = 0.048$ migalastat vs ERT
Ripeau 2017<br>[76]	Agalsidase beta switch to agalsidase alfa, 33	• UPCR (mg/g), mean SD<br>○ Baseline: 0.66 (0.27)<br>○ 1 year: 0.81 (0.29)<br>○ 2 years: 0.73 (0.33)<br>○ p = NS compared with baseline<br>• Urine protein excretion (mg/24 h), mean (SD)<br>○ Baseline: 708.2 (259.5)<br>○ 1 year: 855.2 (291.9)<br>○ 2 years: 904.0 (362.4)

CI, confidence interval. EOW, every other week. ERT, enzyme replacement therapy. LS, least-squares. NS, not significant. Q1, first quartile. Q3, third quartile. SD, standard deviation. UACR, urine albumin–creatinine ratio. UPCR, urine protein–creatinine ratio.

Supplementary Table S6

Overview of LVMI data from (A) mixed or non-specified ERT single-arm studies and single-arm studies for other treatments and (B) switch studies.

A

Author, year	*N*	Treatment duration<br>***Assessment method***	Key results
**Mixed or non-specified ERT single-arm studies**			
Arends 2017a [1]	293	Median [range] follow-up time: 6.8 [0.8, 15.4] years<br><br>*Echocardiography*	• 100 patients changed in dose or preparation during follow-up. In 78 patients the dose of agalsidase beta was temporarily lowered or switched to agalsidase alfa, mainly as a result of shortage of agalsidase beta<br>• Effect of age, sex and phenotype on LVMI reduction during first year, HR (95% CI);<br>○ Age (per 10 years): −1.6 (−4.1, 1.0)<br>○ Men with classic FD (*n* = 121): −5.9 (−9.6, −2.2); $p < 0.01$<br>○ Men with non-classic FD (*n* = 42): −0.8 (−8.5, 6.9)<br>○ Women with classic FD (*n* = 82): −6.3 (−11.3, −1.3); $p < 0.05$<br>○ Women with non-classic FD (*n* = 48), −0.4 (−7.0, 6.1)<br>• In patients without LVH there was no change in LVMI during the first year (beta: 1.5 g/m^2; $p = 0.19$), while in patients with LVH at baseline a moderate decrease in LVMI was observed (beta: −5.4 g/m^2; $p < 0.001$)<br>• Comparison of LVMI in 64 adult and 21 paediatric male patients did not yield meaningful results
Effati 2023* [77]	12	5 years<br>*MRI*	• During treatment, 2/12 (16.7%) patients experienced increased LVMI<br>• No statistical analyses reported
Huang 2024 [78]	13	12 months<br>*Echocardiography*	• Reduction in LVMI following treatment with ERT from 88 (39) g/m^2 to 70 (30) g/m^2 ($p = 0.10$)
Hongo 2018 [79]	42	ERT duration, median [IQR]<br>• All patients: 10 [6.75, 11] years<br>• Male: 11 [10.5, 13]<br>• Female: 8 [4, 10] years; $p < 0.01$ vs male<br><br>*Echocardiography*	• At baseline: mean (SD) LVMI, males vs females: 107.5 (227.4) vs 86.8 (31.1) g/m^2, $p < 0.05$<br>• The mean (SD) rate of LVMI increase was 3.02 (3.41) g/m^2/year in male patients<br>• Male patients<br>○ Year: 0, 1, 2, 3, 4, 5, 6, 7, 8, 9, 10, 11<br>– LVMI (g/m^2), mean (SD): 107.5 (27.4), 108.8 (31.6), 111.5 (39.3), 109.9 (31.8), 108.0 (24.5), 113.7 (50.5), 112.2 (26.2), 113.0 (32.0), 115.8 (41.2), 131.5 (41.9), 128.7 (43.9), 132.7 (43.9)<br>– *N*: 17, 14, 13, 12, 12, 13, 15, 14, 12, 11, 14, 12<br>○ Male patients without LVH (LVMI > 115 g/m^2), slope mean (SD): 2.81 (3.69) g/m^2/year<br>○ Male patients with LVH, slope mean (SD): 3.40 (3.10) g/m^2/year<br>• Male patients with FD without excessive LVH<br>○ Year: 0, 1, 2, 3, 4, 5, 6, 7, 8, 9, 10, 11<br>– LVMI (g/m^2), mean (SD): 98.2 (18.1), 99.7 (24.9), 96.5 (21.8), 98.1 (22.0), 101.4 (20.7), 94.5 (24.0), 107.3 (23.9), 103.7 (22.7), 103.4 (29.7), 118.2 (33.3), 117.8 (37.1), 118.9 (32.3)<br>○ *N*: 14, 11, 10, 9, 10, 10, 13, 12, 10, 9, 12, 10<br>• The mean (SD) rate of LVMI increase was 1.69 (2.73) g/m^2/year in female patients<br>• Female patients<br>○ Year: 0, 1, 2, 3, 4, 5, 6, 7, 8<br>– LVMI (g/m^2), mean (SD): 86.8 (31.1), 92.2 (36.8), 98.2 (37.7), 94.2 (41.4), 96.2 (33.3), 93.2 (42.0), 100.8 (40.1), 101.0 (41.9), 102.6 (50.1)<br>– *N*: 25, 22, 22, 21, 19, 17, 16, 14, 12<br>○ Female patients without LVH (LVMI > 95 g/m^2), slope mean (SD): 1.00 (2.34) g/m^2/year

Author, year	*N*	Treatment duration<br>***Assessment method***	Key results
			○ Female patients with LVH, slope mean (SD): 3.47 (3.04) g/m^2/year
Kovacevic-Preradovic 2008 [80]	29	Mean (SD)<br>• Males: 37 (17) months<br>• Females: 39 (16) months<br><br>*Echocardiography*	• Agalsidase alfa (*n* = 22) or agalsidase beta (*n* = 5) EOW<br>• Two additional patients treated with agalsidase alfa at the beginning of the ERT switched to agalsidase beta<br>• LVMI (g/m^2), mean (SD)<br>○ Baseline: 111 (40)<br>○ Follow-up: 118 (54); $p = 0.21$ vs baseline
Lenders 2016 [81]	76	NR/NA<br><br>*Echocardiography*	• Patients treated with agalsidase alfa or agalsidase beta and were analysed by serum-mediated agalsidase inhibition status<br>• LVM did not significantly differ between both groups ($p = 0.22$)
Lin 2013 [3]	36	6–39 months<br><br>*Echocardiography*	• Taiwanese population, including 15 patients with IVS4 + 919G > A mutation<br>• Mean (SD) LVMI, g/m^2<br>○ At baseline: 68.2 (26.9)<br>○ At follow-up: 60 (26.1); $p = 0.008$
Liu 2014 [82]	36	Median [range]: 20 [13, 46] months<br><br>*Echocardiography*	• Taiwanese population with classic FD (*n* =13) or IVS4 + 919G > A (*n* =23 [16 males, 7 females]) variants<br>• Overall, mean (SD) LVMI was reduced significantly, from 70.9 (28.7) g/m$^{2.7}$ at baseline to 61.5 (28.6) g/m$^{2.7}$ ($p < 0.001$) after ERT while on follow-up<br>○ Males: 78.5 (31.2) g/m$^{2.7}$ vs 68. 3 (25.3) g/m$^{2.7}$; $p = 0.007$<br>○ Females: 63.3 (24.5) g/m$^{2.7}$ vs 54.6 (30.7) g/m$^{2.7}$; $p = 0.023$<br>○ Classic males (*n* = 2): a significantly reduced LVMI: 57.2 (0.1) g/m$^{2.7}$ vs 46.8 (1.0) g/m$^{2.7}$; $p = 0.037$<br>○ Classic females (*n* = 11): LVMI did not improve significantly: 65.8 (30.1) g/m$^{2.7}$ vs 62.6 (36.9) g/m$^{2.7}$; $p = 0.533$<br>○ Males with IVS4+ variant (*n* = 16): 81.1 (32.2) vs 71.0 (25.6) g/m$^{2.7}$, $p = 0.016$<br>○ Females with IVS4+ variant (*n* = 7): 59.4 (12.8) vs 42.0 (10.2) g/m$^{2.7}$, $p < 0.001$
Lin 2014 [4]	9	Agalsidase beta: range: 0.7, 88.6 months<br>Agalsidase alfa: > 12 months<br><br>*Echocardiography*	• Population from Taiwan<br>• Agalsidase beta treatment then switched to agalsidase alfa; no evaluation of switch effects<br>• LVMI after ERT, range: 44.1, 77.7 g/m$^{2.7}$<br>• LVMI change (%), range: −36, −2; $p = 0.073$
Miwa 2019 [6], Miwa 2018* [7]	30	Mean (SD): 7.2 (4.6) years<br><br>*MRI and echocardiography*	• Cohort of patients with FD in Japan<br>• LVMI, mean (SD) annual change from baseline<br>○ Follow-up cohort: 0.18 (1.03)<br>○ Males: 0.53 (1.35) g/m$^{2.7}$<br>○ Females: −0.16 (0.44) g/m$^{2.7}$
Nordin 2019 [83]	Pre-ERT group, 20<br><br>Established ERT, 18<br><br>No ERT, 18	Mean (SD): 1.1 (0.2) years<br><br>Median [range]<br>• Pre-ERT group: 1 year	• Pre-ERT (initiated ERT, *n* = 20)<br>○ Overall, there was no change in mean (SD) LVMI: 93 (42) g/m^2 at baseline to 92 (40) g/m^2 ($p = 0.186$) over 1 year after ERT initiation<br>○ Among LVH-positive patients, change in mean (SD) LVMI: −2 (3) g/m^2; $p = 0.048$<br>○ Among LVH-negative patients, no change in mean (SD) LVMI: 0 (4) g/m^2; $p = 0.766$<br>• Established ERT (advanced stable disease, *n* = 18)<br>○ Over 1 year, there was no significant difference in LVMI: 124 (45) g/m^2 at baseline to 125 (45) g/m^2 ($p = 0.070$)<br>○ LVH-positive vs LVH-negative: NA (sample size too small)

Author, year	*N*	Treatment duration<br>*Assessment method*	Key results
		• Established ERT: 4.2 [1.4, 12.2] years<br>• No ERT: 1 year<br><br>*MRI*	• No ERT (early disease, mostly females, *n* = 18)<br>○ Over 1 year, there was a small increase in LVMI: 65 (15) g/m^2 at baseline to 67 (16) g/m^2 (*p* = 0.005)<br>○ LVH-positive vs LVH-negative: NA (sample size too small)<br>• Interactions between all three groups revealed statistically significant difference in LVMI (*p* = 0.009; difference for pre-ERT vs both established ERT and no ERT)
Rombach 2013 [8]	57	5 years<br><br>*Echocardiography*	• Among males (*n* = 30), mean (SE) LVM increased significantly: 1.2 (0.3) g/m$^{2.7}$ per year (*p* < 0.001) during a median follow-up of 5.0 (range: 0.5, 10.1) years<br>• Among females (*n* = 27), mean (SE)_LVM changed by −0.3 (0.4) g/m$^{2.7}$ per year (*p* = 0.52) during a median follow-up of 5.0 (range: 1.1, 8.0) years<br>• LVM changes by baseline LVH status<br>○ LVH was present in 40.7% (11/27) of males at baseline<br>○ Mean (SE) LVM increased 1.0 (0.3) g/m$^{2.7}$ per year in males without LVH at baseline (*p* = 0.004) and 1.5 (0.5) g/m$^{2.7}$ in males with LVH at baseline (*p* = 0.008)<br>○ Differences in LVM were predicted by CKD stage only, not by type of ERT, dosage, antibodies, hypertension or age at start of ERT<br>○ LVH was present in 50% of females at baseline<br>○ In females, mean change (SE) in LVM was −0.3 (0.5) g/m$^{2.7}$ per year (*p* = 0.61) in those without LVH at baseline and −0.4 (0.6) g/m$^{2.7}$ (*p* = 0.48) in those with LVH at baseline<br>• Differences in response could not be explained by CKD stage, presence of hypertension, age at start of ERT, type of ERT or dosage
Schmied 2016 [84]	38	Median (SD): 6.4 (1.2) years<br><br>*ECG and echocardiography*	• Mean (SD) LVMI (g/m^2)<br>○ Males (*n* = 26): baseline: 139 (59), follow-up: 168 (53); *p* < 0.005 vs baseline<br>○ Females (*n* = 12): baseline: 95 (46), follow-up: 103 (64)<br>• Binary logistic regression analysis revealed an abnormal baseline ECG to be the only factor independently associated with disease progression (*p* < 0.005)<br>• While patients without any ECG abnormalities at the time of treatment initiation showed a stable LVMI during the 5-year follow-up, a significant disease progression was observed in the group with abnormal ECGs, mean (SD) LVMI<br>○ Males: 168 (34) g/m^2 vs 212 (42) g/m^2, *p* < 0.05<br>○ Females: 151 (30) g/m^2 vs 205 (41) g/m^2, *p* < 0.05
Sirrs 2014 [9]	362	• Median Cohort 1a (previously received ERT): 64 months<br>• Cohort 1b (started ERT): 59 months<br>• Cohort 1c (no ERT): 52 months<br><br>*Echocardiography*	• LVMI at follow-up, mean (SD) (g/m^2)<br>• Cohort 1a proteinuria at entry ≤ 0.5 g/day subgroup, annual change from baseline in LVMI (g/m^2/year), mean (SE)<br>○ Combined: 1.81 (1.4)<br>○ Female: 2.05 (2.09)<br>○ Male: 1.72 (1.82)<br>• Cohort 1a proteinuria at entry > 0.5 g/day subgroup, annual change from baseline in LVMI (g/m^2/year), mean (SE)<br>○ Combined: 0.44 (3.03)<br>○ Female: 2.06 (8.99)<br>○ Male: 0.22 (3.12)<br>• Cohort 1b proteinuria at entry ≤ 0.5 g/day subgroup, annual change from baseline in LVMI (g/m^2/year), mean (SE)<br>○ Combined: 0.77 (1.58)<br>○ Female: 1.43 (2.05)<br>○ Male: −1 (2.03)<br>• Cohort 1b proteinuria at entry > 0.5 g/day subgroup, annual change from baseline in LVMI (g/m^2/year), mean (SE)<br>○ Combined: 1.12 (2.05)

Author, year	N	Treatment duration<br>*Assessment method*	Key results
			○ Female: −0.49 (2.91)<br>○ Male: 3.02 (3.16)
Talbot 2015 [11]	25	≤ 10 years<br><br>*ECG and echocardiography*	• Assessed the impact of ESRD (CKD5) on CV outcomes in male patients in Australia with FD on ERT<br>• LVMI (g/m^2), mean (SD)<br>○ Baseline<br>– Non-CKD5 ($n = 15$): 48.5 (12.7)<br>– CKD5 ($n = 10$): 73.3 (23.1)<br>○ Per year of treatment<br>– Non-CKD5<br>▪ Year 1: 46.1 (12.7)<br>▪ Year 2: 50.2 (21.0)<br>▪ Year 5: 48.8 (13.0)<br>▪ Year 7: 48.6 (13.5)<br>▪ Year 10: 48.3 (13.5)<br>▪ $p = 0.96$ over time<br>– CKD5<br>▪ Year 1: 71.3 (19.3)<br>▪ Year 2: 78.1 (24.6)<br>▪ Year 5: 90. (30.1)<br>▪ Year 7: 84.1 (30.3)<br>▪ Year 10: 100.2 (39.7)<br>▪ $p < 0.0001$ vs non-CKD5
Weidemann 2009 [85]	32	3 years<br><br>*Echocardiography*	• The benefit of ERT was assessed in patients at an early stage of the disease showing no or little myocardial fibrosis (patients with no fibrosis, mild fibrosis or severe fibrosis)<br>• LVM (g) during ERT by fibrosis status, mean (SD)<br>○ No fibrosis ($n = 12$): baseline, 238 (42); 1 year, 213 (46); 2 years, 201 (65); 3 years, 202 (46); $p = 0.01$<br>○ Mild fibrosis ($n = 11$): baseline, 275 (62); 1 year, 244 (60); 2 years, 234 (47); 3 years, 244 (65); $p = 0.31$<br>○ Severe fibrosis ($n = 9$): baseline, 303 (84); 1 year, 255 (58); 2 years, 246 (70); 3 years, 247 (45); $p = 0.24$
Wyatt 2012 [12], Anderson 2014 [13]	311	36 months<br><br>*Echocardiography*	• Patient data drawn from the UK National Collaborative Study of Lysosomal Storage Disorders<br>• LVMI (g/m^2), mean at start of ERT; change from baseline (95% CI) < 12 months; 12–36 months; > 36 months<br>○ All adults ($n = 277$): 107.4; 2.59 (−6.95, 12.2); −2.32 (−10.2, 5.56); −8.17 (−16.4, 0.006); $p = 0.11$<br>○ Children ($n = 20$): 68.3; −2.12 (−19.2, 15.0); −1.87 (−13.9, 10.2); −4.64 (−24.2, 14.9); $p = 0.92$<br>• The range of LVMI for adults ($n = 277$) was 37–371 g/m^2<br>○ A statistically significant increase in LVMI with age was seen in adults ($p < 0.001$)<br>○ After adjusting for age, there was no statistically significant association between LVMI and time on ERT ($p = 0.11$) when time on ERT was categorized as ‘not treated’, < 12 months, 12–36 months and > 36 months<br>○ When time was treated as a continuous variable, ERT had a significant linear effect of reducing LVMI (edf = 1; $p = 0.01$)<br>• The range of LVMI for children ($n = 9$) was 41–101 g/m^2<br>○ LVMI was not significantly associated with age in children ($p = 0.63$)<br>○ There was no significant association between LVMI in children and time on ERT (edf = 1.0; $p = 0.78$)
**Migalastat single-arm studies**			
Camporeale 2023 [86]	16	18 months<br><br>*MRI*	• MAIORA study<br>• Baseline ($n = 15$): median [range] LVMI: 99.0 [69.0–121.0] g/m^2,<br>• Follow-up: median [range] LVMI: 95.2 [66.0–184.0] g/m^2<br>• $p = 0.55$

Author, year	*N*	Treatment duration<br>*Assessment method*	Key results
Germain 2016 [16], Germain 2019 [17]	50	6 months plus 6- to 12-month open-label extension plus an additional year<br><br>*Echocardiography*	• FACETS study (NCT00985301) At follow-up, change in LVMI from baseline to month 18 or 24, g/m^2, mean (SEM; 95% CI)<br>○ Patients with suitable mutant α-galactosidase (*n* = 27): −7.7 (3.7; −15.4, −0.01) (change from baseline considered significant because 95% CI does not include zero)<br>○ Patients with suitable variant *GLA* and LVH at baseline (*n* = 8): −18.6 (8.3; −38.2, 1.0)<br>• LVMI at 6 months, g/m^2, mean (SD)<br>○ Migalastat-migalastat (*n* = 27): 92.9 (29)<br>○ Placebo-migalastat (*n* = 16): 108 (51)<br>• LVMI, g/m^2, mean (SD)<br>○ Baseline (*n* = 44): 96.5 (33)<br>○ Month 24 (*n* = 15): 87.1 (28)<br>○ Annualized rate of change from baseline (*n* = 14): −3.05 (8.9; 95% CI, 20.4, 9.62)<br>• The changes in the LVMI correlated with changes in the intraventricular septum thickness (R^2 = 0.26; *p* = 0.006) but not with changes in the LV PWT (R^2 = 0.06; *p* = 0.23).<br>• Change from baseline to month 24 in LVMI, g/m^2, mean (SD; 95% CI)<br>○ Classic phenotype (*n* = 9): −16.7 (18.64; −31.1, −2.4)<br>• Other patients (*n* = 18): −3.2 (18.66; −12.5, 6.1)
Giugliani 2013 [18]	9	48 weeks<br><br>*MRI, ECG and echocardiography*	• Phase 2 open-label study in females<br>• From baseline to week 48, three patients had a decline of ≥ 10% from baseline in LVMI. Two of these had an amenable mutation<br>• LVMI, range, g/m^2<br>○ Week 24<br>– With amendable *GLA* mutations (*n* = 2): 61.2, 72.7<br>– With non-amendable *GLA* mutations (*n* = 2): 66.2, 71.7<br>○ Week 48<br>– With amendable *GLA* mutations (*n* = 5): 50.3, 105.1<br>– With non-amendable *GLA* mutations (*n* = 4): 68.3, 103.2
Hopkin 2022* [19]	97	Median [IQR] years: 5.1 [2.3, 6.8]<br><br>*Echocardiography*	• An integrated analysis of FACETS (NCT00925301, ERT-naïve), and the active-controlled ATTRACT (NCT01218659, ERT-experienced) and open-label extension studies<br>• In the phase 3 FACETS and ATTRACT trials, following LVMI reduction, LVMI remained stable with continuing migalastat in the OLE (*N* = 84). Mean (SD) [range] migalastat exposure (years): 2.7 (1.0) [0.1, 4.3]
Lenders 2020 [20]	59	≤ 12 months<br><br>*Echocardiography*	• FAMOUS study (NCT03135197) documented long-term treatment with migalastat under 'real-world' conditions<br>• Overall, mean (SD) LVMI (g/m^2) as primary endpoint decreased significantly from baseline to 12 months<br>○ Overall difference 10.2 (95% CI 5.3, 15.2) (108.6 [48.0] vs 98.4 [41.4]; *p* = 0.001)<br>○ In females, difference 7.2 (95% CI 2.4, 11.9) (86.2 [18.7] vs 79.0 [21.0]; *p* = 0.0050; reference values, 43–95)<br>– 6/10 (60%) females with increased LVMI at baseline showed a reduction of LVMI over time<br>– 13/16 (81.3%) females with normal LVMI at baseline also showed a reduction<br>○ In males, LVMI significantly decreased by 13.7 (95% CI 4.3, 23.1) (134.0 [57.9] vs 120.3 [47.9]; *p* = 0.0061; reference values, 49–115)<br>– 11/12 (91.7%) males with increased LVMI at baseline showed a reduction of LVMI over time<br>9/11 (81.8%) males with normal LVMI at baseline also showed a reduction
Lenders 2021 [21]	59	24 months<br><br>*Echocardiography*	• FAMOUS study (NCT03135197; see row above for 12-month data) including expanded analysis population<br>• Overall, mean (SD) LVMI (g/m^2) as primary endpoint decreased significantly from baseline<br>○ 10.4 (17.1) difference at 12 months (109.5 [47.3] vs 99.0 [41.4]; *p* = 0.0004)<br>○ 7.5 (17.4) difference at 24 months (109.5 [47.3] vs 102.0 [40.0]; *p* = 0.0118)

Author, year	*N*	Treatment duration<br>*Assessment method*	Key results
			○ In females, (mean [SD]) LVMI decreased from baseline to month 12 (8.8 [11.5]) g/m^2 (86.2 [18.3] vs 77.4 [19.7]; *p* = 0.0042) and from baseline to month 24 (4.6 [9.1]) g/m^2 (86.2 [18.3] vs 81.6 [21.3]; *p* = 0.0554)<br>• Female patients, LVMI (g/m^2)<br>○ Overall: significant loss of LVMI of −11.9% (95% CI: −16.3, −0.8; *p* = 0.0033) within 12 months and −7.5% (95% CI: −11.1, −0.8; *p* = 0.0195) within 24 months<br>○ Non-hypertrophic females (LVMI reference range: 43–95 g/m^2) presented with a significant reduction in LVMI after 12 months (−6.0 [8.8] g/m^2; *p* = 0.0350), which was slightly reduced to −4.8 (6.5) g/m^2 after 24 months (*p* = 0.0541)<br>○ Hypertrophic females presented with a significant reduction in (mean [SD]) LVMI after 12 months (−13.4 [14.3] g/m^2; *p* = 0.0163) but not after 24 months (−4.4 [12.7] g/m^2; *p* = 0.3309) of follow-up from baseline to month 24<br>• Male patients, LVMI (g/m^2)<br>○ Overall: significant reduction in LVMI by −10.2% (95% CI: −18.4, −3.4; *p* = 0.0035) within 12 months and stable values −8.4% (95% CI: −13.5, 2.8; *p* = 0.1780) within 24 months<br>○ Non-hypertrophic males (LVMI reference range: 49–115 g/m^2): presented with stable (mean [SD]) LVMI after 12 months (−5.5 [21.1] g/m^2; *p* = 0.5831) and 24 months (−2.6 [17.5] g/m^2; *p* = 0.8312)<br>○ Hypertrophic (mean [SD]) LVMI decreased after 12 months (−18.2 [19.8] g/m^2; *p* = 0.0163) and 24 months (−17.3 [24.7] g/m^2; *p* = 0.0595)<br>• Mean (SD) LVMI reduction from baseline (g/m^2) in ERT-naïve patients<br>○ 12 months: −10.9 (18.6); *p* = 0.0201<br>○ 24 months: −6.8 (15.9); *p* = 0.0963<br>• Mean (SD) LVMI reduction from baseline (g/m^2) in ERT-exposed patients<br>○ 12 months: −9.9 (16.2); *p* = 0.0028<br>24 months: −7.5 (18.2); *p* = 0.0425
Orsborne 2020* [22]	78	ERT and migalastat<br><br>Median [range] follow-up: 594 [235, 791] days<br><br>*Echocardiography*	• Study included 43 patients who switched from ERT (33 were treatment-naïve)<br>• Echocardiography pre-chaperone therapy demonstrated elevated indexed LVM in 29 individuals (5 mild, 8 moderate and 17 severe)<br>• LVMI was 118.9 g/m^2 at baseline<br>• Mean indexed LVM declined from 118.9 to 115.8 g/m^2. Reduction in LVM was evenly accounted for by both the septum and the posterior wall of the left ventricle<br>• In the 10 patients who have had 2-year follow-up tests, cardiac parameters appeared stable (two individuals switched back to ERT following a perceived deterioration in their cardiac status)
Ramaswami 2022 [23]	21	1 year<br><br>*Echocardiography*	• The ASPIRE study assessed treatment in adolescents with FD (12 to < 18 years, ≥ 45 kg)<br>• Overall mean change (SD) from baseline for LVMI (*n* = 18) was −3.9 (13.53) g/m^2 (not statistically significant)
Skuban 2017 [24]<br><br>(Long-term follow-up reported by Hopkin *et al.* [above])	FACETS, 67<br>ATTRACT, 57	FACETS: 24 months<br><br>ATTRACT: 18 months plus 12-month OLE<br><br>*Echocardiography*	• Two randomized phase 3 clinical studies (FACETS [NCT00985301]; ATTRACT [NCT01218659])<br>• FACETS: mean change from baseline in LVMI was −7.7 g/m^2 (95% CI: −15.4, −0.009; *p* < 0.05)<br>• ATTRACT: LVMI was significantly decreased with migalastat, with an average change from baseline over 18 months of −6.6 g/m^2 (95% CI: −11.0, −2.2); there was no significant change with ERT
Torra 2018* [25]	NR	FACETS: 24 months<br>ATTRACT: 18 months	• FACETS: migalastat > migalastat (0–24 months):<br>○ Baseline eGFR 30–60 mL/min/1.73 m²: LVMI mean change (SD): −5.5 (14.2)<br>○ Baseline eGFR 30 to ≥60 mL/min/1.73 m²: LVMI mean change (SD): −9.2 (21.6)<br>• FACETS: placebo > migalastat (6–24 months):

Author, year	*N*	Treatment duration *Assessment method*	Key results
			○ Baseline eGFR 30–60 mL/min/1.73 m²: LVMI mean change (SD): −21.0 (20.2)<br>○ Baseline eGFR 30 to ≥60 mL/min/1.73 m²: LVMI mean change (SD): −3.2 (17.7)<br>• ATTRACT:<br>○ Baseline eGFR 30–60 mL/min/1.73 m²: LVMI mean change (SD): −10.2 (3.4)<br>○ Baseline eGFR 30 to ≥60 mL/min/1.73 m²: LVMI mean change (SD): −4.8 (11.2)
**Pegunigalsidase alfa single-arm studies**			
Hughes 2023b [29]	16	12 months followed by 60 month open-label extension<br><br>*MRI*	• LVMI at 60 months<br>○ Increase in females: 13.6 g/m^2 (SE: 5.3)<br>○ Increase in males: 5.7 g/m^2 (SE: 2.2)<br>○ Statistical analyses not reported
Schiffmann 2019 [30]	16	3-month dose-ranging study, followed by a 9-month extension<br><br>*Echocardiography*	• At baseline, mean LVM and LVMI were within the normal range, and no cardiac fibrosis was present<br>• Cardiac MRI results showed that the majority of patients maintained cardiac parameters (LVM, LVMI and EF) within the normal ranges throughout the 12-month study, with a slight decrease in LVMI in patients with classic phenotype FD

*Congress abstract.

CI, confidence interval. CKD, chronic kidney disease. CV, cardiovascular. ECG, electrocardiogram. Edf, empirical distribution function. EF, ejection fraction. ERT, enzyme replacement therapy. ESRD, end-stage renal disease. FD, Fabry, disease. HR, hazard ratio. IQR, interquartile range. ITT, intention-to-treat. LVH, left ventricular hypertrophy. LVM, left ventricular mass. LVMI, left ventricular mass index. MRI, magnetic resonance imaging. NA, not available. NR, not reported. PWT, posterior wall thickness. SD, standard deviation. SE, standard error.

B

Author, year	Switch of treatment, *n*	Treatment duration *Assessment method*	Key results
**Switch studies**			
Goker-Alpan 2015 [32]	From agalsidase beta to agalsidase alfa, 71	Up to 24 months<br><br>*Echocardiography*	• Cohort studied included patients who were naïve to ERT or switched from agalsidase beta<br>• Mean LVMI did not change significantly up to 24 months in either agalsidase alfa naive or switch groups<br>• Mean LVMI change from baseline did not change significantly in patients with or without LVH at baseline<br>• [Cardiac data presented in figures]
Hughes 2017 [34], Narita 2020 [35]	Migalastat, 36<br>ERT, 24<br><br>Japanese population<br>• Migalastat, 5<br>• ERT, 1	*18 months*<br><br>*30 months*<br><br>*Echocardiography*	• Patients were previously treated with ERT who switched to migalastat<br>• Mean (95% CI) LVMI change from baseline to 18 months, g/m^2<br>○ All patients: migalastat (n = 33): −6.6 (−11.0, −2.2), p reported to be significant (NR)<br>○ Patients who continued ERT (n = 16): −2.2 (−11.0, 7.0)<br>○ Japanese patients (n = 5): −13.8 (change in LVMI in Japanese patients who received migalastat was greater than in the overall population)<br>• Baseline to 30 and 42 months<br>○ In Japanese patients treated with migalastat (n = 5), decrease in LVMI persisted over 30 months and the change from baseline at 42 months was −27.2 g/m^2

Author, year	Switch of treatment, *n*	Treatment duration<br>*Assessment method*	Key results
Linhart 2023 [33]	From agalsidase alfa to pegunigalsidase alfa, 22	12 months<br><br>*MRI*	• Males (*n* = 15): baseline LVMI: 97.6 (8.9) g/m²; 12 months: 98.3 (7.8) g/m²<br>• Females (*n* = 7): baseline LMVI: 66.9 (5.8) g/m²; 12 months: 74.1 (7.2) g/m²<br>• Mean change: males: 2.4 (3.4) g/m² (*p* = 0.50); females: 7.1 (5.0) g/m² (*p* = 0.21)
Müntze 2019 [39], Muentze 2018 [40]	To migalastat from prior ERT or previously treatment naïve, 21	1 year<br><br>*Echocardiography*	• Cardiac and extracardiac effects of migalastat oral therapy in patients were assessed in a prospective monocentric register<br>• Patients were either treatment-naïve or switched from a prior ERT to prevent further intravenous administration<br>• Median [IQR] myocardial mass index (g/m^2) at baseline<br>o Total (*n* = 13): 137 [86, 159]<br>o Switch (*n* = 6): 127 [6.5, 176.5]<br>o Naïve (*n* = 7): 154 [82, 158]<br>• Median [IQR] myocardial mass index (g/m^2) at 3–6 months<br>o Total (*n* = 13): 112 [71, 157.5];<br>o Switch (*n* = 6): 100.5 [66, 169.3]<br>• Median [IQR] myocardial mass index (g/m^2) at 12 months<br>o Total (*n* = 13): 130 [82, 169]; *p* = 0.037 vs baseline<br>o Naïve (*n* = 7): 130 [70, 184]<br>o Switch (*n* = 6): 126.5 [84, 170]<br>• Myocardial mass index after 12 months was reduced by 6.7% (*n* = 6)
Riccio 2020 [41]	From ERT to migalastat, 7	12 months<br><br>*Echocardiography*	• Mean (SD) LVMI in male patients, mm<br>o Baseline: 39.58 (12.30)12 months ERT: 39.71 (9.93)12 months migalastat: 37.17 (11.09) (*p* = 0.016 vs baseline; *p* = 0.028 vs ERT)

*Congress abstract.

CI, confidence interval. ERT, enzyme replacement therapy. IQR, interquartile range. LVMI, left ventricular mass index. MRI, magnetic resonance imaging. NR, not reported. NS, not significant.

Supplementary Table S7

Overview of data for other structural cardiovascular parameters from (A) single-arm studies, (B) comparator and (C) switch studies.

A

**Author, year**	***N***	**Treatment duration**	**Key results**
**Agalsidase alfa single-arm studies**			
Beck 2004 [87]	545	Mean: 17 months; maximum: 56 months	• Overall, 1 and 2 years of treatment with agalsidase alfa (*n* = 188 and *n* = 92, respectively) was associated with a significant ($p < 0.05$) decrease in MWT and LVM in patients followed longitudinally<br>• Individual data from serial echocardiographic examinations of patients with LVH (MWT > 11 mm and LVM > 50 g/$m^{2.7}$) indicated that decreased MWT and height-adjusted LVM was most notable in those patients with the greatest degree of hypertrophy
Goker-Alpan 2016 [47]	14	Median [range]): 54.5 [54.0, 59.0] weeks	• Midwall fractional shortening remained within the normal range in children following treatment<br>• Overall change from baseline by week 55<br>o Mean [95% CI], −0.62% [−2.69%, 1.46%]<br>o Median [range], −1.27 [−6.1, 4.6]
Hughes 2011 [48]	250	≥ 4 years	• Evaluation of relative effectiveness of ERT in men and women (male: *n*/*N* [%], 172/250 [68.8%])<br>• Mean (SD) midwall fractional shortening (%) at 4 years<br>o Females (*n* = 12): 16.2 (4.0); *p* = 0.586 vs baseline<br>o Males (*n* = 17): 16.3 (3.3); *p* = 0.375 vs baseline
Kampmann 2015 [50]	45	Median [range]: 10.8 [9.6, 12.5] years	• After 10 years of treatment, MWT was significantly reduced in males (LS mean [95% CI] change: −1.89 [−2.58, −1.19] mm; $p < 0.0001$)<br>• Changes were apparent after 1 year (LS mean [95% CI] change: −2.08 [−2.69, −1.46] mm; $p < 0.0001$)<br>• Statistically significant changes were also apparent after 1 year in females (LS mean [95 % CI] change: −2.01 [−2.55, −1.47] mm; $p < 0.0001$)<br>• After 10 years, MWT was not significantly different from before treatment in females (LS mean [95% CI] change: −0.48 [−1.05, 0.09] mm; *p* = 0.0999)<br>• Mean (SD) LVEF (%) after ~10 years of ERT: females, 68.4 (6.9); males, 69.8 (7.0)<br>o Mean [95% CI] LVEF (%) change from baseline: females, −3.64 [−6.74, −0.54]; *p* = 0.022; males, 1.31 [−2.16, 4.78]; *p* = 0.4546
Ramaswami 2019 [88]	69	Median [range] 13.6 [10.1, 17.1] years	• Long-term study of patients who had received agalsidase alfa ERT over a 10-year period<br>• Mean [95% CI] PWTd slope (mm/year) in patients in eGFR category < 60 mL/min/1.73 m^2 at baseline<br>o Females (*n* = 6): 0.01 [−0.12, 0.15]<br>o Males (*n* = 4): 0.03 [−0.11, 0.18]<br>• Mean [95% CI] PWTd slope (mm/year) in patients in eGFR category ≥ 60 mL/min/1.73 m^2 at baseline<br>o Females (*n* = 24): 0.10 [0.01, 0.18]<br>o Males (*n* = 23): 0.11 [−0.03, 0.19]
Tsuboi 2017 [89]	36	Median [range]: 62.5 [8, 84] months	• Cohort of patients with FD in Japan including both ERT treatment-naïve and -experienced patients<br>• LVPWT: 10.5 mm at baseline; 9.9 mm at 60 months
**Agalsidase beta single-arm studies**			
Burlina 2019 [90]	49	≥ 2.5 years	• Fabry Registry analysis<br>• Among patients started on agalsidase beta when aged 5–30 years<br>• Z-scores of LVPWT remained stable over time (slopes: older males (*n* = 14), −0.10 mm/year; younger males (*n* = 13), −0.08 mm/year). Slope estimates were unreliable among females

Author, year	N	Treatment duration	Key results
			• Z-scores of IVST remained stable over time (slopes: older males (*n* = 14), −0.07 mm/year; younger males (*n* = 13), −1.11 mm/year). Slope estimates were unreliable among females
Elliot 2006 [91]	10	Mean (SD): 10.1 (2.3) months	• Male patients (*n* = 5) receiving 1 or 2 mg/kg agalsidase beta<br>• At follow-up, mean (SD); *p* value vs baseline<br>o LV cavity mean change in end-systolic cavity dimension: 0.1 (0.3) mm; *p* = NS<br>o LV cavity mean change in end-diastolic cavity dimension: 0.1 (0.2) mm; *p* = NS<br>o Fractional shortening: 31.4% (6.3%); *p* = 0.08<br>o Maximum LV wall thickness mean change: 0.02 (0.3) mm; *p* = NS
Germain 2015 [92]	52	Median [IQR]: 10 [7.3, 10.3] years	• Analysis of phase 3 clinical trial data and Fabry Registry observational analysis<br>• LVPWT: mean slopes: LRI: +0.08 mm/year), HRI: +0.10 mm/year (*p* = 0.282)<br>• IVST: mean slopes: LRI: +0.04 mm/year (*p* = 0.536), HRI: +0.14 mm/year (*p* = 0.068)
Hwang 2022 [62]	10	22 weeks	• Evaluation of a recombinant agalsidase beta<br>• Mean (SD)<br>o LVPWTd at week 22: 11.77 (5.27) mm; change from baseline, −0.99 (2.18) mm; *p* = 0.625<br>o IVSD at week 22: 9.66 (4.70) mm; change from baseline, −0.27 (1.65) mm; *p* = 0.906<br>o LVID at week 22: 48.99 (4.61) mm; change from baseline, 2.02 (9.10) mm; *p* = 0.945<br>o LVEF at week 22: 68.20 (6.00) g/m^2; change from baseline, 0.98 (6.40) g/m^2; *p* = 0.555
Imbriaco 2009 [93]	11	Mean (SD) [range]: 45 (9) [29, 58] months	• Before the start of agalsidase beta therapy: range of LV wall thickness, 11, 22 mm<br>• After mean (SD) agalsidase beta treatment duration of 45 (9)] months: range of LV wall thickness, 10, 22 mm
Kalliokoski 2006 [94]	10	12 months	• Cardiac structure measures before and after 12 months of treatment of patients (*n* = 7) in Finland, mean (SD)<br>o LV systolic diameter (mm): before, 32.1 (3.2); after, 32.3 (2.9); *p* = 0.94<br>o LV diastolic diameter (mm): before, 53.4 (2.2); after, 53.7 (1.9); *p* = 0.89<br>o PW systolic thickness (mm): before, 17.7 (1.9); after, 18.0 (1.8); *p* = 0.69<br>o PW diastolic thickness (mm): before, 10.3 (1.1); after, 10.0 (1.0); *p* = 0.63<br>o SWTs (mm): before, 15.7 (0.9); after, 17.3 (1.7); *p* = 0.19<br>o SWTd (mm): before, 10.9 (1.6); after, 12.1 (2.1); *p* = 0.25<br>o Relative wall thickness: before, 0.69 (0.18); after, 0.70 (0.14); *p* = 0.66<br>o EF (%): before, 69 (17); after, 68 (17); *p* = 0.86
Messalli 2012 [95]	16	48 months	• Study specifically on cardiac structures (MRI study)<br>• Following ERT in patients with FD in Italy, mean (SD)<br>o LVPWT, maximum measurable wall thickness (mm): 13 (3); *p* < 0.001 vs baseline<br>o IVST (mm: 67 (7); *p* < 0.001 vs baseline<br>o Apex: 63 (6) *p* < 0.001 vs baseline<br>o Lateral wall (mm: 63 (10); *p* < 0.01 vs baseline
Motwani 2012 [96]	66	Median [range]: 3 [2, 5 years]	• Study of the effects of ERT on cardiac manifestations and to explore baseline features or subgroupings that could predict LVH regression with ERT in ERT-naïve patients<br>• The overall mean (SD) MWT was significantly reduced by ERT at follow-up: 14 (6) vs 13 (5) mm; both *p* values < 0.001<br>o On subgroup analysis, this improvement was also seen in both sexes and in both mild and moderate/severe disease groups (all *p* values < 0.05)<br>• LVH status<br>o LVH at baseline (*n* = 42):mean (SD) MWT and LVEDD were significantly reduced by ERT,MWT: 17 (6) vs 16 (5) mm; LVEDD: 55 (6) vs 54 (6) mm; both *p* values < 0.05.<br>o t No LVH at baseline (*n* = 24): MWT: 17 (1) vs 16 (1) mm; LVEDD: 49 (5) vs 49 (4) mm; both *p* values > 0.05<br>• Ascending aorta diameter

Author, year	*N*	Treatment duration	Key results
			○ 16 patients had dilatation of the ascending aorta (14 male; 3 female) prior to ERT, and all cases remained dilated at the end of follow-up. When present, dilatation was in the absence of hypertension or aortic valve disease<br>○ There was no significant change in mean (SD) aortic dimension,32 (5) vs 32 (5) mm; $p = 0.23$<br>○ This was true for both sexes and irrespective of baseline FOS-MSSI or LVH status on subgroup analysis (all *p* values > 0.05)<br>• EF<br>○ The mean (SD) EF was within normal range at baseline but still improved at the end of follow-up, 63 (4) vs 64 (3)%; p < 0.01)<br>○ On subgroup analysis, this improvement was also seen in both sexes and in both mild and moderate/severe disease groups (all *p* values < 0.05)<br>○ Mean (SD) EF also improved in the LVH subgroup,62 (5) vs 64 (3)%; $p < 0.001$, but there was no significant change in the non-LVH group,64 (2) vs 64 (4)%
Ortiz 2021* [97]	254	1.1 years pre-ERT and 4.1 years post-ERT	• Fabry Registry observational analysis<br>• Pre-/post-study of cardiomyopathy outcomes in male patients with the classic FD<br>• LVPWTd (mm/year) ($n = 87$) (median age at start of ERT, 35.1 years)<br>○ Pre-treatment: increased (slope = +0.33 mm/year; $p = 0.01$)<br>○ Post-treatment: stabilized (slope = −0.09 mm/year; $p > 0.05$)<br>○ $p_{pre\text{-}post} < 0.01$<br>• IVSTd (mm/year) ($n = 73$) (median age at start of ERT, 35.1 years)<br>○ Pre-treatment: increased (slope = +0.02 mm/year; $p > 0.05$)<br>○ Post-treatment: stabilized (slope = −0.02 mm/year; $p > 0.05$)<br>○ $p_{pre\text{-}post} = 0.83$<br>• Overall, patients with LRI had more stable cardiac ultrasound indices than those with HRI throughout follow-up
Ramaswami 2019 [64]	31	5 years	• Male paediatric patients with FD receiving agalsidase beta (0.5 mg/kg q2w [n =16] or 1.0 mg/kg q4w [n =15]). Results were not analysed according to treatment regimen<br>• MWT, mean, median (SD) [range], cm<br>○ Year 5 overall ($n = 21$): 0.8, 0.8 (0.1) [0.6, 1.1]<br>○ Change from baseline: 0, 0 (0) [−0.2, 0.2]; $p = 0.2814$<br>○ % change from baseline: 3.3, 2.8 (10.1) [−16.4, 25.3]
Wanner 2020 [98]	42	3.7 (0.9) [2.0, 4.9]	• Observational study of female patients from the Fabry Registry<br>• Estimated LVPWT group slope (95% CI) ($n = 38$):<br>○ Pre-treatment: 0.28 (0.10, 0.46) mm/year; p <0.01<br>○ Post-treatment: −0.13 (−0.30, 0.04) mm/year; $p = 0.13$<br>○ Pretreatment vs. post-treatment slope difference = −0.41 (−0.68, −0.15) mm/year; *p* for pretreatment vs. post-treatment slope difference <0.01<br>• Estimated IVST group slope (95% CI) ($n = 38$):<br>○ Pre-treatment: 0.33 (0.12, 0.54) (p <0.01) mm/year; p <0.01<br>○ Post-treatment: 0.01 (−0.20, 0.21) mm/year; $p = 0.93$<br>○ Pretreatment vs. post-treatment slope difference = −0.32 (−0.67, 0.02) mm/year; *p* for pretreatment vs. post-treatment slope difference = 0.07<br>• Pre-treatment vs post-treatment LVPWT slope difference (95% CI) stratified by HRI vs LRI, or use of ACEI/ARBs<br>○ HRI ($n = 9$): −0.92 (−1.36, −0.49) mm/year; *p* (pre–post difference) < 0.01; *p* (interaction) = 0.09<br>○ LRI ($n = 21$): −0.35 (−0.75, 0.04) mm/year; *p* (pre–post difference) = 0.08<br>○ 'Ever' ACEI/ARBs ($n = 26$): −0.53 (−0.89, −0.17) mm/year; *p* (pre–post difference) < 0.01; *p* (interaction) = 0.24<br>○ 'Never' ACEI/ARBs ($n = 12$): −0.10 (−0.50, 0.30) mm/year; *p* (pre–post difference) = 0.62<br>• Pre-treatment vs post-treatment IVST slope difference (95% CI) stratified by HRI vs LRI, or use of ACEI/ARBs

Author, year	N	Treatment duration	Key results
			○ HRI (n = 10): −1.03 (−1.63, −0.42) mm/year; p (pre–post difference) < 0.01; p (interaction) < 0.05<br>○ LRI (n = 20): −0.12 (−0.67, 0.42) mm/year; p (pre–post difference) = 0.66<br>○ 'Ever' ACEI/ARBs (n = 25): −0.45 (−0.89, −0.00) mm/year; p (pre–post difference) < 0.05; p (interaction) = 0.30<br>○ 'Never' ACEI/ARBs (n = 13): −0.17 (−0.72, 0.39) mm/year; p (pre–post difference) = 0.54
Weidemann 2013 [67]	40	Median [IQR]: 6.0 [5.1, 7.2] years	• Observational study of patients with genetically proven FD from the Fabry Registry<br>• Mean (SD) data at follow-up; p value compared with baseline<br>○ PWT (mm): 11.4 (2.1); $p < 0.0001$<br>○ Septum thickness (mm): 11.9 (1.8); $p < 0.0001$<br>○ EF (%): 63 (7); $p = 0.692$<br>○ LVEDD (mm): 49 (6); $p = 0.883$<br>• Fibrosis<br>○ Patients with fibrosis (n): 31; $p = 0.797$<br>○ Patients with new fibrosis (n): 2<br>○ Mean (SD) fibrosis in relation to LV (%): 3.7 (4.0); $p < 0.0001$<br>○ Patients with new VT (n): 12
Wuest 2011 [99]	14	Mean (SD): 13 (1) months	• Baseline, mean (SD)<br>○ Normalized RV values: RV mass, 31 (6) g/m^2; EDV, 88 (13) mL/m^2; ESV, 39 (9) mL/m^2; SV, 49 (7) mL/m^2; EF, 56% (5%)<br>○ Normalized LV values: LVM, 102 (26) g/m^2; EDV, 76 (13) mL/m^2; ESV, 29 (9) mL/m^2; SV, 48 (7) mL/ m^2; EF, 64% (7%)<br>• At follow-up, mean (SD), p value compared with baseline<br>○ Normalized RV values: RV mass: 27 (7) g/m^2 ($p < 0.05$); EDV, 76 (24) mL/m^2 ($p < 0.05$); ESV, 33 (13) mL/m^2; SV, 43 (12) mL/m^2; RVEF: 57% (7%)<br>○ Normalized LV values: LVM, 94 (27) g/m^2 ($p < 0.05$); EDV, 66 (22) mL/m^2 ($p < 0.05$); ESV, 23 (9) mL/m^2 ($p < 0.05$); SV, 43 (14) mL/m^2; LVEF: 66% (5%) ($p < 0.05$)
**Mixed or non-specified ERT single-arm studies**			
Hongo 2018 [79]	Agalsidase alfa or agalsidase beta, 42	Median [IQR]: 10 [6.75, 11] years	• At baseline<br>○ End-diastolic diameter: male vs female, $p < 0.01$<br>○ End-systolic diameter: male vs female, $p < 0.01$<br>• PW (mm), mean (SD)<br>○ Male patients (year: 0, 1, 2, 3, 4, 5, 6, 7, 8, 9, 10, 11)<br>– 9.8 (1.7), 10.2 (1.6), 10.3 (2.1), 10.6 (1.5), 10.1 (1.5), 10.6 (2.4), 10.5 (1.5), 10.5 (1.6), 10.9 (1.9), 11.8 (2.2), 10.9 (2.2), 11.5 (2.2)<br>– $p < 0.05$ for year 8 vs year 0<br>– N: 17, 14, 13, 12, 12, 13, 15, 14, 12, 11, 14, 12<br>○ Female patients (year: 0, 1, 2, 3, 4, 5, 6, 7, 8)<br>– 9.0 (2.1), 9.4 (2.3), 9.6 (2.3), 9.2 (2.6), 9.3 (2.2), 9.3 (2.1), 9.5 (2.1), 9.9 (2.6), 9.8 (2.8)<br>– N: 25, 22, 22, 21, 19, 17, 16, 14, 12<br>○ Male patients with FD without excessive LVH (year: 0, 1, 2, 3, 4, 5, 6, 7, 8, 9, 10, 11)<br>– 9.3 (1.4), 9.8 (1.4), 9.5 (1.7), 10.0 (1.3), 9.7 (1.2), 9.7 (1.4), 10.2 (1.4), 10.0 (1.2), 10.4 (1.6), 11.1 (1.8), 10.3 (1.7), 10.7 (1.4)<br>– N: 14, 11, 10, 9, 10, 10, 13, 12, 10, 9, 12, 10<br>• IVST (mm), mean (SD)<br>○ Male patients (year: 0, 1, 2, 3, 4, 5, 6, 7, 8, 9, 10, 11)<br>– 9.8 (2.0), 10.1 (2.0), 10.6 (2.3), 10.5 (2.0), 10.0 (1.4), 10.2 (2.4), 10.5 (2.1), 10.3 (2.0), 11.1 (3.5), 11.3 (2.2), 11.2 (2.4), 11.6 (2.3)

Author, year	*N*	Treatment duration	Key results
			– *N*: 17, 14, 13, 12, 12, 13, 15, 14, 12, 11, 14, 12<br>○ Female patients (year: 0, 1, 2, 3, 4, 5, 6, 7, 8)<br>– 9.0 (2.2), 9.4 (2.5), 10.1 (2.9), 9.7 (3.0), 9.9 (2.6), 9.7 (3.2), 10.1 (2.6), 9.8 (2.5), 10.3 (3.8)<br>– *N*: 25, 22, 22, 21, 19, 17, 16, 14, 12<br>○ Male patients with FD without excessive LVH (year: 0, 1, 2, 3, 4, 5, 6, 7, 8, 9, 10, 11)<br>– 9.2 (1.8), 9.4 (1.5), 9.7 (1.4), 9.7 (1.5), 9.6 (1.1), 9.3 (1.6), 10.0 (1.6), 9.7 (1.3), 10.1 (1.8), 10.7 (1.9), 10.6 (2.0), 10.8 (1.7)<br>– *N*: 14, 11, 10, 9, 10, 10, 13, 12, 10, 9, 12, 10<br>• EF (%), mean (SD)<br>○ Male patients (year: 0, 1, 2, 3, 4, 5, 6, 7, 8, 9, 10, 11)<br>– 73.7 (3.7), 75.9 (3.6), 75.1 (5.2), 76.8 (4.9), 77.1 (5.6), 77.1 (4.7), 76.5 (3.6), 74.1 (5.5), 75.3 (6.8), 75.3 (6.8), 72.3 (4.2), 73.7 (5.3)<br>– $p < 0.05$ year 5 and year 6 vs year 0<br>– *N*: 17, 14, 13, 12, 12, 13, 15, 14, 12, 11, 14, 12<br>○ Female patients (year: 0, 1, 2, 3, 4, 5, 6, 7, 8)<br>– 75.6 (5.1), 74.7 (5.4), 73.1 (5.5), 73.9 (4.8), 75.0 (6.0), 73.6 (5.1), 73.1 (4.5), 73.7 (7.2), 72.3 (6.8)<br>– *N*: 25, 22, 22, 21, 19, 17, 16, 14, 12<br>○ Male patients with FD without excessive LVH (year: 0, 1, 2, 3, 4, 5, 6, 7, 8, 9, 10, 11)<br>– 73.6 (3.5), 76.3 (3.7), 75.8 (5.8), 77.7 (4.6), 77.5 (6.1), 77.5 (3.4), 76.6 (3.9), 73.7 (5.7), 76.4 (6.9), 76.4 (7.1), 72.7 (4.4), 74.1 (5.7)<br>– *N*: 14, 11, 10, 9, 10, 10, 13, 12, 10, 9, 12, 10<br>• End-diastolic dimension (mm), mean (SD)<br>○ Male patients (year: 0, 1, 2, 3, 4, 5, 6, 7, 8, 9, 10, 11)<br>– 50.5 (3.9), 48.9 (4.1), 48.8 (4.0), 48.7 (4.2), 50.0 (3.1), 49.4 (3.4), 49.6 (3.2), 50.0 (3.3), 48.7 (3.4), 50.3 (2.3), 50.9 (3.3), 50.3 (2.8)<br>○ Female patients (year 0, 1, 2, 3, 4, 5, 6, 7, 8)<br>– 43.7 (2.8), 43.3 (2.4), 43.4 (3.0), 43.7 (2.4), 43.9 (3.0), 43.5 (3.5), 44.2 (4.0), 43.4 (3.9), 43.8 (5.0)<br>○ Male patients with FD without excessive LVH (year: 0, 1, 2, 3, 4, 5, 6, 7, 8, 9, 10, 11)<br>– 50.4 (4.2), 48.9 (4.2), 48.5 (4.0), 48.6 (4.4), 50.2 (3.4), 48.7 (2.9), 50.0 (3.3), 49.9 (3.3), 48.7 (3.4), 50.2 (2.2), 50.9 (3.3), 50.4 (2.5)<br>• End-systolic dimension (mm), mean (SD)<br>○ Male patients (year: 0, 1, 2, 3, 4, 5, 6, 7, 8, 9, 10, 11)<br>– 32.1 (3.4), 30.0 (3.1), 29.0 (3.1), 29.1 (3.2), 30.4 (3.2), 29.3 (1.8), 29.8 (2.9), 31.0 (2.5), 29.3 (2.1), 30.2 (2.9), 30.8 (1.8), 30.3 (2.7)<br>– *N*: 17, 14, 13, 12, 12, 13, 15, 14, 12, 11, 14, 12<br>○ Female patients (year: 0, 1, 2, 3, 4, 5, 6, 7, 8)<br>– 26.8 (2.7), 26.5 (3.1), 26.5 (2.9), 26.9 (2.6), 26.5 (3.5), 26.8 (3.3), 27.0 (3.3), 27.1 (4.1), 27.2 (5.0)<br>– *N*: 25, 22, 22, 21, 19, 17, 16, 14, 12<br>○ Male patients with FD without excessive LVH (year: 0, 1, 2, 3, 4, 5, 6, 7, 8, 9, 10, 11)<br>– 32 (3.5), 29.7 (3.3), 29.1 (3.3), 28.4 (2.4), 30.4 (3.5), 29.4 (1.7), 30.1 (2.9), 31.0 (2.4), 29.3 (2.3), 29.7 (2.7), 30.8 (1.9), 30.4 (2.9)<br>– *N*: 14, 11, 10, 9, 10, 10, 13, 12, 10, 9, 12, 10
Kovacevic-Preradovic 2008 [80]	Agalsidase alfa, 22<br>Agalsidase beta, 5	Median: 37 months	• LVWT (*n*): 10 (34%); $p = 1.0$ vs baseline<br>• Mean (SD); *p* value vs baseline:<br>○ IVST (mm): 12.5 (5); $p = 0.009$<br>○ PWT (mm): 10.3 (3.1); $p = 0.21$<br>○ LV end-diastolic dimension index (mm/m^2): 27 (3); $p = 0.18$

Author, year	N	Treatment duration	Key results
	Switch from agalsidase alfa to agalsidase beta, 2		○ Relative wall index: 0.46 (0.16); *p* = 0.35<br>○ Left atrium end-systolic dimensions index: 20 (3); *p* = 0.36
Lenders 2016 [81]	Agalsidase alfa or agalsidase beta, 76	NR	• Patients with ERT inhibition showed a trend toward higher relative wall thickness vs those without inhibition (*p* = 0.06)<br>• Cardiac measures for IVST in diastole and EF showed no differences between the groups
Lin 2013 [3]	Agalsidase beta or agalsidase alfa, 36 (including 7 who switched to agalsidase alfa – data not separated)	Median: 16 months	• Patients from Taiwan, including 15 with the IVS4 + 919G > A mutation<br>• At follow-up<br>○ LVPWT, mean (SD): 10.3 (2.4) mm; *p* = 0.024 vs baseline<br>○ LVSWT, mean (SD): 12.6 (4.2) mm; *p* = 0.006 vs baseline
Lin 2014 [4]	Agalsidase beta or agalsidase alfa, 9	Agalsidase beta: range, 0.7, 88.6 months<br><br>Agalsidase alfa: > 12 months	• Population from Taiwan<br>• Agalsidase beta treatment then switched to agalsidase alfa<br>• Range<br>○ LVPWT after ERT: 8.9, 12.2 mm<br>○ LVPWT change (%): −35, 33; *p* = 0.228<br>• Range<br>○ IVST after ERT: 8.9, 17.7 mm<br>○ IVST change (%): −27, 0; *p* = 0.348
Mignani 2008 [100]	Agalsidase beta, 30<br><br>Agalsidase alfa, 3	Mean (SD):<br>• Dialysis patients: 45.1 (19.8) months<br>• Transplant patients: 48.4 (13.2) months	• Patients from Italy receiving renal replacement therapy: kidney dialysis or renal transplant (ERT and kidney dialysis, *n* = 17; ERT and kidney transplant, *n* =17)<br>• LVPWT<br>○ Dialysis patients<br>– Mean LVPWT (cm) at year 1 (*n* = 15), 2 (*n* = 12) and 3 (*n* = 7), and change from baseline at year 3<br>▪ 1.41; 1.37; 1.46; 5%<br>○ Transplant patients<br>– Mean LVPWT (cm) at year 1 (*n* = 14), 2 (*n* = 13) and 3 (*n* = 12), and change from baseline at year 3<br>▪ 1.56; 1.51; 1.50; −4%<br>• IVSTd<br>○ Dialysis patients<br>– Mean IVSTd (cm) at year 1 (*n* = 15), 2 (*n* = 12) and 3 (*n* = 7), and change from baseline at year 3<br>▪ 1.56; 1.50; 1.68; 8%<br>○ Transplant patients<br>– Mean IVSTd (cm) at year 1 (*n* = 14), 2 (*n* = 13) and 3 (*n* = 12), and change from baseline at year 3

Author, year	*N*	Treatment duration	Key results
			▪ 1.65; 1.69; 1.49; −9%
Miwa 2019 [6], Miwa 2018* [7]	ERT, 30	Mean (SD): 7.2 (4.6) years	• Study in Japanese patients<br>• Mean (SD) annual change<br>o LVPWT, mm<br>– Follow-up cohort: 0.07 (0.32)<br>– Males: 1.07 (2.80)<br>– Females: 0.01 (0.39)<br>o IVST, mm<br>– Follow-up cohort: 0.01 (0.50)<br>– Males: 1.01 (2.20)<br>– Females: −0.24 (0.46)<br>o EF, %<br>– Follow-up cohort: −0.18 (3.32)<br>– Males: −2.40 (1.09)<br>– Females: 1.09 (1.03)
Schmied 2016 [84]	ERT, 38	Median (SD): 6.4 (1.2) years	• Patients with FD treated at a single centre in Zurich, Switzerland<br>• Mean (SD) LVMI, g/m^2<br>o Males at follow-up: 168 (53); $p < 0.005$ vs baseline<br>o Females at follow-up: 103 (64)<br>• Mean (SD) LVPWT, mm<br>o Males at follow-up: 1.1 (0.3)<br>o Females at follow-up: 1.1 (0.5)<br>• Mean (SD) LVSWT, mm<br>o Males at follow-up, septum: 1.5 (0.5); $p < 0.05$ vs baseline<br>o Females at follow-up, septum: 1.2 (0.6)<br>• Mean (SD) LVEF, %<br>o Males at follow-up: 66 (4)<br>o Females at follow-up: 69 (9)
Talbot 2015 [11]	ERT, 25<br><br>(CKD5: *n* = 10; non-CKD5: *n* = 15)	≤10 years	• Assessed the impact of ESRD (CKD5) on CV outcomes in male patients in Australia with FD on ERT<br>• Over the course of the study<br>o Mean (SD) PWT (mm)<br>– Non-CKD5<br>▪ Year 1: 10.2 (1.9)<br>▪ Year 2: 10.5 (2.6)<br>▪ Year 5: 10.8 (2.3)<br>▪ Year 7: 10.9 (2.4)<br>▪ Year 10: 10.4 (2.6)<br>▪ $p = 0.83$ over time<br>– CKD5<br>▪ Year 1: 13.9 (3.6)<br>▪ Year 2: 13.0 (3.5)<br>▪ Year 5: 15.0 (2.5)<br>▪ Year 7: 14.7 (2.1)<br>▪ Year 10: 15.4 (3.9)<br>o Mean (SD) IVST (mm)<br>– Non-CKD5

Author, year	N	Treatment duration	Key results
			▪ Year 1: 10.3 (2.2)<br>▪ Year 2: 10.4 (2.7)<br>▪ Year 5: 10.8 (2.2)<br>▪ Year 7: 11.4 (2.1)<br>▪ year 10: 11.2 (2.1)<br>▪ *p* = 0.61 over time<br>– CKD5<br>▪ Year 1: 15.7 (4.1)<br>▪ Year 2: 15.6 (3.9)<br>▪ Year 5: 16.3 (5.0)<br>▪ Year 7: 18.2 (6.0)<br>▪ Year 10: 21.2 (7.3)<br>• Mean (SD) markers of diastolic function (Doppler-derived E/Ea, isovolumic relaxation time and deceleration time) were significantly greater (all *p* < 0.001) in the CKD5 subgroup vs the non-CKD5 subgroup
**Migalastat single arm studies**			
Camporeale 2023 [86]	16	18 months	• Baseline (*n* = 15): median [range] LVWT: 13.0 [10.0, 17.0] mm<br>• Follow-up: median [range] LVWT: 12.0 [10.0, 19.0] mm<br>• *p* = 0.19
Germain 2016 [16]	67	6 months plus 6- to 12-month open-label extension plus an additional year	• Assessments of the FACETS study<br>• Follow-up at month 18/24<br>○ Mean (SEM) [95% CI] LVPWT change from baseline end-diastolic LVPWT was stable for up to 24 months (*N* = 28: 0.014 (0.034) [−0.058, 0.087]) cm<br>○ Mean (SEM) [95% CI] end-diastolic IVST changed by −0.061 (0.051) cm [−1.67, 0.045], representing a decrease of 5.2%Changes in the LVMI correlated with changes in IVST (R^2 = 0.26; *p* = 0.006) but not with changes in LVPWT (R^2 = 0.06; *p* = 0.23)
Müntze 2019 [39]	21 (12 patients with MRI data completed the 1-year follow-up)	12 months	• Cardiac assessment of patients with amenable *GLA* mutations in the prospective monocentric HEAL-FABRY registry<br>• Distinctive changes over time were observed not only in diastolic but also systolic parameters. The systolic myocardial mass index was reduced by 2.39% (*p* = 0.10)

*Congress abstract.

ACEI, angiotensin-converting enzyme inhibitor. AHA, American Heart Association. ARB, angiotensin receptor blocker. CI, confidence interval. CKD, chronic kidney disease. CV, cardiovascular. ECG, electrocardiogram. EDV, end-diastolic volume. E/Ea, xxx. EF, ejection fraction. ERT, enzyme replacement therapy. ESRD, end-stage renal disease. ESV, end-systolic volume. FD, Fabry disease. FOS-MSSI, Fabry Outcome Survey-Mainz Severity Score Index. HRI, high renal involvement. IQR, interquartile range. ITT, intention-to-treat. IVST, interventricular septal thickness. IVSTd, interventricular septal thickness in diastole. LRI, low renal involvement. LS, least-squares. LV, left ventricular. LVEDD, left ventricular end-diastolic diameter. LVEF, left ventricular ejection fraction. LVH, left ventricular hypertrophy. LVM, left ventricular mass. LVMI, left ventricular mass index. LVPWT, left ventricular posterior wall thickness. LVPWTd, left ventricular posterior wall thickness in diastole. LVSWT, left ventricular septum wall thickness. MRI, magnetic resonance imaging. MWT, maximum wall thickness. NR, not reported. NS, not significant. PWT, posterior wall thickness. PWTd, posterior wall thickness in diastole. q2w, every 2 weeks. q4w, every 4 weeks. RV, right ventricular. RVEF, right ventricular ejection fraction. SD, standard deviation. SEM, standard error of the mean. SV, stroke volume. VT, ventricular tachycardia.

B

Author, year	Treatment/comparator arms, *n*	Treatment duration	Key results
**Comparator studies**			
Kramer 2014 [101]	ERT, 57<br>No ERT, 16	Mean (SD): 4.8 (2.4) years	• At follow-up<br>○ LVSWT, mean (SD)<br>– ERT (*n* = 56): 12.3 (3.7) mm<br>– No ERT (*n* = 16): 8.6 (1.9) mm<br>– $p < 0.05$ vs no ERT at follow-up<br>○ LVEF, mean (SD)<br>– ERT (*n* = 56): 63 (9) mm<br>– No ERT (*n* = 16): 59 (8) mm
Madsen 2017 [102]	ERT, 47<br>No ERT, 19	ERT: median [range] 8 [0, 12] years<br><br>No ERT: median [range] r 6 [0, 13] years	• Assessed progression of cardiac involvement in a Danish cohort<br>• Cardiac structure data at baseline, median [range]<br>○ IVSTd (mm)<br>– ERT (*n* = 46): 11 [5, 22]; no ERT (*n* = 17): 11 [5, 20]<br>○ LVPWT (mm)<br>– ERT (*n* = 45): 11 [6, 18]; no ERT (*n* = 17) 10 [6, 22]<br>○ LVIDd (mm)<br>– ERT (*n* = 44): 48 [37, 58]; no ERT (*n* = 17): 44 [33, 53]<br>○ LVEF (%)<br>– ERT (*n* = 43): 59 [55, 60]; no ERT (*n* = 18) 58 [45, 63]<br>• Cardiac structure data at follow-up, median [range]<br>○ IVSTd (mm)<br>– ERT (*n* = 40): 12[8, 23]; no ERT (*n* = 14): 8 [5, 12]<br>○ LVPWT (mm)<br>– ERT (*n* = 40): 12 [8, 22]; no ERT (*n* = 14): 8 [6, 10]<br>○ LVIDd (mm<br>– ERT (*n* = 39): 47 [36, 61]; no ERT (*n* = 14): 48 [40, 55]<br>○ LVEF (%)<br>– ERT (*n* = 38): 58 [50, 63]; no ERT (*n* = 14): 57 [47, 63]<br>• Cardiac structure data at follow-up in patients receiving ERT by presence of cardiac disease at baseline, median [range]<br>○ IVSTd (mm)<br>– Cardiac disease (*n* = 31): 12 [8, 23]; no cardiac disease (*n* = 9): 8 [5, 12]<br>○ LVPWT (mm)<br>– Cardiac disease (*n* = 31): 12 [8, 22]; no cardiac disease (*n* = 9): 8 [6, 10]<br>○ LVIDd (mm)<br>– Cardiac disease (*n* = 31): 47 [36, 61]; no cardiac disease (*n* = 8): 48 [40, 55]<br>○ LVEF (%)<br>– Cardiac disease (*n* = 30): 58 [50, 63]; no cardiac disease (*n* = 8): 60 [57, 60]
Niemann 2010 [103]	ERT, 57<br>No ERT, 18	Mean (SD) 3.1 (1.8) years	• Assessment of the effects of ERT on right ventricular morphology and function<br>○ Baseline / follow-up mean (SD) RV end-diastolic diameter (mm)<br>– ERT: 29.3 (6.6) / 29.9 (4.4)<br>– No ERT: 27.6 (4.7) / 27.2 (3.2)

Author, year	Treatment/comparator arms, *n*	Treatment duration	Key results
			○ Baseline / follow-up mean (SD) RV wall thickness (mm)<br>– ERT: 6.9 (1.6) / 6.7 (1.5)<br>– No ERT: 5.2 (1.1) / 5.2 (1.3)<br>– *p* < 0.05 ERT vs no ERT group at baseline
Nordin 2019 [83]	Pre-ERT group, 20<br><br>Established ERT, 18<br><br>No ERT, 18	Follow-up, mean (SD): 1.1 (0.2) years<br><br>Median [range]<br>• Pre-ERT group: 1 year<br>• Established ERT: 4.2 [1.4, 12.2] years<br>• No ERT: 1 year	• MWT<br>○ Pre-ERT (initiated ERT)<br>– Small reduction in MWT: 14.8 (5.9) mm at baseline to 14.4 (5.7) mm; *p* = 0.028<br>○ Established ERT (advanced stable disease)<br>– No change in MWT: 17.5 (4.7) mm at baseline to 17.8 (4.9) mm; *p* = 0.056<br>○ No ERT (early disease, mostly females)<br>– There was a small increase in MWT: 9.8 (2.7) mm at baseline to 10.2 (2.6) mm; *p* = 0.010
Pogoda 2023 [104]	Untreated controls: 30<br>Migalastat-treated: 20<br>ERT-treated: 48	≥ 81 months	• A prospective observational single-center study in Germany<br>• The control group was untreated females with FD who were younger than the treated group<br>• Median [range] IVS at baseline, mm<br>○ Untreated females: 10 [7, 18]<br>○ Migalastat-treated females: 12.5 [9.0, 17.0]<br>○ ERT-treated females: 14.0 [7.0, 22.0]<br>○ Migalastat-treated males: 15.0 [11.0, 27.0]<br>○ ERT-treated males: 14.5 [10.0, 30.0]<br>• Change per year in IVS from baseline, median [range] mm<br>○ Untreated females: 0.0 [−3.0, 3.0]<br>○ Migalastat-treated females: −0.29 [−1.2, 1.6]<br>○ ERT-treated females: 0.0 [−0.6, 2.1]<br>○ Migalastat-treated males: 0.00 [−5.1, 1.0]<br>○ ERT-treated males: 0.11 [−1.7, 3.5]<br>• Median [range] LVEF at baseline, %<br>○ Untreated females: 61.2 [41.3, 81.0]<br>○ Migalastat-treated females: 63.0 [51.6, 74.6)<br>○ ERT-treated females: 60.0 [50.0, 67.0]<br>○ Migalastat-treated males: 54.4 [33.9, 75.6]<br>○ ERT-treated males: 62.8 [33.7, 71.0]<br>• Change per year in LVEF from baseline, median [range] %<br>○ Untreated females: 0.7 [−14.9, 9.5]<br>○ Migalastat-treated females: −0.29 [−8.5, 15.3]<br>○ ERT-treated females: 0.1 [−3.5, 26.8]<br>○ Migalastat-treated males: 0.9 [−18.4, 8.9]<br>○ ERT-treated males: 0.77 [−7.6, 6.2]<br>• Independent of treatment, yearly changes in cardiac parameters in females and males were stable, pointing at disease stabilization

Author, year	Treatment/comparator arms, *n*	Treatment duration	Key results
Weidemann 2003 [105]	Agalsidase beta, 16<br>Age-matched healthy controls, 16	12 months	• The FD patients and the healthy control group showed a normal EF at baseline and there was no change during ERT<br>• Mean (SD) LVPWT at baseline was significantly higher in FD patients compared with controls: 13.8 (0.6) mm vs 7.2 (0.3) mm, $p < 0.001$<br>• At 12 months, mean (SEM)<br>o Myocardial mass (g) in patients with FD at 12 months (n = 10): 180 (21); $p < 0.05$ vs baseline<br>o End-diastolic thickness of the LV posterior wall (mm) of patients after 12 months: 11.8 (0.6); $p < 0.05$ vs baseline
Weidemann 2009 [85]	Agalsidase beta, 32<br>Control, 20	3 years	• The benefit of ERT was assessed in patients at an early stage of the disease showing no or little myocardial fibrosis<br>• LVPWT and IVSTd were significantly higher in patients compared with healthy control subjects (LVPWT: 13.9 (2.1) mm vs 8.6 (1.4) mm; IVSTd: 14,3 (2.1) mm vs 8.9 (1.0) mm; $p < 0.001$<br>• Subgroup analysis included patients with no fibrosis (n = 12): mild fibrosis (n = 11): or severe fibrosis (n = 9):<br>• At follow-up<br>o PWT (mm) during ERT by fibrosis status, mean (SD)<br>– No fibrosis: baseline, 13.0 (1.2); 1 year, 11.7 (1.6); 2 years, 11.3 (1.8); 3 years, 11.5 (1.6); p = 0.01<br>– Mild fibrosis: baseline, 14.4 (2.2); 1 year, 13.1 (2.4); 2 years, 13.3 (2.4); 3 years, 12.9 (1.8); p = 0.21<br>– Severe fibrosis: baseline, 14.7 (2.7); 1 year, 13.3 (2.3); 2 years, 13.4 (2.5); 3 years, 12.8 (2.3); p = 0.17<br>o SWT (mm) during ERT by fibrosis status, mean (SD)<br>– No fibrosis: baseline, 13.5 (1.4); 1 year, 12.1 (1.0); 2 years, 11.9 (1.2); 3 years, 12.0 (1.4); p = 0.01<br>– Mild fibrosis: baseline, 14.4 (1.8); 1 year, 13.3 (1.9); 2 years, 13.4 (1.9); 3 years, 13.4 (1.4); p = 0.20<br>– Severe fibrosis: baseline, 14.9 (3.0); 1 year, 14.3 (2.7); 2 years, 14.3 (2.6); 3 years, 14.1 (2.0); p = 0.01<br>o LVEDD (mm) during ERT by fibrosis status, mean (SD)<br>– No fibrosis: baseline, 48 (4); 1 year , 49 (4); 2 years, 49 (6); 3 years, 49 (4); p = 0.89<br>– Mild fibrosis: baseline, 49 (5); 1 year, 48 (5); 2 years, 47 (5); 3 years, 48 (6); p = 0.99<br>– Severe fibrosis: baseline, 51 (12); 1 year, 49 (5); 2 years, 47 (3); 3 years, 49 (6); p = 0.69

CI, confidence interval. EF, ejection fraction. ERT, enzyme replacement therapy. FD, Fabry disease. IVSTd, interventricular septal thickness in diastole. LV, left ventricular. LVEDD, left ventricular end-diastolic diameter. LVEF, left ventricular ejection fraction. LVIDd, left ventricular internal diameter in diastole. LVPWT, left ventricular posterior wall thickness. LVSWT, left ventricular septum wall thickness. MWT, maximum wall thickness. PWT, posterior wall thickness. RV, right ventricular. SD, standard deviation. SEM, standard error of the mean. SWT, septal wall thickness.

C

Author, year	Treatment groups, *n*	Post-switch treatment duration	Key results
**Switch studies**			
Hughes 2017 [34]	Migalastat, 36<br>Ongoing ERT, 24	18 months	• Change from baseline in mean (95% CI) LVPWT, cm<br>o Migalastat (n = 33): −0.035 (−0.077, 0.007)<br>o ERT (n = 16): 0.029 (−0.037, 0.094)<br>• Change from baseline in mean (95% CI) LVSWT, cm<br>o Migalastat (n = 33): 0.058 (−0.200, 0.140)<br>o ERT (n = 16): 0.037 (−0.051, 0.124)
Kramer 2018 [36]	Agalsidase beta, 37<br>Switch group (agalsidase alfa), 38	Long-term follow-up 1 (LTFU1); ≥ 12 months	• Switch/ re-switching study<br>• Mean (SD) LVSd, mm, LTFU1; LTFU2<br>o Regular dose: 12.7 (3.3); 12.8 (3.8)

Author, year	Treatment groups, *n*	Post-switch treatment duration	Key results
	Re-switch group (agalsidase beta), 37	Long-term follow-up 2 (LTFU2); ≥ 24 months	○ Switch group: 12.8 (3.1); 13.4 (3.4)<br>○ Re-switch: 12.8 (3.8); 13.5 (3.4)<br>• Mean (SD) LVEF, %, LTFU1; LTFU2<br>○ Regular dose: 57 (15); 58 (10)<br>○ Switch group: 61 (7); 62 (8)<br>○ Re-switch: 58 (7); 60 (9)
Riccio 2020 [41]	ERT switch to migalastat, 7	12 months	• Mean (SD) LVPWT in male patients, mm<br>○ Baseline: 9.00 (2.51)<br>○ 12 months ERT: 9.28 (2.21)<br>○ 12 months migalastat: 8.85 (2.34)<br>• Mean (SD) IVST, mm<br>○ Baseline: 10.14 (2.47)<br>○ 12 months ERT: 10.00 (2.23)<br>○ 12 months migalastat: 9.71 (2.05)<br>• Mean (SD) LVEF, %<br>○ Baseline: 59.71 (4.38)<br>○ 12 months ERT: 60.42 (4.68)<br>○ A12 months migalastat: 58.42 (5.74)

CI, confidence interval. ERT, enzyme replacement therapy. IVST, interventricular septal thickness. LVEF, left ventricular ejection fraction. LVPWT, left ventricular posterior wall thickness. LVSd, left ventricular septum thickness in diastole. LVSWT, left ventricular septum wall thickness.

Supplementary Table S8

Overview of cerebrovascular data from (A) mixed or non-specified ERT single-arm studies and single-arm studies for other treatments and (B) switch studies.

A

Author, year	*N*	Treatment duration	Key results
**Mixed or non-specified ERT single-arm studies**			
Arends 2017a [1]	Agalsidase alfa or beta (switching allowed), 293	Median: 6.8 years	• Multicentre, retrospective cohort, observational study<br>• Before ERT, cerebral events (stroke or TIA) occurred in 41 (14%) patients, and WMLs were present in 75 (61%) evaluated patients. During follow-up, 30 (10.2%) patients developed a stroke or TIA<br>• In patients who had a cerebral event before ERT initiation, the risk of developing another cerebral event was tripled<br>o However, adjusted for age, sex and phenotype, these events were not significantly associated with clinical event rate (HR: 1.54; 95% CI: 0.86, 2.77)<br>o WMLs were also not associated with disease progression (HR: 1.02; 95% CI: 0.40, 2.57)
Arends 2017b [106]	Agalsidase alfa or beta (switching allowed), 85<br><br>Treatment start<br>• Early (< 25 years): 21<br>• Late (≥ 25 years): 64	Median: 6.7 years (early treatment group), 5.4 years (late-treatment group)	• Multicentre, retrospective cohort, observational study in men with classic FD who started ERT before the age of 25 compared with those who started later in life<br>• At baseline, WMLs were present in five (31%) patients who initiated treatment early and 15 (83%) who initiated treatment later<br>• One (5%) patient in the early treatment group had an ischaemic stroke 12 years after initiation of ERT at age 30 years (7 events per 1000 patient-years)<br>• In the late treatment group, 36 (57%) patients developed one or more clinical events (114 events per 1000 patient-years)<br>o Of these, 11 (31%) patients experienced cerebral events
Korver 2020 [107]	ERT, 88	Median [range]: 7.9 [0.1, 15.8] years	• Retrospective cohort study describing WML, basilar artery diameter and infarction progression<br>• Cerebral infarctions on MRI were present in 23 (15.6%) patients at baseline and in 42 (28.2%) patients at the end of follow-up<br>• Median (range) basilar artery diameter was 3.33 (1.85, 5.83) mm at baseline and increased to 3.67 (1.85, 7.25) mm at follow-up<br>• Both WML severity and basilar artery diameter progressed with age, with differences in rate of progression between the sex and phenotype<br>• Additional effects of established cardiovascular risk factors, organ involvement and treatment with ERT (for ≥ 6 months vs < 6 months or 'early' treatment start [< 30 years]) are probably small to negligible<br>• Infarction rate was highest in men with a classic phenotype, with a median infarction-free survival of 46.5 years
Lulla 2020* [108]	ERT, 31	66.6% of patients reported duration on ERT of > 5 years	• Survey evaluating the prevalence and severity of neuropsychiatric symptoms among patients with FD<br>• 32.3% of patients reported a stroke or TIA
Miwa 2019 [6], Miwa 2018* [7]	ERT, 30	Mean (SD): 7.2 (4.6) years	• Study in Japanese patients<br>• Mean (SD) annual change in basilar artery diameter, mm<br>o Follow-up cohort: 0.07 (0.14)<br>o Males: 0.12 (0.18)<br>o Females: 0.02 (0.06)

Author, year	N	Treatment duration	Key results
			• The annual basal artery diameter change correlated inversely with the duration of ERT in males(beta = −0.72; *p* = 0.019)
Nowak 2017 [109]	ERT, 67	Median [IQR]: 9 [6, 12] years	• Retrospective analysis of a prospective multicentre cohort<br>• Among 21 treated female patients, one (5%) experienced a new-onset aerial fibrillation, five (24%) experienced strokes and one (5%) needed a pacemaker<br>o No events occurred among the 22 untreated female patients.<br>• Among 24 treated male patients, four (17%) experienced a new-onset aerial fibrillation, four (17%) experienced strokes, one (4%) myocardial infarction and two (8%) needed a pacemaker
Ramaswami 2019 [64]	31	5 years	• Male paediatric patients<br>• Basilar artery diameter, mean, median (SD) [range], mm<br>• Year 5 overall (*n* = 18): 2.8, 2.8 (0.7) [1.7, 1.2]
Rombach 2013 [8]	Agalsidase alfa or beta, 75<br><br>Patients in prospective follow-up<br>• Adults, 57<br>• Adolescents, 6	Median [range]: 5.2 [0.1, 11.0] years	• Males<br>o Males with follow-up MRIs (*n* = 25): 12 (48%) developed new WMLs<br>o Median [range] duration of ERT at the time of new WML: 3.1 [0.9, 8.1] years<br>o Time to development of WMLs was not associated with baseline WML, *p* = 0.44<br>• Females<br>o Of female patients with follow-up MRIs (n = 25): 7 (28%) developed new WMLs<br>o Median [range] duration of ERT at the time of new WML 4.0 [1.0, 6.1] years<br>o Time to development of WMLs was not associated with baseline WML, *p* = 0.77<br>• Of adolescents (*n* = 6), one female patient and one male patient developed WMLs; another female patient with previous WML developed an asymptomatic lacunar infarction
Wyatt 2012 [12], Anderson 2014 [13]	Agalsidase alfa or beta (switching allowed), 311	Median (range)<br>• Adult males: 3.51 (0, 9.72) years<br>• Adult females: 3.55 (0, 8.77) years<br>• Male children: 1.2 (0, 4.2) years<br>• Female children: 2.4 (1.7, 3.0) years	• Longitudinal, retrospective prospective cohort study<br>• At recruitment, 20 patients (13 males) had a TIA or stroke<br>o Median [range] duration of ERT at the time of stroke: 3.75 [0, 8.43] years<br>o No further strokes were reported during the study period<br>• Patients on or off ERT had a low probability of having a TIA or stroke before 40 years of age, but the risk of having a TIA or stroke increased thereafter<br>o There was no statistically significant association between treatment status and the probability of having a TIA or stroke; HR = 2.09 (95% CI 0.68, 6.44), *p* = 0.19
**Migalastat single-arm studies**			
Sunder-Plassmann 2019* [110]	Migalastat, 114	Median [range]: 4.4 [0.1, 8.3] years	• Meta-analysis of four phase 2 and four phase 3 clinical trials<br>• During treatment, 11 cerebrovascular events were reported for eight (7%) patients (6/8 patients had a history of cerebrovascular events)<br>• The mean (SD) age of patients at first event during migalastat treatment was 50.4 (15.5) years
Hopkin 2022* [19]	Migalastat, 97	Median [IQR]: 5.1 [2.3, 6.8] years	• Observational study of data from three phase 3 trials and their open-label extensions<br>• Incidence of cerebrovascular events was 13.2 per 1000 patient-years

C CI, confidence interval. CV, cardiovascular. ERT, enzyme replacement therapy. FD, Fabry disease. HR, hazard ratio. MRI, magnetic resonance imaging. NR, not reported. OR, odds ratio. TIA, transient ischemic attack. WML, white matter lesion.

B

**Author, year**	***N***	**Treatment duration**	**Key results**
Switch studies			
Kramer 2018 [36]	Agalsidase beta, 37<br>Switch group (agalsidase alfa), 38<br>Re-switch group (agalsidase beta), 37	Long-term follow-up 1; ≥ 12 months<br>Long-term follow-up 2; ≥ 24 months	• Switch/re-switch study<br>• The regular dose and the switch groups showed decreasing frequencies of stroke/TIA between baseline and the second follow-up (both $p < 0.05$)<br>• The re-switch group showed a significant increase in the frequencies of stroke/TIA during the first follow-up ($p < 0.05$) and a subsequent decrease at the second follow-up ($p < 0.05$) with a comparable frequency to baseline<br>• Two female patients (aged 66 and 74 years in the switch and re-switch groups, respectively) died owing to stroke

CI, confidence interval. TIA, transient

Supplementary Figure S1. Study locations

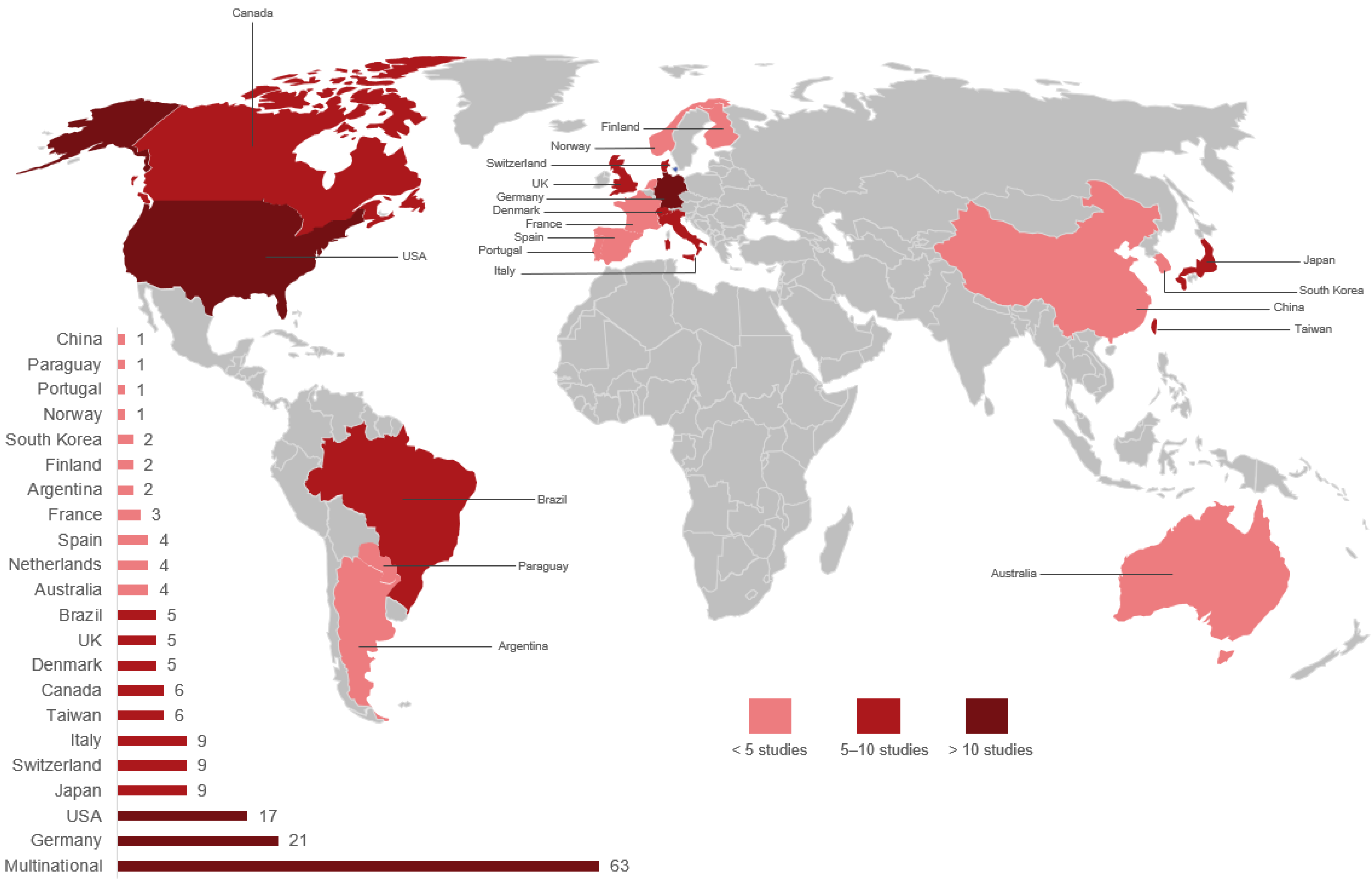

Supplementary references

[1] M. Arends, M. Biegstraaten, D.A. Hughes, A. Mehta, P.M. Elliott, D. Oder, et al., Retrospective study of long-term outcomes of enzyme replacement therapy in Fabry disease: Analysis of prognostic factors, PLoS ONE 12(8) (no pagination) (2017).

[2] M. Goicoechea, F. Gomez-Preciado, S. Benito, J. Jorras, R. Torra, A. Huerta, et al., Predictors of outcome in a Spanish cohort of patients with Fabry disease on enzyme replacement therapy, Nefrologia : publicacion oficial de la Sociedad Espanola Nefrologia. 10 (2021).

[3] H.Y. Lin, H.C. Liu, Y.H. Huang, H.C. Liao, T.R. Hsu, C.I. Shen, et al., Effects of enzyme replacement therapy i for cardiac-type Fabry patients with a Chinese hotspot late-onset Fabry mutation (IVS4+919G>A), BMJ Open 3(7) (no pagination) (2013).

[4] H.Y. Lin, Y.H. Huang, H.C. Liao, H.C. Liu, T.R. Hsu, C.I. Shen, et al., Clinical observations on enzyme replacement therapy in patients with Fabry disease and the switch from agalsidase beta to agalsidase alfa, J Chin Med Assoc 77 (2014) 190-197.

[5] C.V. Madsen, H. Granqvist, H. Petersen Jo, A.K. Rasmussen, A.M. Lund, P. Oturai, et al., Age-related renal function decline in Fabry disease patients on enzyme replacement therapy: A longitudinal cohort study, Nephrology Dialysis Transplantation 34(9) (2019) 1525-1533.

[6] K. Miwa, Y. Yagita, M. Sakaguchi, K. Kitagawa, N. Sakai, H. Mochizuki, Effect of enzyme replacement therapy on basilar artery diameter in male patients with Fabry disease, Stroke 50(4) (2019) 1010-1012.

[7] K. Miwa, N. Sakai, Y. Yagita, M. Takeuchi, K. Todo, M. Sakaguchi, et al., Basilar artery diameter is associated with cerebral small vessel disease and the duration of enzyme-replacement therapy in Fabry disease, Stroke. Conference: American Heart Association/American Stroke Association 49 (2018).

[8] S.M. Rombach, B.E. Smid, M.G. Bouwman, G.E. Linthorst, M.G. Dijkgraaf, C.E. Hollak, Long term enzyme replacement therapy for Fabry disease: effectiveness on kidney, heart and brain, Orphanet Journal Of Rare Diseases 8 (2013) 47.

[9] S.M. Sirrs, D.G. Bichet, R. Casey, J.T. Clarke, K. Lemoine, S. Doucette, et al., Outcomes of patients treated through the Canadian Fabry disease initiative, Mol Genet Metab 111 (2014) 499-506.

[10] R. Skrunes, C. Tondel, S. Leh, K.K. Larsen, G. Houge, E.S. Davidsen, et al., Long-term dose-dependent agalsidase effects on kidney histology in Fabry disease, Clinical Journal of the American Society of Nephrology 12(9) (2017) 1470-1479.

[11] A.S. Talbot, N.T. Lewis, K.M. Nicholls, Cardiovascular outcomes in Fabry disease are linked to severity of chronic kidney disease, Heart 101 (2015) 287-293.

[12] K. Wyatt, W. Henley, L. Anderson, R. Anderson, V. Nikolaou, K. Stein, et al., The effectiveness and cost-effectiveness of enzyme and substrate replacement therapies: A longitudinal cohort study of people with lysosomal storage disorders, Health Technology Assessment 16(39) (2012) 1-566.

[13] L.J. Anderson, K.M. Wyatt, W. Henley, V. Nikolaou, S. Waldek, D.A. Hughes, et al., Long-term effectiveness of enzyme replacement therapy in Fabry disease: results from the NCS-LSD cohort study, Journal of Inherited Metabolic Disease 37(6) (2014) 969-978.

[14] D.G. Bichet, R. Torra, E. Wallace, D. Hughes, R. Giugliani, N. Skuban, et al., Long-term follow-up of renal function in patients treated with migalastat for Fabry disease, Mol Genet Metab Rep 28 (2021) 100786.

[15] D. Hughes, G. Sunder-Plassmann, A. Jovanovic, E. Brand, M.L. West, D.G. Bichet, et al., FollowME Fabry Pathfinders registry: Renal effectiveness in a multi-national, multi-

center cohort of patients on migalastat treatment for at least three years, Molecular Genetics and Metabolism. Conference: WORLDSymposiumTM 138 (2023).
[16] D.P. Germain, D.A. Hughes, K. Nicholls, D.G. Bichet, R. Giugliani, W.R. Wilcox, et al., Treatment of Fabry's disease with the pharmacologic chaperone migalastat, New England Journal of Medicine 375(6) (2016) 545-555.
[17] D.P. Germain, K. Nicholls, R. Giugliani, D.G. Bichet, D.A. Hughes, L.M. Barisoni, et al., Efficacy of the pharmacologic chaperone migalastat in a subset of male patients with the classic phenotype of Fabry disease and migalastat-amenable variants: data from the phase 3 randomized, multicenter, double-blind clinical trial and extension study, Genetics in Medicine 21(9) (2019) 1987-1997.
[18] R. Giugliani, S. Waldek, D.P. Germain, K. Nicholls, D.G. Bichet, J.K. Simosky, et al., A Phase 2 study of migalastat hydrochloride in females with Fabry disease: selection of population, safety and pharmacodynamic effects, Mol Genet Metab 109 (2013) 86-92.
[19] R.J. Hopkin, D.G. Bichet, G. Sunder-Plassmann, K. Nicholls, I. Olivotto, R. Giugliani, et al., Long-term multisystemic efficacy with migalastat in ERT-naive and ERT-experienced patients with amenable GLA variants, Molecular Genetics and Metabolism 135(2) (2022) S56.
[20] M. Lenders, P. Nordbeck, C. Kurschat, N. Karabul, J. Kaufeld, J.B. Hennermann, et al., Treatment of Fabry's disease with migalastat: Outcome from a prospective observational multicenter study (FAMOUS), Clinical Pharmacology and Therapeutics 108(2) (2020) 326-337.
[21] M. Lenders, P. Nordbeck, C. Kurschat, M. Eveslage, N. Karabul, J. Kaufeld, et al., Treatment of Fabry disease with migalastat-outcome from a prospective 24 months observational multicenter study (FAMOUS), European heart journal. Cardiovascular pharmacotherapy. 16 (2021).
[22] C. Orsborne, L. Thompson, A. Jovanovic, A retrospective outcome analysis of chaperone therapy in Fabry disease: Clinical outcomes after the first year of therapy - A single centre experience, Molecular Genetics and Metabolism 129(2) (2020) S123.
[23] U. Ramaswami, W. Wilcox, R.J. Hopkin, H. Yang, H. Jiang, V. Lengoc, Migalastat HCl 150mg every other day is well-tolerated and efficacious in adolescent patients with Fabry disease, Molecular Genetics and Metabolism 135(2) (2022) S104.
[24] N. Skuban, U. Feldt-Rasmussen, R. Giugliani, D.P. Germain, D.A. Hughes, W.R. Wilcox, et al., Efficacy and safety of migalastat, an oral pharmacologic chaperone for Fabry disease: Results from two randomized phase 3 studies, FACETS and ATTRACT, Nephron 136(3) (2017) 175.
[25] R. Torra, D. Germain, D. Bichet, R. Schiffmann, J. Yu, J. Castelli, et al., Clinical outcomes with migalastat in patients with Fabry disease based on degree of renal impairment: Results from phase 3 trials, Nephrology Dialysis Transplantation 33(Suppl 1) (2018) i346–i356.
[26] M.L. West, D. Hughes, G. Sunder-Plassmann, A. Jovanovic, E. Brand, D.G. Bichet, et al., FollowME Fabry Pathfinders Registry: Renal Effectiveness in a Cohort of Patients on Migalastat Treatment for at Least Three Years, Journal of the American Society of Nephrology. Conference: Kidney Week 34 (2023).
[27] J. Bernat, M. Holida, N. Longo, O. Goker-Alpan, E. Wallace, P. Deegan, et al., eP149: Safety and efficacy of pegunigalsidase alfa, every 4 weeks, in Fabry disease: Results from the phase 3, open-label, BRIGHT study, Genetics in Medicine 24(3 Supplement) (2022) S91-S92.
[28] J. Bernat, M.D. Holida, N. Longo, O. Goker-Alpan, E. Wallace, P.B. Deegan, et al., Long-term safety and efficacy of pegunigalsidase alfa administered every 4 weeks in patients with Fabry disease: Two-year interim results from the ongoing phase 3

BRIGHT51 open-label extension study, Molecular Genetics and Metabolism. Conference: WORLDSymposiumTM 138 (2023).
[29] D. Hughes, D. Gonzalez, G. Maegawa, J.A. Bernat, M. Holida, P. Giraldo, et al., Long-term safety and efficacy of pegunigalsidase alfa: A multicenter 6-year study in adult patients with Fabry disease, Genetics in Medicine 25 (2023) 100968.
[30] R. Schiffmann, O. Goker-Alpan, M. Holida, P. Giraldo, L. Barisoni, R.B. Colvin, et al., Pegunigalsidase alfa, a novel PEGylated enzyme replacement therapy for Fabry disease, provides sustained plasma concentrations and favorable pharmacodynamics: A 1-year Phase 1/2 clinical trial, Journal of Inherited Metabolic Disease 42(3) (2019) 534-544.
[31] A. Khan, D.L. Barber, J. Huang, C.A. Rupar, J.W. Rip, C. Auray-Blais, et al., Lentivirus-mediated gene therapy for Fabry disease, Nat Commun 12 (2021) 1178.
[32] O. Goker-Alpan, K. Nedd, S.P. Shankar, Y.H. Lien, N. Weinreb, A. Wijatyk, et al., Effect and tolerability of agalsidase alfa in patients with Fabry disease who were treatment naive or formerly treated with agalsidase beta or agalsidase alfa, JIMD rep 23 (2015) 7-15.
[33] A. Linhart, G. Dostálová, K. Nicholls, M.L. West, C. Tøndel, A. Jovanovic, et al., Safety and efficacy of pegunigalsidase alfa in patients with Fabry disease who were previously treated with agalsidase alfa: results from BRIDGE, a phase 3 open-label study, Orphanet J Rare Dis 18 (2023) 332.
[34] D.A. Hughes, K. Nicholls, S.P. Shankar, G. Sunder-Plassmann, D. Koeller, K. Nedd, et al., Oral pharmacological chaperone migalastat compared with enzyme replacement therapy in Fabry disease: 18-month results from the randomised phase III ATTRACT study, Journal of Medical Genetics 54(4) (2017) 288-296.
[35] I. Narita, T. Ohashi, N. Sakai, T. Hamazaki, N. Skuban, J.P. Castelli, et al., Efficacy and safety of migalastat in a Japanese population: a subgroup analysis of the ATTRACT study, Clinical and Experimental Nephrology 24(2) (2020) 157-166.
[36] J. Kramer, M. Lenders, S. Canaan-Kuhl, P. Nordbeck, N. Uceyler, D. Blaschke, et al., Fabry disease under enzyme replacement therapy-new insights in efficacy of different dosages, Nephrology Dialysis Transplantation 33(8) (2018) 1362-1372.
[37] M. Lenders, S. Canaan-Kuhl, J. Kramer, T. Duning, S. Reiermann, C. Sommer, et al., Patients with Fabry disease after enzyme replacement therapy dose reduction and switch-2-year follow-up, Journal of the American Society of Nephrology 27(3) (2016) 952-962.
[38] M. Lenders, P. Nordbeck, S. Canaan-Kuhl, L. Kreul, T. Duning, L. Lorenz, et al., Treatment switch in Fabry disease- a matter of dose?, Journal of Medical Genetics 58(5) (2021) 342-350.
[39] J. Muntze, D. Gensler, O. Maniuc, D. Liu, T. Cairns, D. Oder, et al., Oral chaperone therapy migalastat for treating Fabry disease: Enzymatic response and serum biomarker changes after 1 year, Clinical Pharmacology and Therapeutics 105(5) (2019) 1224-1233.
[40] J. Muentze, D. Gensler, T. Salinger, D. Oder, C. Wanner, S. Frantz, et al., Treatment of cardiac manifestations in Fabry disease with the oral drug Migalastat: First 12 months results from a cohort of amenable all-comers, European Heart Journal 39(Supplement 1) (2018) 460.
[41] E. Riccio, M. Zanfardino, L. Ferreri, C. Santoro, S. Cocozza, I. Capuano, et al., Switch from enzyme replacement therapy to oral chaperone migalastat for treating Fabry disease: real-life data, Eur J Hum Genet 28 (2020) 1662-1668.
[42] K. Tsuboi, H. Yamamoto, Clinical observation of patients with Fabry disease after switching from agalsidase beta (Fabrazyme) to agalsidase alfa (Replagal), Genetics in Medicine 14(9) (2012) 779-786.

[43] K. Tsuboi, H. Yamamoto, Clinical course of patients with Fabry disease who were switched from agalsidase-beta to agalsidase-alpha, Genetics in Medicine 16(10) (2014) 766-772.
[44] F. Weidemann, J. Kramer, T. Duning, M. Lenders, S. Canaan-Kuhl, A. Krebs, et al., Patients with Fabry disease after enzyme replacement therapy dose reduction versus treatment switch, Journal of the American Society of Nephrology 25(4) (2014) 837-849.
[45] M. Cybulla, K. Nicholls, S. Feriozzi, A. Linhart, J. Torras, B. Vujkovac, et al., Renoprotective effect of agalsidase alfa: A long-term follow-up of patients with Fabry disease, J 11 (2022) 17.
[46] S. Feriozzi, J. Torras, M. Cybulla, K. Nicholls, G. Sunder-Plassmann, M. West, et al., The effectiveness of long-term agalsidase alfa therapy in the treatment of Fabry nephropathy, Clin J Am Soc Nephrol 7 (2012) 60-69.
[47] O. Goker-Alpan, N. Longo, M. McDonald, S.P. Shankar, R. Schiffmann, P. Chang, et al., An open-label clinical trial of agalsidase alfa enzyme replacement therapy in children with Fabry disease who are naive to enzyme replacement therapy, Drug Design, Development and Therapy 10 (2016) 1771-1781.
[48] D.A. Hughes, M.A. Barba Romero, C.E.M. Hollak, R. Giugliani, P.B. Deegan, Response of women with Fabry disease to enzyme replacement therapy: Comparison with men, using data from FOS-the Fabry Outcome Survey, Molecular Genetics and Metabolism 103(3) (2011) 207-214.
[49] L.B. Jardim, F. Aesse, L.M. Vedolin, C. Pitta-Pinheiro, J. Marconato, M.G. Burin, et al., White matter lesions in Fabry disease before and after enzyme replacement therapy: a 2-year follow-up, Arq Neuropsiquiatr 64 (2006) 711-717.
[50] C. Kampmann, A. Perrin, M. Beck, Effectiveness of agalsidase alfa enzyme replacement in Fabry disease: cardiac outcomes after 10 years' treatment, Orphanet Journal of Rare Diseases 10(1) (no pagination) (2015).
[51] M. Ries, J.T. Clarke, C. Whybra, M. Timmons, C. Robinson, B.L. Schlaggar, et al., Enzyme-replacement therapy with agalsidase alfa in children with Fabry disease, Pediatrics 118 (2006) 924-932.
[52] H. Sasa, M. Nagao, K. Kino, Safety and effectiveness of enzyme replacement therapy with agalsidase alfa in patients with Fabry disease: Post-marketing surveillance in Japan, Mol Genet Metab 126 (2019) 448-459.
[53] R. Schiffmann, M. Ries, M. Timmons, J.T. Flaherty, R.O. Brady, Long-term therapy with agalsidase alfa for Fabry disease: Safety and effects on renal function in a home infusion setting, Nephrology Dialysis Transplantation 21(2) (2006) 345-354.
[54] R. Schiffmann, H. Askari, M. Timmons, C. Robinson, W. Benko, R.O. Brady, et al., Weekly enzyme replacement therapy may slow decline of renal function in patients with Fabry disease who are on long-term biweekly dosing, Journal of the American Society of Nephrology 18(5) (2007) 1576-1583.
[55] R. Schiffmann, M. Ries, D. Blankenship, K. Nicholls, A. Mehta, J.T.R. Clarke, et al., Changes in plasma and urine globotriaosylceramide levels do not predict Fabry disease progression over 1 year of agalsidase alfa, Genetics in Medicine 15(12) (2013) 983-989.
[56] R. Schiffmann, G.M. Pastores, Y.H. Lien, V. Castaneda, P. Chang, R. Martin, et al., Agalsidase alfa in pediatric patients with Fabry disease: a 6.5-year open-label follow-up study, Orphanet Journal Of Rare Diseases 9 (2014) 169.
[57] A. Schwarting, F. Dehout, M. Beck, A. Mehta, G. Sunder-Plassmann, O. Bodamer, et al., Enzyme replacement therapy and renal function in 201 patients with Fabry disease, Clinical Nephrology 66(2) (2006) 77-84.
[58] S. Thofehrn, C. Netto, C. Cecchin, M. Burin, U. Matte, S. Brustolin, et al., Kidney function and 24-hour proteinuria in patients with Fabry disease during 36 months of

agalsidase alfa enzyme replacement therapy: a Brazilian experience, Ren Fail 31 (2009) 773-778.
[59] M. West, K. Nicholls, A. Mehta, J.T.R. Clarke, R. Steiner, M. Beck, et al., Agalsidase alfa and kidney dysfunction in Fabry disease, Journal of the American Society of Nephrology 20(5) (2009) 1132-1139.
[60] F. Breunig, F. Weidemann, J. Strotmann, A. Knoll, C. Wanner, Clinical benefit of enzyme replacement therapy in Fabry disease, Kidney Int 69 (2006) 1216-1221.
[61] M. Goicoechea, F. Gomez-Preciado, S. Benito, J. Torras, R. Torra, A. Huerta, et al., Predictors of long-term outcome in a Spanish cohort of patients with Fabry disease on enzyme replacement therapy, Nephrology Dialysis Transplantation 35(SUPPL 3) (2020) iii363.
[62] S. Hwang, B.H. Lee, W.S. Kim, D.S. Kim, C.K. Cheon, C.H. Lee, et al., A phase II, multicenter, open-label trial to evaluate the safety and efficacy of ISU303 (Agalsidase beta) in patients with Fabry disease, Medicine (United States) 101(37) (2022) E30345.
[63] J.C. Lubanda, E. Anijalg, V. Bzduch, B.L. Thurberg, B. Benichou, A. Tylki-Szymanska, Evaluation of a low dose, after a standard therapeutic dose, of agalsidase beta during enzyme replacement therapy in patients with Fabry disease, Genetics in medicine : official journal of the American College of Medical Genetics 11(4) (2009) 256-264.
[64] U. Ramaswami, D.G. Bichet, L.A. Clarke, G. Dostalova, A. Fainboim, A. Fellgiebel, et al., Low-dose agalsidase beta treatment in male pediatric patients with Fabry disease: A 5-year randomized controlled trial, Molecular Genetics and Metabolism 127(1) (2019) 86-94.
[65] D.G. Warnock, A. Ortiz, M. Mauer, G.E. Linthorst, J.P. Oliveira, A.L. Serra, et al., Renal outcomes of agalsidase beta treatment for Fabry disease: Role of proteinuria and timing of treatment initiation, Nephrology Dialysis Transplantation 27(3) (2012) 1042-1049.
[66] D.G. Warnock, C.P. Thomas, B. Vujkovac, R.C. Campbell, J. Charrow, D.A. Laney, et al., Antiproteinuric therapy and Fabry nephropathy: Factors associated with preserved kidney function during agalsidase-beta therapy, Journal of Medical Genetics 52(12) (2015) 860-866.
[67] F. Weidemann, M. Niemann, S. Stork, F. Breunig, M. Beer, C. Sommer, et al., Long-term outcome of enzyme-replacement therapy in advanced Fabry disease: Evidence for disease progression towards serious complications, Journal of Internal Medicine 274(4) (2013) 331-341.
[68] J.E. Wraith, A. Tylki-Szymanska, N. Guffon, Y.H. Lien, M. Tsimaratos, A. Vellodi, et al., Safety and efficacy of enzyme replacement therapy with agalsidase beta: An international, open-label study in pediatric patients with Fabry disease, Journal of Pediatrics 152(4) (2008) 563-570.e561.
[69] M. Cybulla, K.N. Walter, A. Schwarting, R. Divito, S. Feriozzi, G. Sunder-Plassmann, Kidney transplantation in patients with Fabry disease, Transplant International 22(4) (2009) 475-481.
[70] L. Golan, O. Goker-Alpan, M. Holida, I. Kantola, M. Klopotowski, J. Kuusisto, et al., Evaluation of the efficacy and safety of three dosing regimens of agalsidase alfa enzyme replacement therapy in adults with Fabry disease, Drug Design, Development and Therapy 9 (2015) 3435-3444.
[71] N. Guerard, D. Oder, P. Nordbeck, C. Zwingelstein, O. Morand, R.W.D. Welford, et al., Lucerastat, an iminosugar for substrate reduction therapy: Tolerability, pharmacodynamics, and pharmacokinetics in patients with Fabry disease on enzyme replacement, Clin Pharmacol Ther 103 (2018) 703-711.
[72] D. Hughes, M. Barba Romero, A. Gurevich, P. Engrand, R. Giugliani, Menarche, menopause, and pregnancy data in untreated/agalsidase alfa-treated females in the Fabry Outcome Survey, Twin Research and Human Genetics 21(5) (2019) 439.

[73] T. Prabakaran, H. Birn, B.M. Bibby, A. Regeniter, S.S. Sorensen, U. Feldt-Rasmussen, et al., Long-term enzyme replacement therapy is associated with reduced proteinuria and preserved proximal tubular function in women with Fabry disease, Nephrology Dialysis Transplantation 29 (2014) 619-625.
[74] S.J. van der Veen, S. Korver, A. Hirsch, C.E.M. Hollak, F.A. Wijburg, M.M. Brands, et al., Early start of enzyme replacement therapy in pediatric male patients with classical Fabry disease is associated with attenuated disease progression, Molecular Genetics and Metabolism 135(2) (2022) 163-169.
[75] A.C. Vedder, G.E. Linthorst, G. Houge, J.E. Groener, E.E. Ormel, B.J. Bouma, et al., Treatment of Fabry disease: outcome of a comparative trial with agalsidase alfa or beta at a dose of 0.2 mg/kg, PLoS ONE [Electronic Resource] 2 (2007) e598.
[76] D. Ripeau, H. Amartino, M. Cedrolla, L. Urtiaga, B. Urdaneta, M. Cano, et al., Cambio de agalsidasa beta por agalsidasa alfa en la terapia de reemplazo enzimatica de pacientes con enfermedad de Fabry en Latinoamerica, Medicina (Argentina) 77(3) (2017) 173-179.
[77] P.P. Effati, J.T. Saarinen, E. Loyttyniemi, M. Saarenhovi, R. Autio, I. Kantola, Long-term effectiveness of enzyme replacement therapy in Fabry disease with the p. Arg227Ter (R227*) mutation, Molecular Genetics and Metabolism. Conference: WORLDSymposiumTM 138 (2023).
[78] S. Huang, J. Wang, W. Zhang, F. Gao, Y. Chen, W. Shui, et al., Clinical study of left ventricular structure and function in patients with Anderson-Fabry disease before and after enzyme replacement therapy, J Clin Ultrasound 52 (2024) 20-29.
[79] K. Hongo, K. Ito, T. Date, I. Anan, Y. Inoue, S. Morimoto, et al., The beneficial effects of long-term enzyme replacement therapy on cardiac involvement in Japanese Fabry patients, Molecular Genetics and Metabolism 124(2) (2018) 143-151.
[80] T. Kovacevic-Preradovic, M. Zuber, C.H. Attenhofer Jost, U. Widmer, B. Seifert, G. Schulthess, et al., Anderson-Fabry disease: long-term echocardiographic follow-up under enzyme replacement therapy, Eur J Echocardiogr 9 (2008) 729-735.
[81] M. Lenders, J. Stypmann, T. Duning, B. Schmitz, S.M. Brand, E. Brand, Serum-mediated inhibition of enzyme replacement therapy in Fabry disease, Journal of the American Society of Nephrology 27(1) (2016) 256-264.
[82] H.C. Liu, H.Y. Lin, C.F. Yang, H.C. Liao, T.R. Hsu, C.W. Lo, et al., Globotriaosylsphingosine (lyso-Gb3) might not be a reliable marker for monitoring the long-term therapeutic outcomes of enzyme replacement therapy for late-onset Fabry patients with the Chinese hotspot mutation (IVS4+919G>A), Orphanet Journal of Rare Diseases 9(1) (no pagination) (2014).
[83] S. Nordin, R. Kozor, R. Vijapurapu, J.B. Augusto, K.D. Knott, G. Captur, et al., Myocardial storage, inflammation, and cardiac phenotype in Fabry disease after one year of enzyme replacement therapy, Circulation: Cardiovascular Imaging 12(12) (no pagination) (2019).
[84] C. Schmied, A. Nowak, C. Gruner, E. Olinger, H. Debaix, A. Brauchlin, et al., The value of ECG parameters as markers of treatment response in Fabry cardiomyopathy, Heart. 07 (2016).
[85] F. Weidemann, M. Niemann, F. Breunig, S. Herrmann, M. Beer, S. Stork, et al., Long-term effects of enzyme replacement therapy on Fabry cardiomyopathy: evidence for a better outcome with early treatment, Circulation 119 (2009) 524-529.
[86] A. Camporeale, F. Bandera, M. Pieroni, F. Pieruzzi, M. Spada, A. Bersano, et al., Effect of Migalastat on cArdiac InvOlvement in FabRry DiseAse: MAIORA study, Journal of Medical Genetics 60 (2023) 850-858.
[87] M. Beck, R. Ricci, U. Widmer, F. Dehout, A.G. de Lorenzo, C. Kampmann, et al., Fabry disease: overall effects of agalsidase alfa treatment, Eur J Clin Invest 34 (2004) 838-844.

[88] U. Ramaswami, M. Beck, D. Hughes, C. Kampmann, J. Botha, G. Pintos-Morell, et al., Cardio-renal outcomes with long-term agalsidase alfa enzyme replacement therapy: A 10-year Fabry Outcome Survey (FOS) analysis, Drug Design, Development and Therapy 13 (2019) 3705-3715.
[89] K. Tsuboi, H. Yamamoto, Efficacy and safety of enzyme-replacement-therapy with agalsidase alfa in 36 treatment-naive Fabry disease patients, BMC Pharmacology and Toxicology 18(1) (no pagination) (2017).
[90] A.P. Burlina, M. Mauer, G.H. Cabrera, J.L. Jefferies, M. Yang, E. Ponce, et al., Clinical outcomes in young patients with Fabry disease who initiated agalsidase beta treatment before 30 years of age, Journal of Inherited Metabolic Disease 42(Supplement 1) (2019) 233-234.
[91] P.M. Elliott, H. Kindler, J.S. Shah, B. Sachdev, O.E. Rimoldi, R. Thaman, et al., Coronary microvascular dysfunction in male patients with Anderson-Fabry disease and the effect of treatment with alpha galactosidase A, Heart 92 (2006) 357-360.
[92] D.P. Germain, J. Charrow, R.J. Desnick, N. Guffon, J. Kempf, R.H. Lachmann, et al., Ten-year outcome of enzyme replacement therapy with agalsidase beta in patients with Fabry disease, Journal of Medical Genetics 52 (2015) 353-358.
[93] M. Imbriaco, A. Pisani, L. Spinelli, A. Cuocolo, G. Messalli, E. Capuano, et al., Effects of enzyme-replacement therapy in patients with Anderson-Fabry disease: a prospective long-term cardiac magnetic resonance imaging study, Heart 95 (2009) 1103-1107.
[94] R.J. Kalliokoski, I. Kantola, K.K. Kalliokoski, E. Engblom, J. Sundell, J.C. Hannukainen, et al., The effect of 12-month enzyme replacement therapy on myocardial perfusion in patients with Fabry disease, Journal of Inherited Metabolic Disease 29(1) (2006) 112-118.
[95] G. Messalli, M. Imbriaco, G. Avitabile, R. Russo, D. Iodice, L. Spinelli, et al., Role of cardiac MRI in evaluating patients with Anderson-Fabry disease: assessing cardiac effects of long-term enzyme replacement therapy, La Radiologia medica 117(1) (2012) 19-28.
[96] M. Motwani, S. Banypersad, P. Woolfson, S. Waldek, Enzyme replacement therapy improves cardiac features and severity of Fabry disease, Molecular Genetics and Metabolism 107(1-2) (2012) 197-202.
[97] A. Ortiz, M. Mauer, E. Ponce, M. Yang, B. Gudivada, G.R. Hong, et al., Stabilization of kidney function decline and cardiomyopathy in male patients with classic Fabry disease: A pre-vs. post-agalsidase beta treatment Fabry Registry analysis, Nephrology Dialysis Transplantation 36(SUPPL 1) (2021) i110-i111.
[98] C. Wanner, U. Feldt-Rasmussen, A. Jovanovic, A. Linhart, M. Yang, E. Ponce, et al., Cardiomyopathy and kidney function in agalsidase beta-treated female Fabry patients: a pre-treatment vs. post-treatment analysis, ESC Heart Failure 7(3) (2020) 825-834.
[99] W. Wuest, W. MacHann, F. Breunig, F. Weidemann, H. Koestler, D. Hahn, et al., Right ventricular involvement in patients with Fabry disease and the effect of enzyme replacement therapy, RoFo Fortschritte auf dem Gebiet der Rontgenstrahlen und der Bildgebenden Verfahren 183(11) (2011) 1037-1042.
[100] R. Mignani, S. Feriozzi, A. Pisani, A. Cioni, C. Comotti, M. Cossu, et al., Agalsidase therapy in patients with Fabry disease on renal replacement therapy: A nationwide study in Italy, Nephrology Dialysis Transplantation 23(5) (2008) 1628-1635.
[101] J. Kramer, M. Niemann, S. Stork, S. Frantz, M. Beer, G. Ertl, et al., Relation of burden of myocardial fibrosis to malignant ventricular arrhythmias and outcomes in Fabry disease, Am J Cardiol 114 (2014) 895-900.
[102] C.V. Madsen, H. Bundgaard, A.K. Rasmussen, S.S. Sorensen, J.H. Petersen, L. Kober, et al., Echocardiographic and clinical findings in patients with Fabry disease during long-

term enzyme replacement therapy: a nationwide Danish cohort study, Scandinavian Cardiovascular Journal 51(4) (2017) 207-216.
[103] M. Niemann, F. Breunig, M. Beer, S. Herrmann, J. Strotmann, K. Hu, et al., The right ventricle in Fabry disease: Natural history and impact of enzyme replacement therapy, Heart 96(23) (2010) 1915-1919.
[104] C. Pogoda, S.M. Brand, T. Duning, A. Schmidt-Pogoda, J. Sindermann, M. Lenders, et al., Impact of enzyme replacement therapy and migalastat on left atrial strain and cardiomyopathy in patients with Fabry disease, Front Cardiovasc Med 10 (2023) 1223635.
[105] F. Weidemann, F. Breunig, M. Beer, J. Sandstede, O. Turschner, W. Voelker, et al., Improvement of cardiac function during enzyme replacement therapy in patients with Fabry disease: A prospective strain rate imaging study, Circulation 108(11) (2003) 1299-1301.
[106] M. Arends, F.A. Wijburg, C. Wanner, F.M. Vaz, A.B.P. van Kuilenburg, D.A. Hughes, et al., Favourable effect of early versus late start of enzyme replacement therapy on plasma globotriaosylsphingosine levels in men with classical Fabry disease, Molecular Genetics and Metabolism 121(2) (2017) 157-161.
[107] S. Korver, M.G.F. Longo, M.R. Lima, C.E.M. Hollak, M. El Sayed, I.N. Van Schaik, et al., Determinants of cerebral radiological progression in Fabry disease, Journal of Neurology, Neurosurgery and Psychiatry 91(7) (2020) 756-763.
[108] D.S. Lulla, L. Higuita, S. Shukla-Parekh, H. Lau, Survey assessing the prevalence and severity of neuropsychiatric manifestations in patients living with Fabry disease, Molecular Genetics and Metabolism 129(2) (2020) S103.
[109] A. Nowak, G. Koch, U. Huynh-Do, M. Siegenthaler, H.P. Marti, M. Pfister, Disease progression modeling to evaluate the effects of enzyme replacement therapy on kidney function in adult patients with the classic phenotype of Fabry disease, Kidney and Blood Pressure Research 42(1) (2017) 1-15.
[110] G. Sunder-Plassmann, S. Shankar, W. Wilcox, K. Nicholls, R. Giugliani, H. Lagast, et al., Occurrence of cerebrovascular events during long-term treatment with migalastat in patients with Fabry disease, Journal of Inherited Metabolic Disease 42(Supplement 1) (2019) 233.
